# Clonality analysis of pulmonary tumors by genome-wide copy number profiling

**DOI:** 10.1371/journal.pone.0223827

**Published:** 2019-10-16

**Authors:** Julien P. L. Vincenten, Hendrik F. van Essen, Birgit I. Lissenberg-Witte, Nicole W. J. Bulkmans, Oscar Krijgsman, Daoud Sie, Paul P. Eijk, Egbert F. Smit, Bauke Ylstra, Erik Thunnissen

**Affiliations:** 1 Amsterdam UMC, location VUmc, Department of Pulmonary Diseases, Amsterdam, The Netherlands; 2 Albert Schweitzer Hospital, Department of Pulmonary Diseases, Dordrecht, The Netherlands; 3 Amsterdam UMC, location VUmc, Tumor Genome Analysis Core, Cancer Center Amsterdam, The Netherlands; 4 Amsterdam UMC, location VUmc, Department of Epidemiology and Biostatistics, Amsterdam, The Netherlands; 5 Spaarne Gasthuis, Department of Pathology, Haarlem, The Netherlands; 6 Netherlands Cancer Institute - Antoni van Leeuwenhoek, Department of Molecular Oncology & Immunology, Amsterdam, The Netherlands; 7 Netherlands Cancer Institute - Antoni van Leeuwenhoek, Department of Thoracic Oncology, Amsterdam, The Netherlands; 8 Amsterdam UMC, location VUmc, Department of Pathology, Amsterdam, The Netherlands; National Cancer Center, JAPAN

## Abstract

Multiple tumors in patients are frequently diagnosed, either synchronous or metachronous. The distinction between a second primary and a metastasis is important for treatment. Chromosomal DNA copy number aberrations (CNA) patterns are highly unique to specific tumors. The aim of this study was to assess genome-wide CNA-patterns as method to identify clonally related tumors in a prospective cohort of patients with synchronous or metachronous tumors, with at least one intrapulmonary tumor. In total, 139 tumor pairs from 90 patients were examined: 35 synchronous and 104 metachronous pairs. Results of CNA were compared to histological type, clinicopathological methods (Martini-Melamed-classification (MM) and ACCP-2013-criteria), and, if available, *EGFR*- and *KRAS*-mutation analysis. CNA-results were clonal in 74 pairs (53%), non-clonal in 33 pairs (24%), and inconclusive in 32 pairs (23%). Histological similarity was found in 130 pairs (94%). Concordance between histology and conclusive CNA-results was 69% (74 of 107 pairs: 72 clonal and two non-clonal). In 31 of 103 pairs with similar histology, genetics revealed non-clonality. In two out of four pairs with non-matching histology, genetics revealed clonality. The subgroups of synchronous and metachronous pairs showed similar outcome for the comparison of histological versus CNA-results. MM-classification and ACCP-2013-criteria, applicable on 34 pairs, and CNA-results were concordant in 50% and 62% respectively. Concordance between mutation matching and conclusive CNA-results was 89% (8 of 9 pairs: six clonal and two non-clonal). Interestingly, in one patient both tumors had the same *KRAS* mutation, but the CNA result was non-clonal. In conclusion, although some concordance between histological comparison and CNA profiling is present, arguments exist to prefer extensive molecular testing to determine whether a second tumor is a metastasis or a second primary.

## Introduction

Multiple primary lung cancer (MPLC) may occur synchronous or metachronous. Synchronous primary lung cancer has been reported in a CT screening study in 25% of patients [1], while the incidence of second-primary lung cancer is 2% per year [2,3]. In addition, patients with primary cancer in other sites, in particular head and neck squamous cell cancer, have a high risk of developing second-primary lung cancer [4,5]. In clinical practice it can be challenging to determine, whether multiple tumors in one patient represent metastatic disease or multiple primaries. If the clinical interpretation in patients presenting with multiple pulmonary tumors is ‘metastatic disease’, the treatment will usually be systemic therapy without curative intent [6].

Histological comparison of resection or biopsy specimens combined with clinical features has been proposed to discriminate between multiple primaries from metastases [7,8]. However, this approach has been criticized, as several reports show discordance between clinicopathological criteria and molecular approaches [9–15]. The odds that lesions represent metastatic lesions would increase considerably if clonality could be demonstrated, which is defined as the relation of cells arising from the mitotic division of a single somatic cell [16]. Conversely, non-clonality would argue for multiple primary tumors.

Multiple somatic molecular aberrations have been investigated to determine clonality in daily diagnostic routine: loss-of-heterozygosity (LOH) [17,18], X-chromosome inactivation [19,20], *p53*-, *EGFR-*, *KRAS-*mutations [11–13,21–23] and genome-wide copy number profiling [24–26] or combinations [10,14,15,27,28]. Genome-wide copy number aberration (CNA) analysis by array comparative genomic hybridization (aCGH) has been successfully applied in case reports and small studies for clonality analysis [29–34]. Although the optimal procedure to investigate clonality is not established yet, the latter approach has the advantage of the highest number of data points for clonality analysis. The use of CNA as a more detailed comparison of tumor pairs, is supported by the report by Qiu and colleagues [35], which shows CNA differences, not only between lung adenocarcinoma versus lung squamous cell carcinoma (SqCC), but also between non-small cell lung carcinoma (NSCLC) versus histologically identical tumors from various origins.

To distinguish primary lung cancers from metastatic foci in patients with two lung tumors, the 8^th^ edition of the TNM classification for lung cancer proposes a collective judgment of a multidisciplinary tumor board after taking into account all of the available information, including histologic type, breakpoints in aCGH, radiographic appearance or metabolic uptake, pattern of biomarkers (driver gene mutations), comparison of the rates of growth, presence of nodal or systemic metastases and in cases of resections the appearances in comprehensive histologic assessment [36]. These criteria are divided into clinical (pre-resection) and pathological (post-resection) criteria.

In the 8^th^ TNM classification of lung cancer two approaches are considered sufficiently reliable by themselves to define clonality between two lung tumors: 1) different patterns in histological judgement represent non-clonality, and 2) matching chromosomal breakpoints by DNA sequencing represent clonality.

The purpose of this prospective study is the comparison of genome-wide CNA-profiling to histological and clinicopathological routine procedures for clonality analysis in a cohort of all our patients with synchronous or metachronous tumors of which at least one was located in the lung, and report our results from everyday practice.

## Materials and methods

### Patients and clinical data

All 201 tumors were included from 90 patients who were consecutively diagnosed between 2007 and 2015 with multiple tumors and had at least one tumor located in the lung, resulting in 139 tumor pairs (Table 1), of which 25% was synchronous. The median age of patients was 60 (range 25–82). The metachronous pairs had a median interval of 30 months between diagnosis (range 1–252 months). Formalin fixed, paraffin embedded (FFPE) resection specimens, as well as small samples (biopsies or fine-needle aspirations) were used. The request to perform clonality analysis was made by the treating pulmonologists or pathologist.

**Table 1:** Clinicopathological information categorized for patients, tumor samples and pairs.

**Patients** **n = 90**	**Gender**			Male		50 (56%)
				Female		40 (44%)
	**Number of tumors per patient**			2		73
				3		15
				4		1
				6		1
**Samples** **n = 201**	**Location of the tumor**	**Intrathoracic**		Intrapulmonary		113 (56%)
				Mediastinum	Tumors	3
					Lymph nodes	5
				Pleura / Diaphragm		3
		**Extrathoracic**	H&N* (17%)	Tumors		30
				Lymph nodes		5
			**Other** (21%)	Axillary lymph nodes		1
				Digestive system		15
				Breast		6
				Urinary system and male genital organs		8
				Female reproductive organs		3
				Central nervous system		2
				Soft tissue or bone		6
				Skin		1
	**Sample type**			Resection		108 (54%)
				Small samples	Biopsy	88 (44%)
					Cytology	5 (2%)
	**Histology**			Adenocarcinoma		70 (35%)
				Squamous cell carcinoma		90 (45%)
				Adenosquamous		2 (1%)
				Undifferentiated non-small cell carcinoma		12 (6%)
				Small cell carcinoma		5 (2%)
				Pulmonary carcinoid tumor		9 (4%)
				Other		13 (6%)
**Pairs** **n = 139**	**Sample types per pair**			Resection vs resection		58 (42%)
				Resection vs small sample		55 (40%)
				Small sample vs small sample		26 (19%)
	**Interval**			Synchronous		35 (25%)
				Metachronous**	<2 years	50 (36%)
					2–4 years	32 (23%)
					≥4 years	22 (16%)
	**Histology**			Match		130 (94%)
				No match		9 (6%)
	**Martini-Melamed**			Intrapulmonary metastatic		17
				Multiple primary lung cancer		24
				Not applicable^#^		98
	**ACCP-2013**			Intrapulmonary metastatic		11
				T3		10
				T4		8
				Multiple primary lung cancer		12
				Not applicable^#^		98
	**Mutation analysis**			Match		8
				No match		3
				Not performed on both samples		128
	**CNA**			Clonal		74 (53%)
				Non-clonal		33 (24%)
				Inconclusive		32 (23%)

*H&N = head and neck

**Metachronous: median time of interval: 30 months (range 1–252)

^#^ Martini-Melamed and ACCP-2013 are used for comparison of intrapulmonary tumors. Not applicable implies at least one of the tumors is not inside the lungs.

Histopathological analysis was performed by central review (ET). The histological outcomes were categorized according to the WHO classification(s) into major histological types, which are recognized across different organ systems: adenocarcinoma, SqCC, adenosquamous carcinoma, undifferentiated non-small cell carcinoma, small cell carcinoma, carcinoid, large cell neuroendocrine carcinoma and ‘other’. Within a category we aimed for agreement in patterns. Immunohistochemistry (IHC) was applied if necessary, especially in case of biopsies and cytological samples. TTF-1, mucin and p63 were used for biopsies and cytology samples with undifferentiated non-small cell carcinoma to discriminate between NSCLC favoring adenocarcinoma (TTF-1 or mucin positive, p63 negative), favoring SqCC (TTF-1 and mucin negative, p63 positive), NSCLC not otherwise specified (NOS) (TTF-1, mucin and p63 negative) and NSCLC NOS, possible adenosquamous carcinoma (TTF-1/mucin and p63 positive) [37–39]. The samples with NSCLC favoring SqCC (n = 6) and NSCLC favoring adenocarcinoma (n = 1) were assigned to the groups of SqCC and adenocarcinoma respectively. The NSCLC-NOS samples (n- = 5) and samples with missing IHC data (n = 2) were added to the group of undifferentiated non-small cell carcinomas [40]. The use of other markers could be applied in specific cases if a primary other organ was considered, but was not a requirement for inclusion in the study. Subtyping of adenocarcinomas was not performed [41]. Pairs with similar histology were categorized as ‘histological matching’. In a minority of the pairs *EGFR*- and *KRAS*-mutation analysis was performed on both tumors and available for comparison.

Clinical data were retrospectively collected for an additional research question about the correlation between clonality analysis and the occurrence of (extra) metastases after CNA. Follow-up data were analyzed for signs of metastatic cancer without knowledge of the results of clonality analysis. Metastatic disease was defined radiologically as a pattern of spread of tumors in multiple organs, associated with metastases (lungs, liver, brain, bones and adrenal glands) [42].

### Martini–Melamed (MM-) criteria

For pairs with both tumors located in the lungs, categorization was performed by MM-criteria to differentiate MPLC from intrapulmonary metastases [7].

### ACCP-2013 criteria

The latest guideline from the American College of Chest Physicians (ACCP) regarding multiple lung tumors defines multiple primary lung cancer in three ways: 1) tumors with the same histology, located in a different lobe and no N2 or N3 lymph node involvement and no systemic metastases, 2) tumors with a different histology, molecular genetic characteristics or arising from separate foci of carcinoma in situ, 3) tumors with the same histology with at least a 4-year interval and no systemic metastases [8]. Other tumor pairs are considered to be related, which are categorized as T3, T4 or pulmonary metastases. T3 means tumors in the same lobe with the same histology. T4 means histologically similar tumors, which are anatomically separated and located in different ipsilateral lobes. Pulmonary metastases are defined in three ways: 1) tumors with similar histology and multiple systemic metastases, 2) tumors with similar histology, located in different lobes and signs of N2 or N3 involvement, 3) tumors with an interval of less than two years.

### Genome-wide CNA analysis by aCGH

CNA profiling to assess clonality was performed on DNA isolated from formalin fixed and paraffin embedded (FFPE) samples at the Department of Pathology of the Amsterdam UMC, location VUmc, which is ISO 15189 accredited (previously CCKL https://www.cckl.nl/).

Two or more synchronous or metachronous FFPE tumor samples from a single patient were offered for a clonality test upon clinical request. In metachronous cases FFPE blocks of the first tumor were retrieved from the archives of the pathology department. Non-malignant tissue was used as reference source for DNA CNA analysis. From each FFPE block an initial 5μm section was cut for Haematoxylin and Eosin (H&E) staining. Sequentially, 10 sections of a thickness of 10μm were cut, tumor material was macroscopically selected and genomic DNA was isolated as previously described [43]. After DNA isolation, samples were labelled according to the manufacturer’s instructions (Enzo Life Sciences, New York, USA), before hybridization to aCGH slides (Agilent Technologies, Amstelveen, NL) [44]. Since the start of the study the number of probes per array on the CGH slides increased from a 105K to a 180K, as shown in Table 2. The laboratory protocols are made publicly available at protocols.io (dx.doi.org/10.17504/protocols.io.zj7f4rn; dx.doi.org/10.17504/protocols.io.zj3f4qn; dx.doi.org/10.17504/protocols.io.zjuf4nw; dx.doi.org/10.17504/protocols.io.zjwf4pe). All array designs and aCGH data, including the unsuccessful experiments, are made publicly available in GEO under accession number GSE87058.

**Table 2:** The number of submitted tumor pairs categorized per year and used aCGH platform. Note the increase of the number of CGH probes over time.

Year	4x44k	2x105k	4x180k	No aCGH*	Total
2007	6				6
2008		2			2
2009		11	10		21
2010		1	17	4	22
2011		1	32		33
2012			12		12
2013			26		26
2014			16	1	17
Total	6	15	113	5	139

* No aCGH performed due to insufficient DNA-quality or lack of remaining tumor tissue.

In addition, all CNA profiles are added in pairs, as supplementary figures (S1 Figures).

#### Data analysis

Pre-processing of the CNA data was executed as previously described by van de Wiel et al. [45], which applies several R-packages version R-2.10.1. Segmentation was performed using Bioconductor R-package DNA copy version 1.20.0, which data were used to determine clonality based on two independent algorithms. First, the Bioconductor R-package “Clonality” version 1.0.0 was implemented with defaults settings and cut-off values as described in Ostrovnaya et al. [46], which calculates a likelihood ratio (LR) based on the concordance of the segment values of the CNAs in the tumor. For the LR the non-clonal result is a negative number and a clonal result is a positive number.

Secondly, we developed a correlation-based method to calculate clonality. This ‘Pearson correlation’ was also calculated based on the log2ratio values of the CNA segment values.

To determine a cut-off for classification of tumor profiles as clonal or non-clonal, 626 random pairs were generated from a pool of publicly available CNA profiles, GEO accession number GSE38479, each from a separate patient [47]. This set of non-clonal tumor pairs was used in a cross validation to calculate a cut-off value by which 95% of (non-clonal) pairs was classified as non-clonal. The cross validation was performed using randomly selected sets of 2/3 of the sample pairs, the cut-off was set where 95% of samples were classified as non-clonal. This process was iterated 10,000 times. As expected, the 10,000 cut-off values from the cross validation followed a normal distribution after which the final cut-off was set at the mode of this normal distribution. In practice, if the Pearson correlation, based on segmented log2ratios, is below <0.54, it is considered non-clonal, whereas it is considered clonal when the result is >0.54.

A visual analysis was independently performed by two experts (B.Y. & E.T.). A moving average was performed, if necessary for visualization purposes [48]. CNA profile pairs were categorized as clonal or non-clonal. Alternatively, some clonality comparisons were visually determined as inconclusive if a low deflection from the normal was observed in one or both profiles. Note that, although tumor cell percentage was estimated prior to DNA isolation from the H&E sections by the Pathologist (ET), this was not included in the clonality calculations. Visually incongruent tumor pairs and inconclusive pairs were discussed, and consensus was obtained. Inconclusive results were invariably due to insufficient quality of the CNA profiles [43], which we suspect is due to degraded DNA isolated from these clinical tumor specimens or a low fraction of neoplastic cells, since repetition of these experiments did not improve results.

The mathematical and visual evaluation were combined for the final CNA result.

All protocols to obtain and study human archived tissues and patients’ data were approved by the Medical Ethics Review Committee (METc) at the VU University Medical Center and in compliance with the Code for Proper Secondary Use of Human Tissue in The Netherlands. The Medical Research Human Subjects Act (WMO in Dutch) does not apply as affirmed 2019.455. The data from the patient records for the retrospective part of our study, were retrieved by one researcher and anonymized before any other involved researcher could access them.

*EGFR*- and *KRAS*-mutation analyses were performed as described before [49].

The relations between histology matching, Martini-Melamed criteria, ACCP-2013-criteria, CNA-results and mutation analysis matching were separately assessed with the McNemar test, SPSS version 22 (BW). A *p*-value <0.05 was considered significant.

## Results

The predominant histological types were SqCC (45%, 90/201 tumors) and adenocarcinoma (35%, 70/201 tumors). The histological diagnoses matched in 94% of the tumor pairs (130/139 pairs). CNA results showed clonality in 53% (74/139 pairs) and a non-clonal relation in 24% (33/139 pairs). Inconclusive outcomes for CNA profiles accounted for 23% (32/139 pairs) and were most frequently a result of insufficient DNA and subsequent CNA quality (91%). The highest percentage of inconclusive CNA-results was in the group of ‘resection versus small sample’ pairs: 35% (19/55 pairs), compared to 19% (11/58 pairs) of the ‘resection versus resection’ pairs and 8% (2/26 pairs) of the ‘small sample versus small sample’ pair (*p* = 0.017).

The clinical indication for clonality analysis was the assessment of intrapulmonary tumors (29%, 41/139 pairs), a lung tumor versus a head & neck tumor (23%, 32/139 pairs), and a lung tumor versus tumors from other locations (47%, 66/139 pairs; Table 3).

**Table 3:** CNA compared to other methods for clonality analysis. Highlighted in grey are discordant results. P-values from McNemar test.

		CNA				
		Clonal (n = 74)	Non-clonal (n = 33)	Inconclusive (n = 32)	Total (n = 139)	*p*-value
**Histology**	Match	72	31	27	130	<0.001
	No match	2	2	5	9	
**Martini-Melamed**	Metastasized	10	3	4	17	0.015
	MPLC	14	7	3	24	
	Not applicable^#^	50	23	25	98	
**ACCP-2013**	Related*	17	6	6	28	1.00
	MPLC	7	4	1	13	
	Not applicable^#^	50	23	25	98	
**Mutation analysis**	Match	6	1	1	8	1.00
	No match	0	2	1	3	
	Missing	68	30	30	128	

MPLC = multiple primary lung cancer

* Related: metastatic, T3 or T4

^#^ Martini-Melamed and ACCP-2013 are used for comparison of intrapulmonary tumors. ‘Not applicable’ implies at least one of the tumors is not inside the lungs.

A special carcinoid subgroup comprised nine tumors from four patients. Patient 1 had a lung carcinoid and a thoracic wall carcinoid with an interval of eleven years, and a third carcinoid on the forehead another three years later. Patient 2 had a lung carcinoid and a pleural carcinoid with an interval of twenty years. Patient 3 had a lung carcinoid and an ipsilateral carcinoid in the other lobe with an interval of eight years. Patient 4 had a stomach carcinoid and a lung carcinoid one year later. The CNA-results for all carcinoid pairs were clonal, except for the stomach-lung tumor pair, which was inconclusive.

### Histology versus CNA profiling

**Fig 1:**
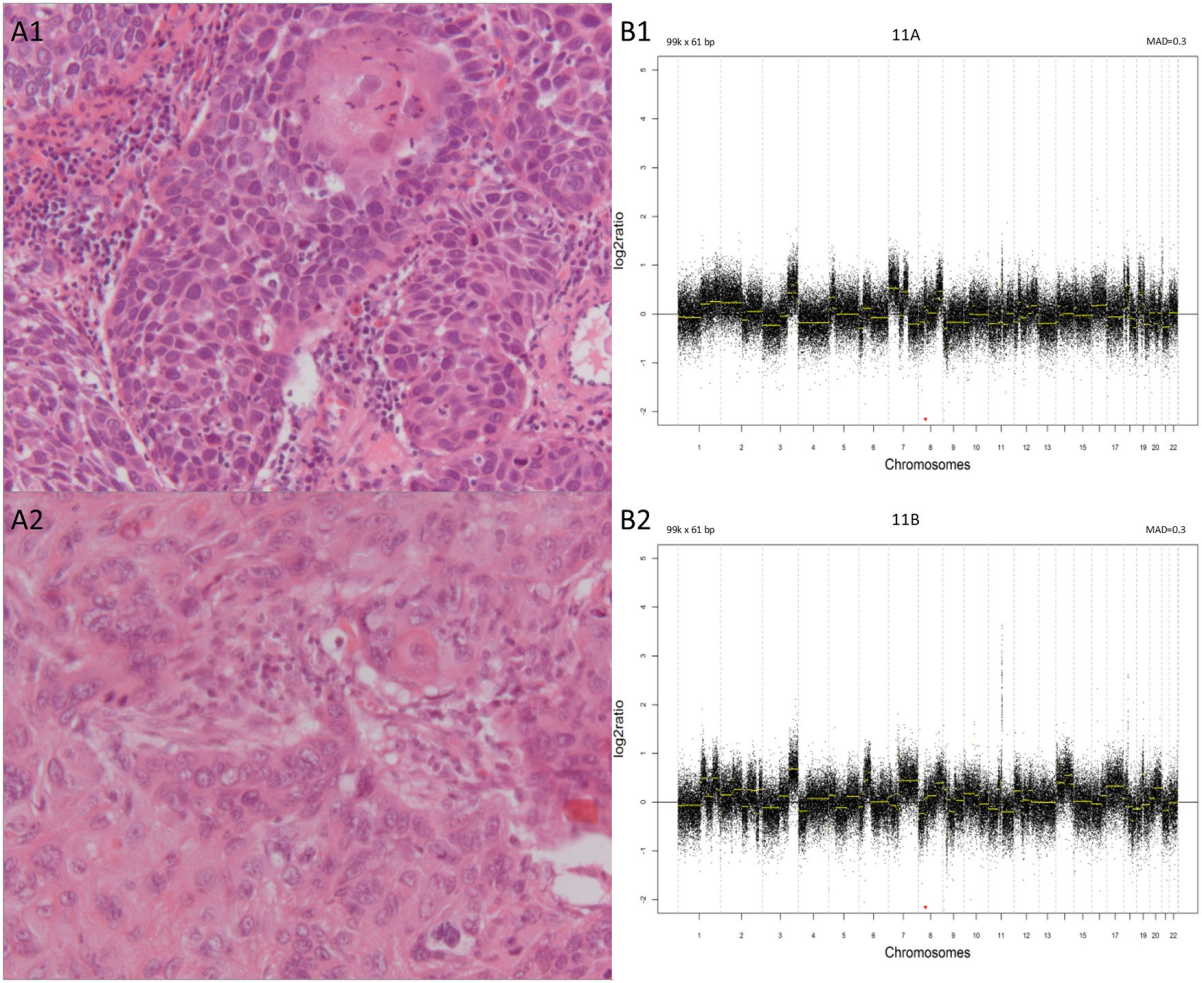
Example of a matching histologic pair with a non-clonal CNA-result. A1 & A2: squamous cell carcinomas from a laryngeal tumor biopsy and a resected tumor in the left lower lobe, respectively (20x objective). B1 & B2: the corresponding CNA profiles. On the y axis is the log2 tumor to normal ratio and on the x axis the chromosomal position. MAD = median absolute deviation.

**Fig 2:**
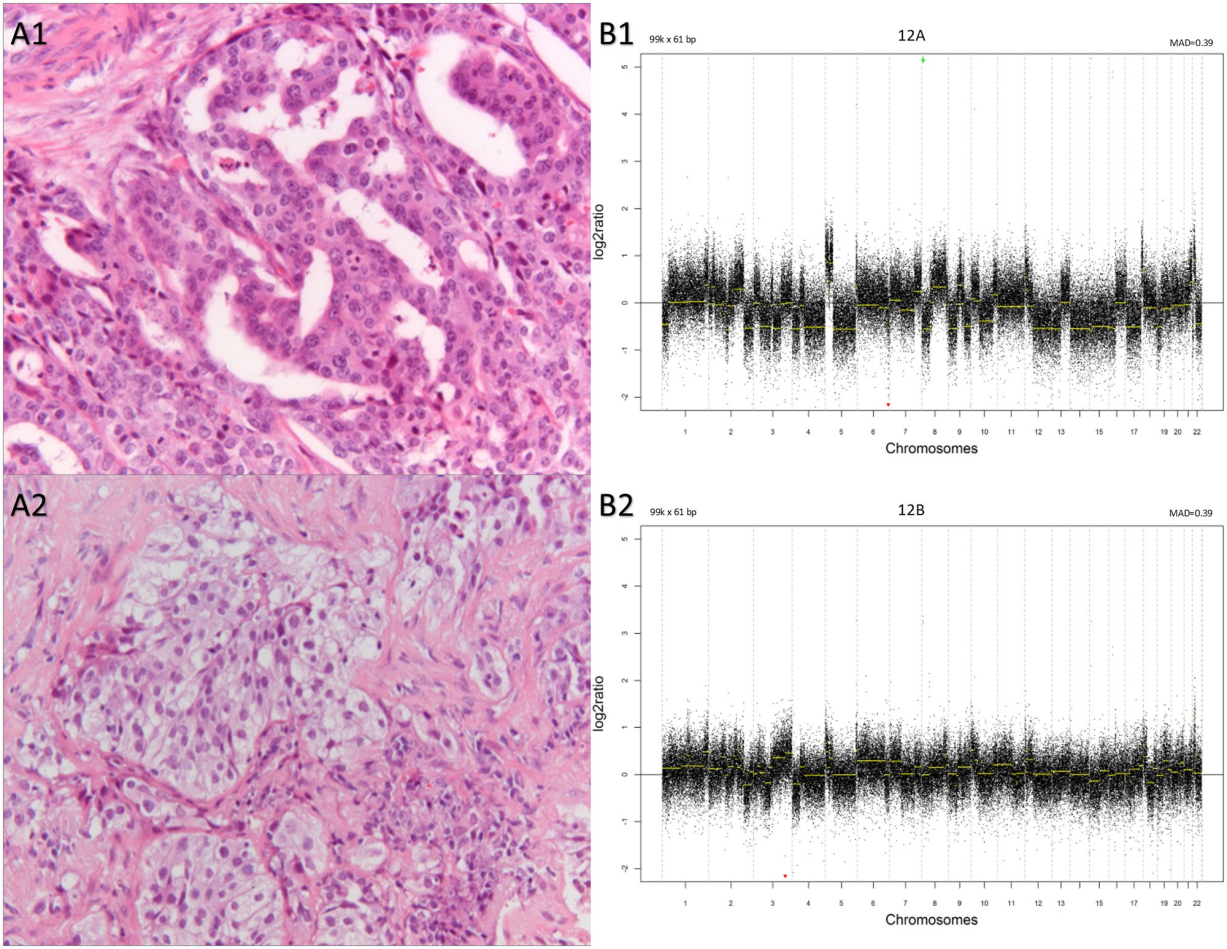
Example of a non-matching histologic pair with a clonal CNA-result. A1: adenocarcinoma from a breast resection. A2: non-small cell carcinoma, favoring squamous cell carcinoma (IHC: p63 positive; TTF-1/PAS-D/Alcian blue negative) from a lung biopsy (20x objective). B1 & B2: the corresponding CNA profiles. On the y axis is the log2 tumor to normal ratio and on the x axis the chromosomal position. Gain and loss are positive and negative log2 ratio, respectively. MAD = median absolute deviation. All array data are available in the Gene Expression Omnibus database, under accession number GSE87058. Mutation analysis: not performed on both samples. Martini-Melamed and ACCP-2013: not applicable. Follow-up: no sign of metastasis after 79 months.

Comparison of histological matching with conclusive CNA analysis (77% of total; 53% clonal, 24% non-clonal) revealed concordance in 69% (74/107 pairs: 72 clonal and two non-clonal) (*p* < 0.001; Table 3). CNA showed a non-clonal pattern in 30% of the pairs with matching histology (31/103 pairs; example in Fig 1), while a clonal pattern was present in 50% of the pairs with different histology (2/4 pairs; example in Fig 2). The cases with a clonal CNA result and different histology were 1) a biopsy with NSCLC-favor SqCC (immunohistochemistry positive for p63 and negative for TTF-1) versus a resection specimen with adenocarcinoma and 2) biopsies of two lung tumors in the same lobe, showing a NSCLC-NOS (TTF-1 and p63 negative) and NSCLC-favor SqCC (TTF-1 negative, p63 positive).

Synchronous and metachronous tumor pairs present two different clinical entities. Results were categorized for these subgroups (Table 4).

**Table 4:** Histology versus CNA-results for the subgroups of synchronous and metachronous tumor pairs.

			CNA			Total	*p*-value
			Clonal	Non-clonal	Inconclusive		
**Synchronous** (n = 35)	Histology	Match	18 (56%)	8 (25%)	6 (19%)	32	0.046
		Non-match	1 (33%)	1 (33%)	1 (33%)	3	
**Metachronous** (n = 104)	Histology	Match	54 (55%)	23 (23%)	21 (22%)	98	<0.001
		Non-match	1 (17%)	1 (17%)	4 (67%)	6	

Comparison of the results of histological matching from synchronous tumor pairs (n = 35) with conclusive CNA-results (80% of total; 54% clonal, 26% non-clonal) revealed concordance in 71% (19/28 pairs: 18 clonal and one non-clonal) (*p* = 0.046; Table 4). CNA showed a non-clonal pattern in 31% of the pairs with matching histology (8/26 pairs), while a clonal pattern was present in one of the two pairs with different histology.

For the group of metachronous tumor pairs (n = 104) comparison of the results of histological matching with conclusive CNA-results (76% of total; 53% clonal, 23% non-clonal) revealed concordance in 70% (55/79 pairs: 54 clonal and one non-clonal) (*p*<0.001; Table 4). CNA showed a non-clonal pattern in 30% of the pairs with matching histology (23/77 pairs), while a clonal pattern was present in one of the two pairs with different histology.

### Clinicopathological classifications for intrapulmonary tumor pairs versus CNA profiling

Results of the MM-classification compared to conclusive CNA-results were concordant in 50% (17/34 pairs: ten clonal/intrapulmonary metastasized and seven non-clonal/MPLC) (*p* = 0.015; Table 3).

Results of the ACCP-2013 classification compared to conclusive CNA profiling-results were concordant in 62% (21/34 pairs: 17 clonal/related (five intrapulmonary metastasized, eight T3, four T4) and four non-clonal/MPLC) (*p* = 1.00; Table 3).

### Mutation analysis matching versus CNA profiling

As *EGFR-* and *KRAS-*mutation analyses were not standard procedures in this study, but only performed upon clinical request for treatment decisions, results of mutation analyses on both tumors of a pair were available for only eleven pairs (Table 5). A pair with an inconclusive CNA-result was found in the group with matching mutations, as well as in the group with non-matching mutations. In the remaining pairs with conclusive CNA-results mutation matching was concordant in 89% (8/9 pairs): six clonal and two non-clonal (*p* = 1.0) (Table 3). Even though one pair showed a matching mutation, suggesting a clonal relationship, clear discordance in the CNA-profiling result was present.

**Table 5:** Details of pairs (n = 11) with mutation analysis performed upon both tumor samples.

Pair	Mutation analysis*	Sample types**	Locations^$^	Interval (months)	CNA	Histology^‡^	Martini-Melamed^†^	ACCP-2013^†^	Follow-up
**Patient 1, pair 1**	No match (WT vs KRAS c.35G>T; p.G12V)	R-R	RLL vs RML	0	Non-clonal	Match (ADC)	MPLC	MPLC	Metastases
**Patient 1, pair 2**	No match (KRAS c.34G>T; p.G12C vs WT)	S-R	Gluteus vs RLL	2	Non-clonal	Match (ADC)	NA	NA	Metastases
**Patient 1, pair 3**	No match (KRAS c.34G>T; p.G12C vs c.35G>T; p.G12V)	S-R	Gluteus vs RML	2	Inconclusive	Match (ADC)	NA	NA	Metastases
**Patient 2**	Match (KRAS c.35G>A; p.G12D)	R-R	LUL vs LUL	0	Clonal	Match (ADC)	Metastatic	T3	No metastases (FU 7 months)
**Patient 3**	Match (KRAS c.34G>T; p.G12C)	R-R	RUL vs RUL	0	Clonal	Match (ADC)	Metastatic	T3	No metastases (FU 40 months)
**Patient 4, pair 1**	Match (EGFR exon 21, (c.2573 T>G; p.L858R) vs EGFR exon 21 (c.2573 T>G; p.L858R), plus EGFR exon 20 (c.2369 C>T; p.T790M)	R-R	RUL vs LUL	75	Clonal	Match (ADC)	MPLC	MPLC	Metastases
**Patient 4, pair 2**	Match (EGFR exon 21 (c.2573 T>G; p.L858R), plus EGFR exon 20 (c.2369 C>T; p.T790M)	R-R	LUL vs LUL	0	Clonal	Match (ADC)	Metastatic	T3	Metastases
**Patient 4, pair 3**	Match (EGFR exon 21, (c.2573 T>G; p.L858R) vs EGFR exon 21 (c.2573 T>G; p.L858R), plus EGFR exon 20 (c.2369 C>T; p.T790M)	R-R	RUL vs LUL	75	Clonal	Match (ADC)	MPLC	MPLC	Metastases
**Patient 5**	Match (KRAS c.35G>T; p.G12V)	R-S	RUL vs Stomach	19	Clonal	Match (ADC)	NA	NA	Metastases
**Patient 6**	Match (KRAS c.34G>T; p.G12C)	S-S	LLL vs RLL	0	Non-clonal	Match (ADC)	Metastatic	Metastatic	Metastases
**Patient 7**	Match (EGFR exon 19 deletion (c.2235-2249del15, p.DelE746-A750) plus exon 20 (c.2369 C>T; p.T790M)	R-R	RUL vs RLL	0	Inconclusive	Match (ADC)	MPLC	T4	Metastases

* WT: wild type

** Samples: R = resection, S = small sample

^$^ Locations: RLL = right lower lobe, RML = right middle lobe, LUL = left upper lobe, RUL = right upper lobe, LLL = left lower lobe, RLL = right lower lobe

^‡^ Histology: ADC = adenocarcinoma, LCC = large cell carcinoma

^†^ NA = not applicable. Martini-Melamed and ACCP-2013 are used for comparison of intrapulmonary tumors. “Not applicable’ implies that at least one of the tumors is not inside the lungs.

### Follow-up data versus CNA and (clinico)pathological results

Follow-up data showed metastatic disease in 57% (79/139 pairs) and no metastatic disease in 26% (36/139 pairs). Six percent (9/139 pairs) was excluded from the follow-up data analysis, because the patients had died within six weeks after the CNA-analysis. Missing follow-up data accounted for 11% (15/139 pairs). The follow-up period for pairs without signs of metastatic disease ranged from two to 102 months (median 26 months) and was less than five years in 81% (29/36 pairs). The follow-up period of less than five years was in 41% due to non-cancer-related death (12/29 pairs) and for the other 59% the endpoint of our study was less than five years after the date of CNA-profiling. The follow-up data are shown in Table 6, categorized by method for clonality analysis. The CNA clonal group showed signs of clinical metastatic disease in 70% (43/61 pairs), which was similar to the percentage in the non-clonal group (19/27 pairs; *p* = 1.00). The histologically matching group showed signs of metastatic disease in 71% (77/109 pairs), compared to 33% in the group with different histology (2/6 pairs) (*p*<0.001). The MPLC-group according to MM-criteria showed metastatic disease in 67% (14/21 pairs), compared to 71% in the group with intrapulmonary metastasized lung cancer (12/17 pairs) (*p* = 0.07). The non-clonal group according to the ACCP-2013-criteria showed metastatic disease in 67% (6/9 pairs), compared to 69% in the group with related tumors (20/29 pairs) (*p* = 0.61).

**Table 6:** Follow-up data categorized by method for clonality analysis.

		Metastatic disease during follow-up		
		Yes (n = 79)	No* (n = 36)	*p*-value
**Histologic types**	Match	77	32	<0.001
	No match	2	4	
***EGFR/KRAS*-mutations**	Match	6	2	1.00
	No match	3	0	
**Martini-Melamed**	Metastasized	12	5	0.067
	MPLC	14	7	
**ACCP-2013**	Related^#^	20	9	0.61
	MPLC	6	3	
**CNA**	Clonal	43	18	1.00
	Non-clonal	19	8	

* Range of the follow-up period: 2 to 102 months (median 26 months)

^#^ Related: metastatic, T3 or T4

## Discussion

In this prospective cohort, in 77% of the tumor pairs a conclusive test result for clonality analysis was obtained by genome-wide CNA profiling, of which 69% was clonal (i.e. metastases) and 31% non-clonal (i.e. multiple primary tumors). The comparison of CNA-results with histological matching showed a concordance rate of 69%. The difference in outcomes was statistically significant (*p*<0.001). In 24% of the tumor comparisons with similar histology a different molecular pattern was revealed by CNA profiling. Moreover, a matching molecular CNA pattern was shown in some tumors (22%) with different histology. In other tumor types similar results are reported for comparison of tumors with different histology [33,50,51], as well as for comparison of tumors with similar histology [34,35].

Our data show less concordance than some reports on molecular methods and histological matching including histological subtyping [24,52–54], but are in line with another [55].

We performed the histological evaluation into major categories: adenocarcinoma squamous cell carcinoma and undifferentiated to cover more than one WHO classification. For lung cancer cases we also applied IHC if necessary [38,39]. The use of other markers was allowed, but was not a requirement for inclusion in the study. Moreover, the specificity of IHC is not always useful for distinction: e.g. TTF1 CK7, CDX2 and CK20 do not have any distinctive capacity between primary intestinal carcinoma from the lung and a metastasis from a colorectal carcinoma, as the IHC pattern is the same: TTF1-, CK7-, CDX2+ and CK20+ [40].

In lung cancer studies with sufficiently detailed information the reported discordance rates between histological and molecular prediction range from 9–46% [10,22,54,55]. In these studies only lung tumors were compared to each other, while in our study also tumors from other sites are compared to intrapulmonary tumors. A recent histopathological reproducibility study in NSCLC shows an agreement rate of 81% for determining primary tumor or metastases, based on histology alone [56]. However, this study did not use molecular analysis for confirmation of these findings.

Application of the clinical ‘proposed criteria to distinguish separate primary lung cancers from metastatic foci in patients with two lung tumors in the 8^th^ edition of the TNM classification of lung cancer’ was suitable for only a subset of intrapulmonary tumor pairs in our cohort (n = 41) [36]. As stated before, only two criteria are decisive themselves: 1) different patterns in histological judgement prove non-clonality, and 2) matching chromosomal breakpoints by DNA sequencing demonstrate clonality. Based on the histologic types, 7% of our pairs showed non-matching results (3/41). These cases would be classified as non-clonal. The remaining cases would require further evaluation (93%; 38/41).

Interestingly, one of the three tumor pairs with dissimilar histologic types showed a similar CNA pattern, which means that, according to the proposed criteria, this tumor pair could be classified as non-clonal (based on histology), as well as clonal (based on genetics). The remaining cases with dissimilar histologic types showed one non-clonal and one inconclusive CNA results. After application of the proposed decisive criteria on our cohort, comparison of the histologic types would not be decisive in 93% and in the remaining 7% of cases it would contradict CNA results in at least one case. These findings emphasize the current need for the proposed collective judgment of a multidisciplinary tumor board, but they also emphasize the desirability of better objective, decisive methods than the proposed ones.

The subgroups of synchronous and metachronous pairs showed similar results. CNA results were conclusive in 80% and 76% respectively. Concordance between histological similarity and CNA results was 68% and 70% respectively. In respectively 33% (1/3) and 17% (1/6) CNA revealed clonality in pairs with different histology. In respectively 25% (8/32) and 23% (23/98) of pairs with similar histology a different molecular pattern was revealed by CNA profiling in both groups.

In 1975 Martini and Melamed introduced clinicopathological criteria (MM) to define multiple primary lung tumors [7]. The guideline of the ACCP recommends adjusted clinicopathological criteria [8]. In our study, comparison of CNA-results with MM and ACCP-2013 approaches showed discordance in 50% and 38%, respectively. In other studies, comparison of the MM-criteria with molecular approaches showed discordance ranging from 14 to 43% [9,57]. In essence, the clinicopathological approach for the distinction between primary tumors and metastases in patients with multiple lung tumors is not confirmed by molecular analysis in approximately half of the comparisons. Overall, molecular analysis outperforms the clinicopathological approach.

Until recently, the hypotheses existed that differences in driver mutations between two pulmonary tumors provide a strong argument for two primary tumors, while the same driver mutations point to lineage. These hypotheses were mainly based on studies which examined tumors by mutation analysis only [11–13,22,23,58]. Although only a limited number of cases in our study were available for the comparison of the results of CNA-profiling and mutation analysis, the concordance between the two methods was high. One pair with the same *KRAS*-mutation in both tumors showed a discordant CNA-result, i.e. a non-clonal relationship. A pair with corresponding *KRAS*-mutations and non-clonal CNA-patterns can be explained by the fact that missense *KRAS*-mutations have a relatively high incidence in NSCLC. The value of corresponding single mutations for clonality analysis depends on the frequency of these mutations: rare mutations have a very low probability of co-occurrence in separate tumors by incidence, implying that corresponding rare mutations are stronger arguments for clonality than correspondence for more frequently occurring mutations.

In Western NSCLC the frequency of *KRAS* p.G12C mutations ranges from 8–14% [59–61]. Extrapolating this to a random NSCLC pair leads to the odds of 1% of having the same *KRAS* p.G12C mutation. It is not excluded that the odds may be higher for separate tumors within one patient, as the same carcinogenic exposure is involved and genetic variations in xenobiotic metabolism and DNA repair enzymes are excluded.

The use of identical *KRAS*-mutations as argument for clonality should therefore be interpreted with caution. Previously, a similar finding has been described for *EGFR*: arguments for clonality were based on identical *EGFR*-mutations, while a parallel performed alternative method revealed arguments against clonality [24]. In two additional recent lung cancer heterogeneity studies, comparable information was obtained from three out of 13 cases in total [62,63]. Overall, the hypothesis that identical driver mutations equal a clonal relationship has been falsified in four studies. Therefore, identical driver mutations can no longer serve as proof for lineage between tumors, particularly not if only one single gene is analysed. NGS Multi-Gene mutation panels provide more datapoints for comparison, and lineage for these results is likely to provide more evidence for clonality than single gene mutation analysis. In SqCC CNA may be more suitable than comparison of mutations for clonality analysis, as less clinically relevant driver mutations are known in SqCC than in adenocarcinoma [64].

Intratumoral heterogeneity makes the comparison of the accuracy of the various available molecular methods challenging [65,66]. Tumors originating from a single cancer (stem) cell can still have marked differences due to heterogeneity, which can obscure clonality assessments. As methods for clonality analysis may have conflicting results, the question arises as to which method provides the correct results. The lack of a gold standard makes it difficult to establish the accuracy of the methods for clonality analysis. As long as no gold standard is available, it is reasonable to prefer the method, which provides the most detailed information, i.e. the largest number of data points. Molecular methods study tumors in more detail than morphological or clinicopathological methods and may therefore be considered as more accurate.

A limitation of our study is that CNA-profiling was inconclusive in 23% of the pairs, even though this is in line with initial CNA reports, ranging from 0–25% [10,24,35], and in line with other clinical DNA techniques, like mutation analysis [67–70]. Inconclusive results were caused by a high background noise in the CNA-profiles, possibly due to reduced labelling efficiency of low FFPE-isolated DNA [44]. Another limitation is in copy number analysis by aCGH (or other molecular methods) a sufficient number of genetically aberrant (tumor) cells need to be present in the tissue selected for DNA isolation. For aCGH at least 10% tumor cells is required for CNA detection [71]. Since 2015, CNA profiling by shallow next generation sequencing (NGS) has replaced aCGH in our clinical diagnostic setting. Shallow NGS is more robust, i.e. with significantly less to no inconclusive cases [34,72–74]. Moreover, since April 2017 CNA profiling runs in parallel with a non-invasive prenatal test [75], leading to a reduced current turnaround time of six working days.

Other limitations of our study are the retrospective method of the collection of the follow-up data and the considerable loss to follow-up (11%).

Follow-up data have limitations as a standard to assess the accuracy of molecular methods in the clinical context of two tumors. If metastases develop in time, this may be related to one or both initial tumors. The absence of metastatic disease during follow-up does not exclude a clonal relationship between two radically treated tumors either, as radical treatment improves clinical outcomes in oligometastatic disease [76,77]. Therefore, the results of follow-up are not decisive about clonality between tumors. Differences in our follow-up results were found between histological similar and different pairs (*p*<0.001), but not between the CNA-clonal group and the CNA-non-clonal group (*p* = 1.00), and neither between MM-categories MPLC and intrapulmonary metastasized disease (*p* = 0.067), nor between the ACCP-2013 related and non-related groups (*p* = 0.61).

When tumors are clonal and the patient is therefore diagnosed with the metastatic disease, it might be expected that the follow-up would show new metastases in nearly 100% of cases. Our results showed new metastases in ‘only’ 70% of CNA clonal cases. This paradoxical appearance may be due to the fact that our group of clonal cases is not an average cohort of metastatic (frequently lung) cancer patients, as many stage IV cancer patients have signs of widespread disease at the time of presentation. In those cases, clonality analysis was not applied for treatment decisions. Therefore, patients with widespread metastatic disease were not included in our study. Patients in our study had only one or a very limited number of tumors, so the degree of metastatic spread at the time of presentation was lower than a random cohort of metastatic lung cancer patients [77,78]. Thus, selection of cases in our study explains the paradox.

Information about the stage of disease was not presented, because the stage is highly dependent upon the outcome of the clonality analysis and is therefore biased. Non-clonal tumors are staged independently, while clonal tumors have impact upon T- or M-status and therefore the final stage of disease [79].

## Conclusion

In summary, genome-wide CNA profiling is a suitable and reliable technique to determine clonality in daily clinical practice. Although, some concordance between histological comparison and CNA profiling was found, arguments exist to use extensive molecular testing, like CNA, to determine whether a second tumor is a metastasis or a second primary.

## Supporting information

S1 Figures All CNA profiles (1A–90B). (PDF)
