# Correction: Clonality analysis of pulmonary tumors by genome-wide copy number profiling

**DOI:** 10.1371/journal.pone.0225733

**Published:** 2019-11-19

**Authors:** Julien P. L. Vincenten, Hendrik F. van Essen, Birgit I. Lissenberg-Witte, Nicole W. J. Bulkmans, Oscar Krijgsman, Daoud Sie, Paul P. Eijk, Egbert F. Smit, Bauke Ylstra, Erik Thunnissen

The first two authors, Julien P. L. Vincenten and Hendrik F. van Essen, should be noted as contributing equally to this work.

The last two authors, Bauke Ylstra and Erik Thunnissen, should be noted as contributing equally to this work as last author.
