# Supplementary material for: Clonality analysis of pulmonary tumors by genome-wide copy number profiling
Source: PLoS One. 2019 Oct 16;14(10):e0223827. doi: 10.1371/journal.pone.0223827 (PMC6795528; doi:10.1371/journal.pone.0223827)
Supplement: S1 Figures — (PDF) [file pone.0223827.s001.pdf]

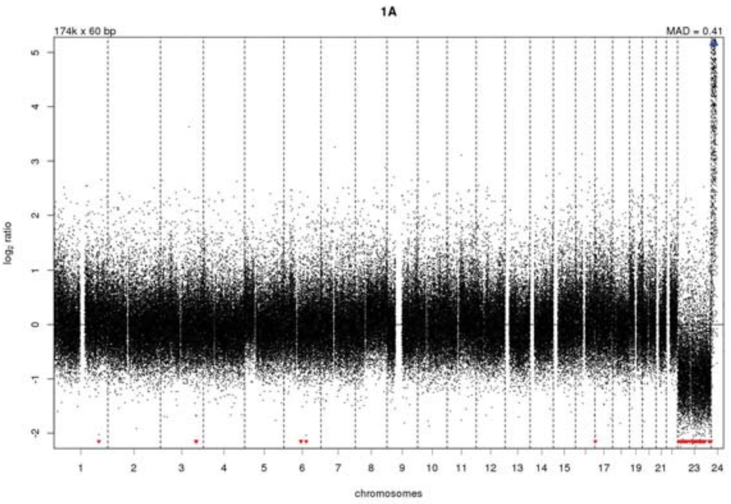
1A
174k x 60 bp
MAD = 0.41
log2 ratio
chromosomes

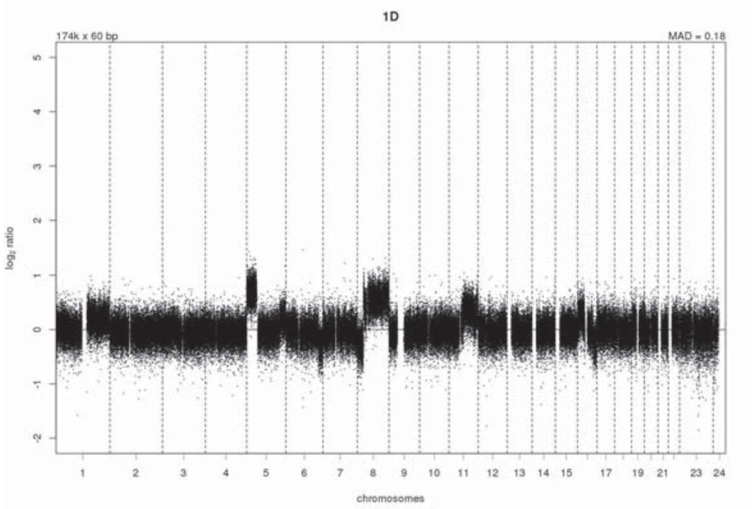
1D
174k x 60 bp
MAD = 0.18
log2 ratio
chromosomes

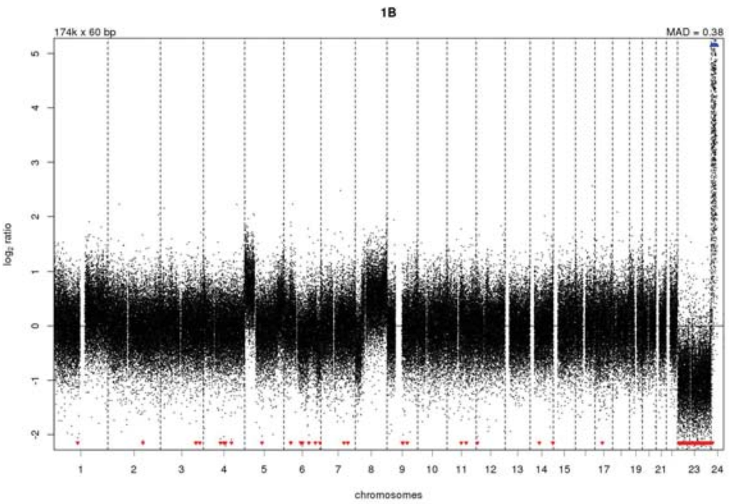
1B
174k x 60 bp
MAD = 0.38
log2 ratio
chromosomes

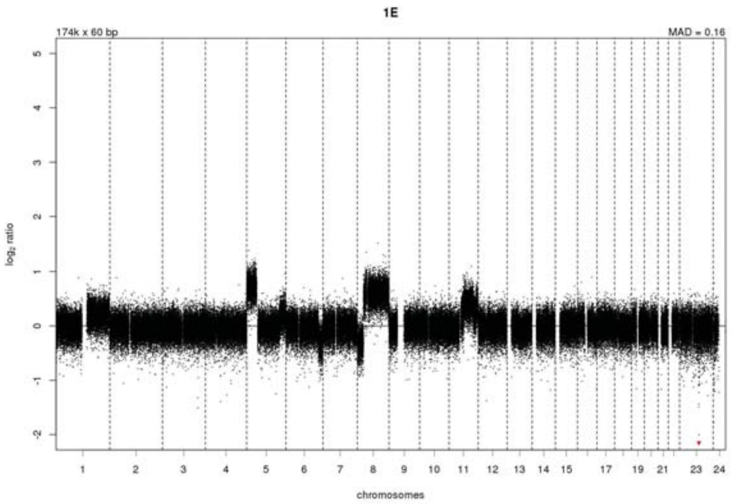
1E
174k x 60 bp
MAD = 0.16
log2 ratio
chromosomes

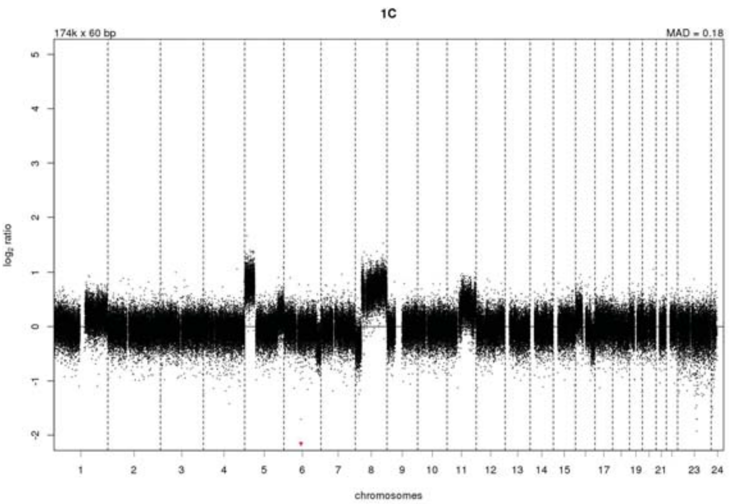
1C
174k x 60 bp
MAD = 0.18
log2 ratio
chromosomes

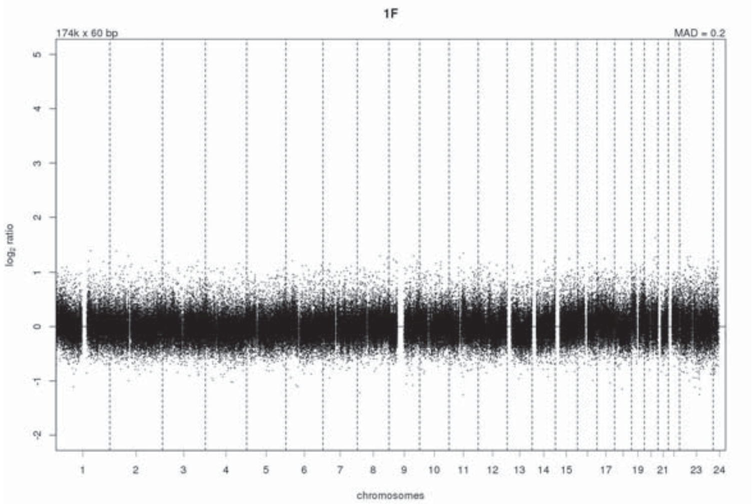
1F
174k x 60 bp
MAD = 0.2
log2 ratio
chromosomes

2A

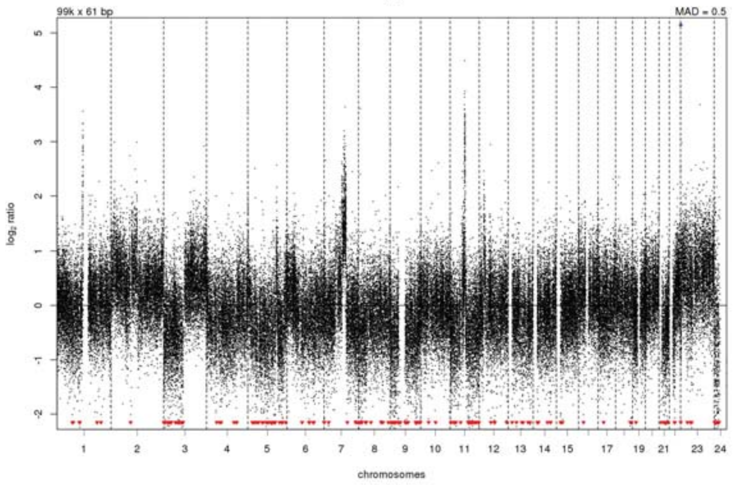
99k x 61 bp
MAD = 0.5
log2 ratio
chromosomes
1
2
3
4
5
6
7
8
9
10
11
12
13
14
15
17
19
21
23
24

2B

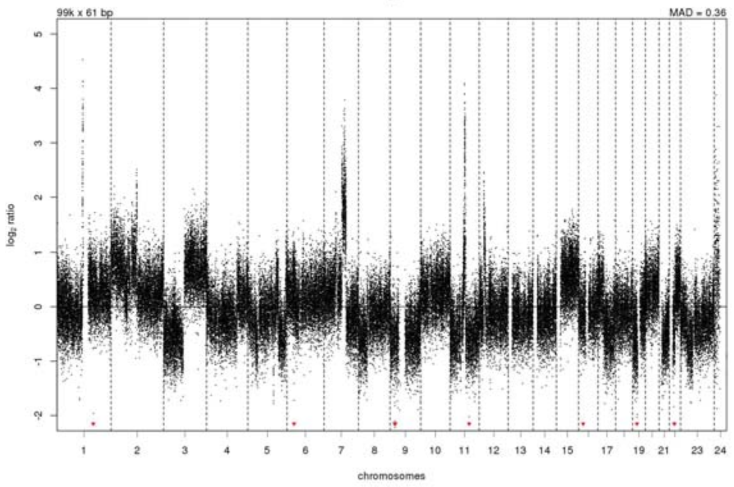
99k x 61 bp
MAD = 0.36
log2 ratio
chromosomes
1
2
3
4
5
6
7
8
9
10
11
12
13
14
15
17
19
21
23
24

3A

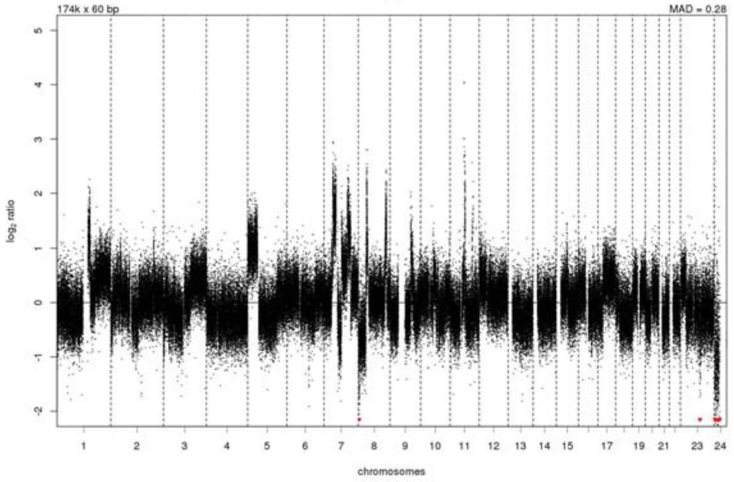
174k x 60 bp
MAD = 0.28
log2 ratio
5
4
3
2
1
0
-1
-2
1
2
3
4
5
6
7
8
9
10
11
12
13
14
15
17
19
21
23
24
chromosomes

3B

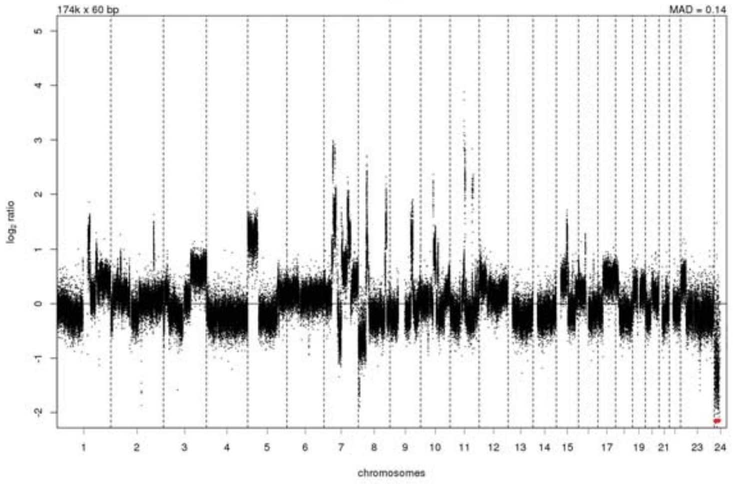
174k x 60 bp
MAD = 0.14
log2 ratio
5
4
3
2
1
0
-1
-2
1
2
3
4
5
6
7
8
9
10
11
12
13
14
15
17
19
21
23
24
chromosomes

4A

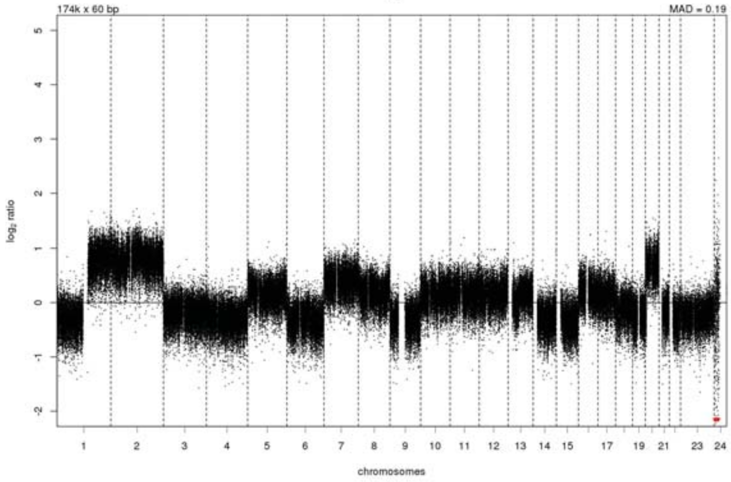

174k x 60 bp
MAD = 0.19
log2 ratio
chromosomes
1 2 3 4 5 6 7 8 9 10 11 12 13 14 15 17 19 21 23 24

4B

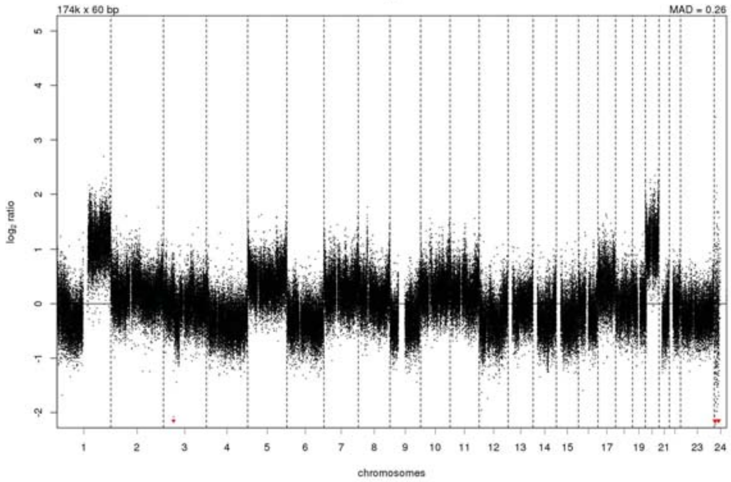

174k x 60 bp
MAD = 0.26
log2 ratio
chromosomes
1 2 3 4 5 6 7 8 9 10 11 12 13 14 15 17 19 21 23 24

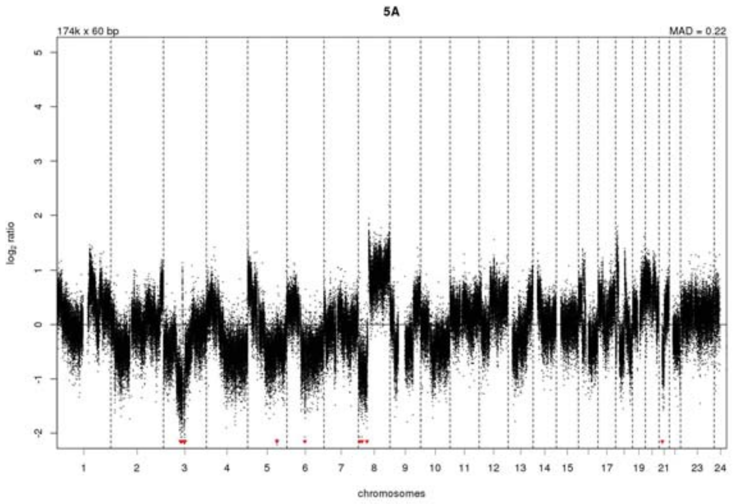

5A
174k x 60 bp
MAD = 0.22
log2 ratio
chromosomes

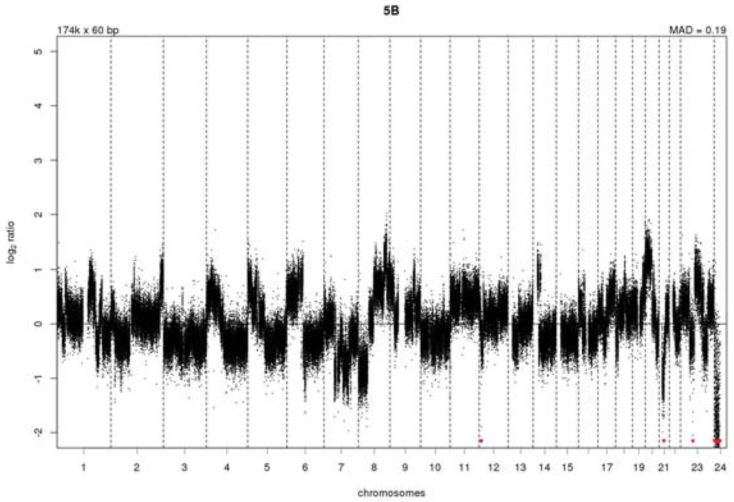

5B
174k x 60 bp
MAD = 0.19
log2 ratio
chromosomes

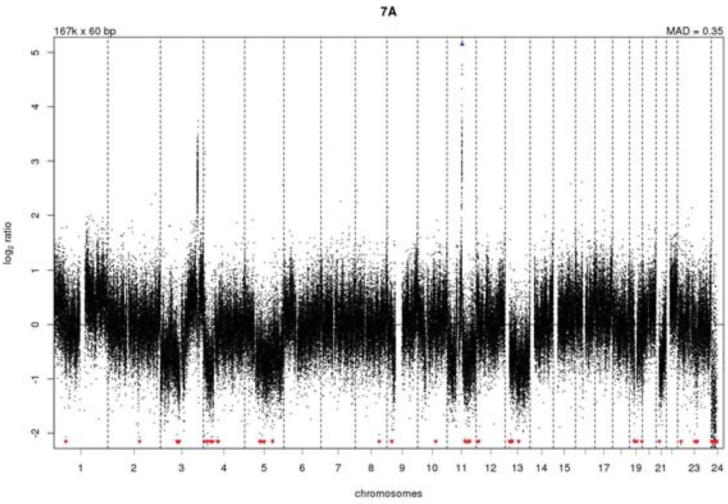
7A
167k x 60 bp
MAD = 0.35
log2 ratio
chromosomes

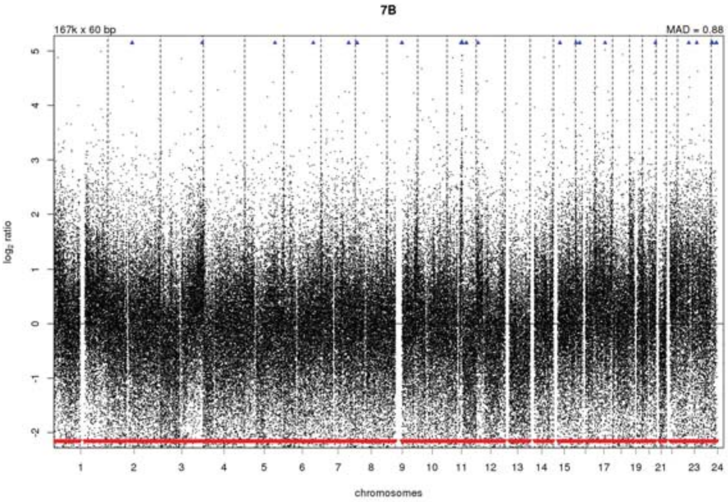
7B
167k x 60 bp
MAD = 0.88
log2 ratio
chromosomes

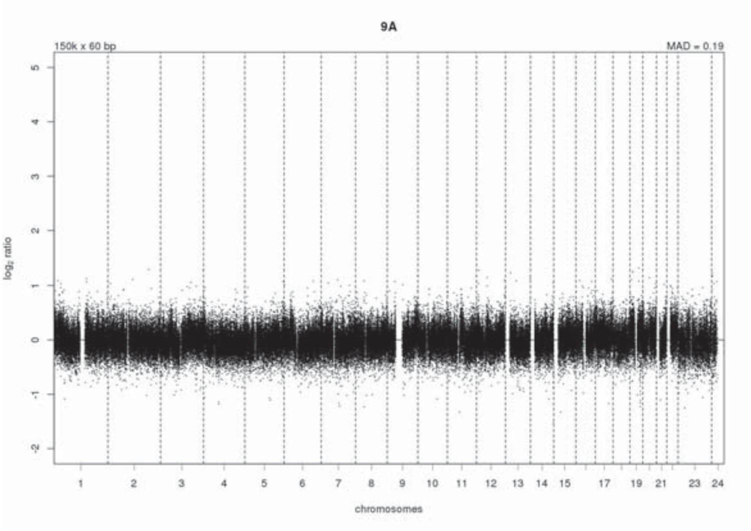
9A
150k x 60 bp
MAD = 0.19
log2 ratio
chromosomes

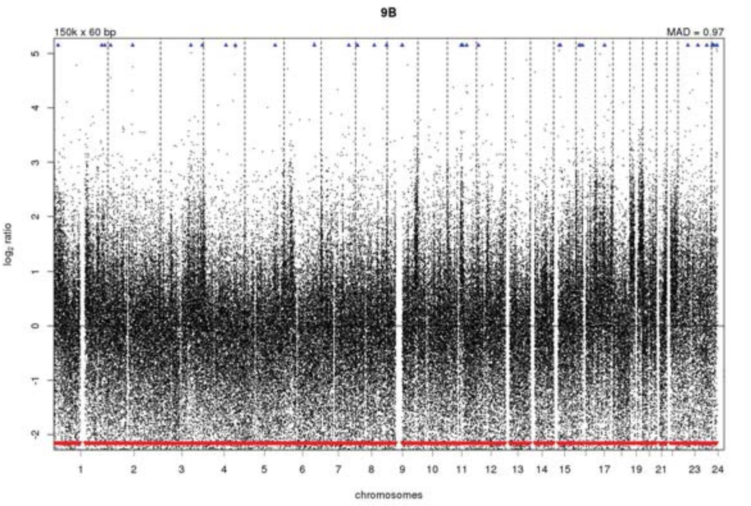
9B
150k x 60 bp
MAD = 0.97
log2 ratio
chromosomes

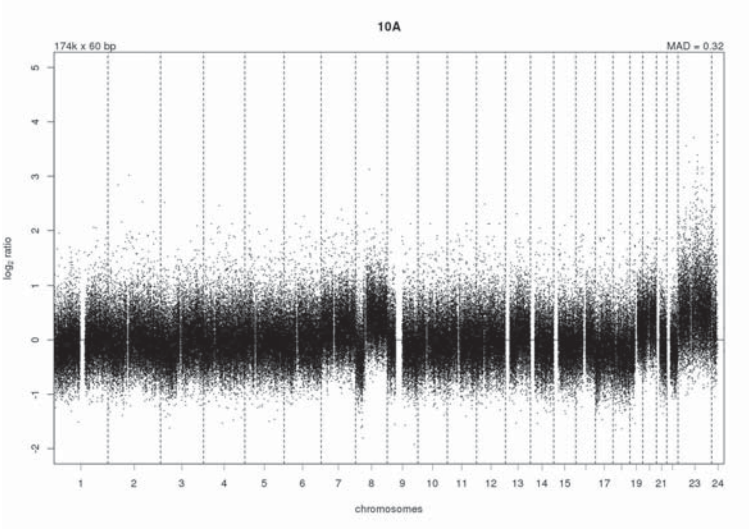
10A
174k x 60 bp
MAD = 0.32
log2 ratio
chromosomes
1 2 3 4 5 6 7 8 9 10 11 12 13 14 15 17 19 21 23 24

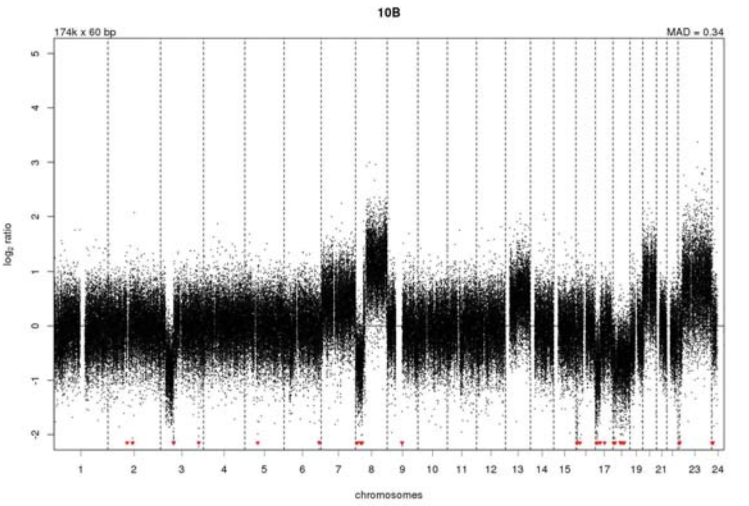
10B
174k x 60 bp
MAD = 0.34
log2 ratio
chromosomes
1 2 3 4 5 6 7 8 9 10 11 12 13 14 15 17 19 21 23 24

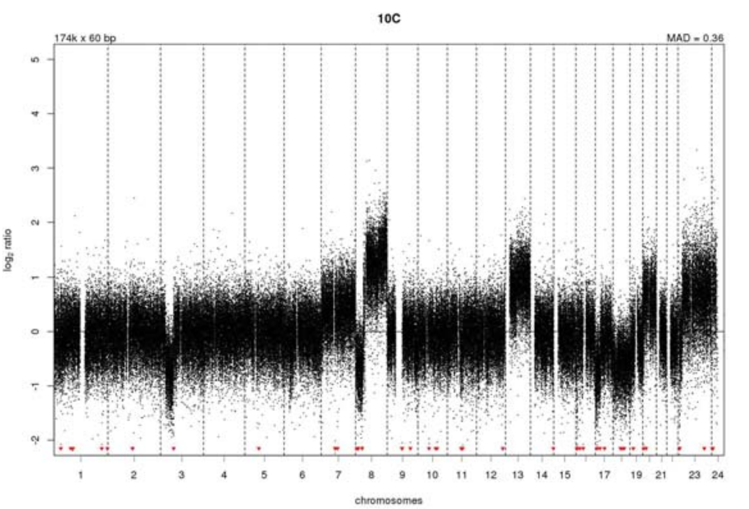
10C
174k x 60 bp
MAD = 0.36
log2 ratio
chromosomes
1 2 3 4 5 6 7 8 9 10 11 12 13 14 15 17 19 21 23 24

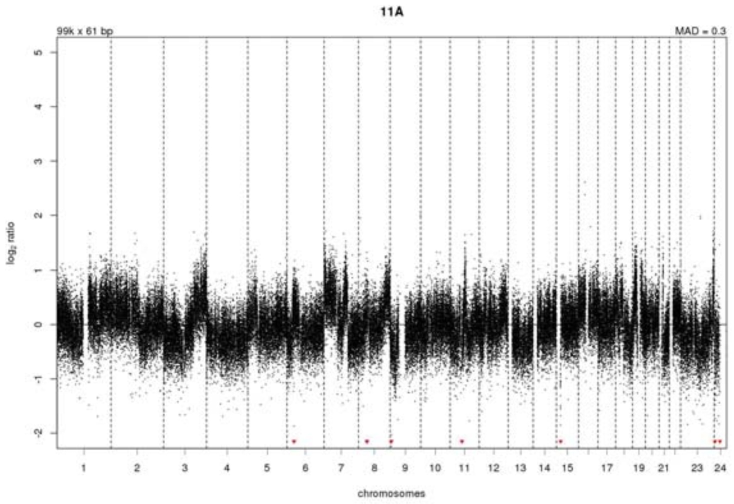
11A
99k x 61 bp
MAD = 0.3
log2 ratio
chromosomes

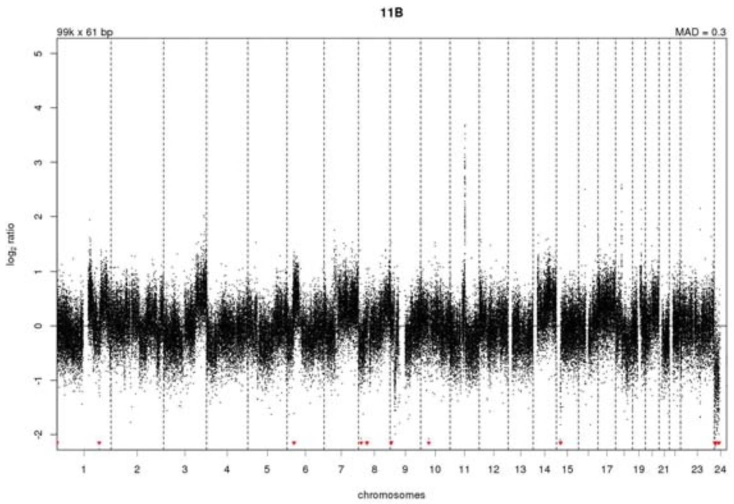
11B
99k x 61 bp
MAD = 0.3
log2 ratio
chromosomes

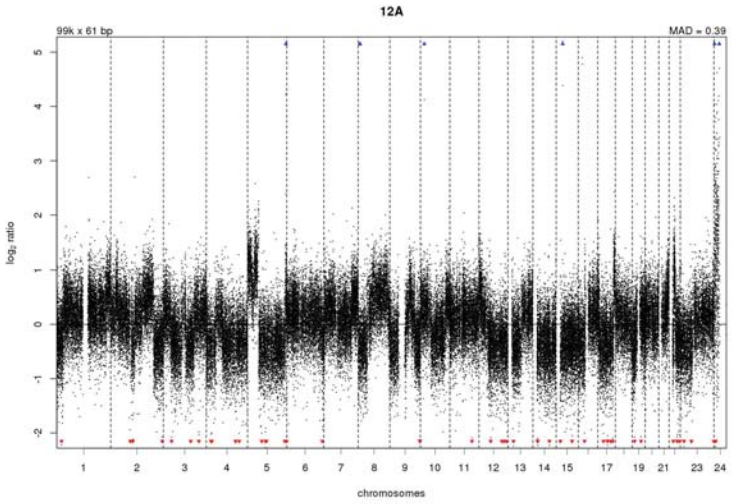

12A
99k x 61 bp
MAD = 0.39
log2 ratio
chromosomes

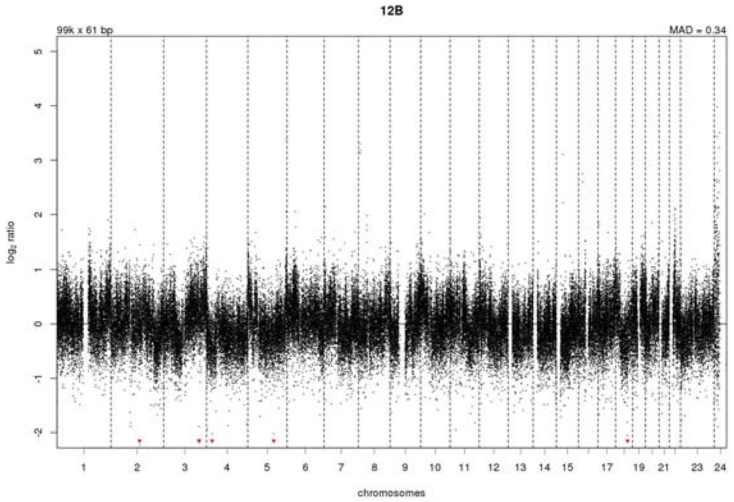

12B
99k x 61 bp
MAD = 0.34
log2 ratio
chromosomes

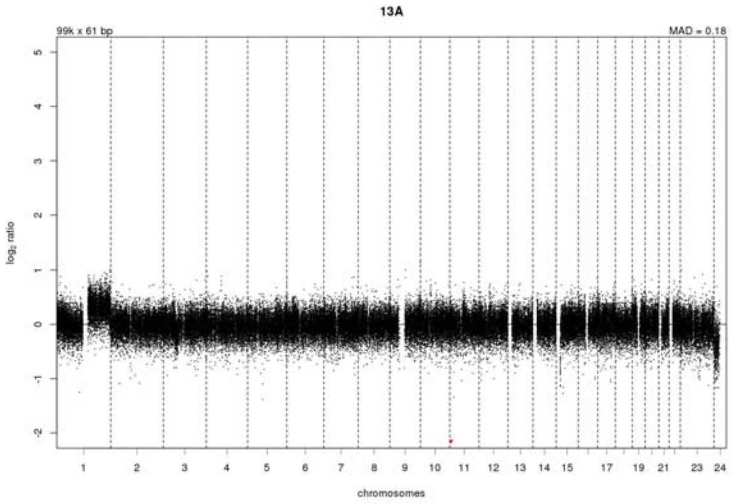
13A
99k x 61 bp
MAD = 0.18
log2 ratio
chromosomes
1
2
3
4
5
6
7
8
9
10
11
12
13
14
15
17
19
21
23
24

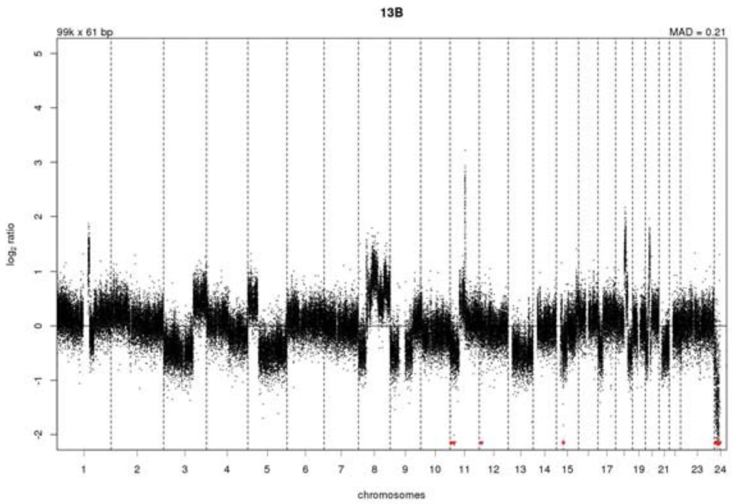
13B
99k x 61 bp
MAD = 0.21
log2 ratio
chromosomes
1
2
3
4
5
6
7
8
9
10
11
12
13
14
15
17
19
21
23
24

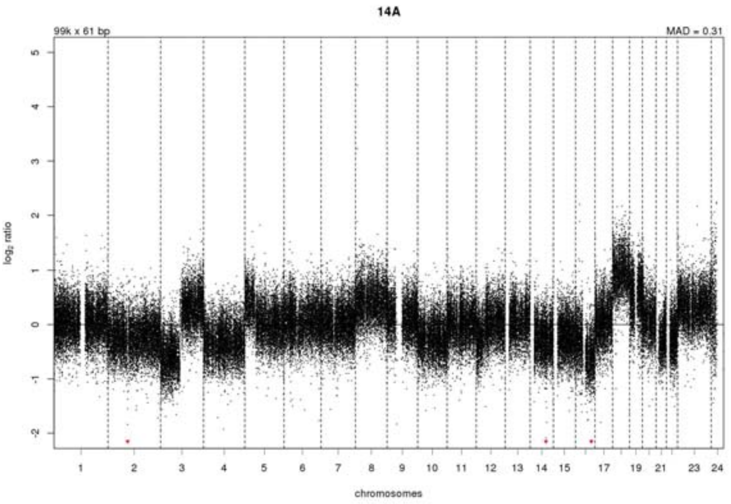
14A
99k x 61 bp
MAD = 0.31
log2 ratio
chromosomes
1 2 3 4 5 6 7 8 9 10 11 12 13 14 15 17 19 21 23 24

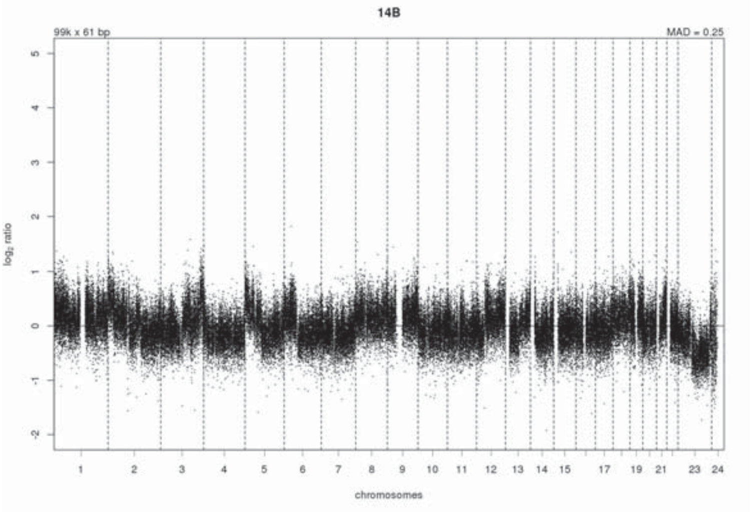
14B
99k x 61 bp
MAD = 0.25
log2 ratio
chromosomes
1 2 3 4 5 6 7 8 9 10 11 12 13 14 15 17 19 21 23 24

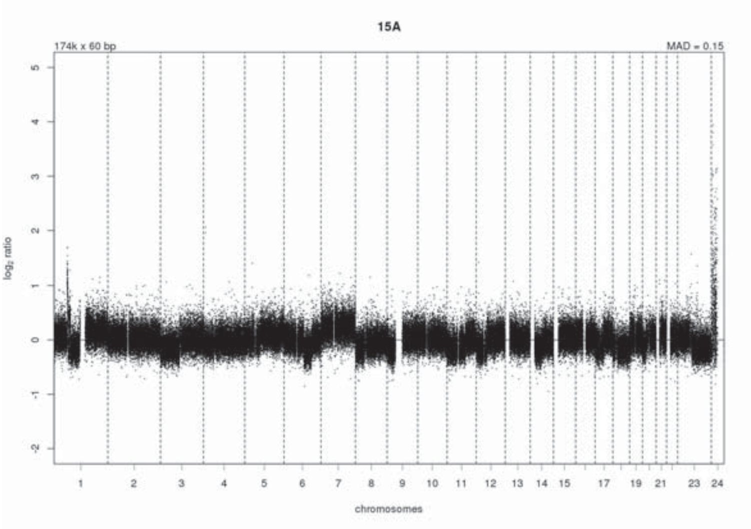
15A
174k x 60 bp
MAD = 0.15
log2 ratio
chromosomes

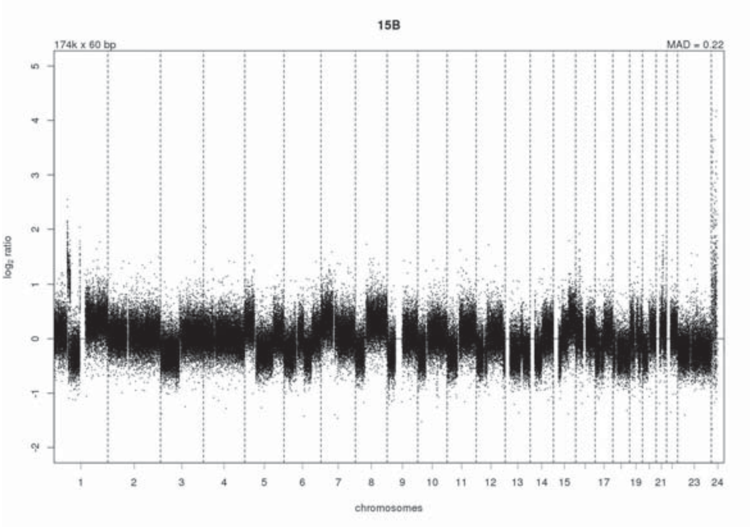
15B
174k x 60 bp
MAD = 0.22
log2 ratio
chromosomes

16A

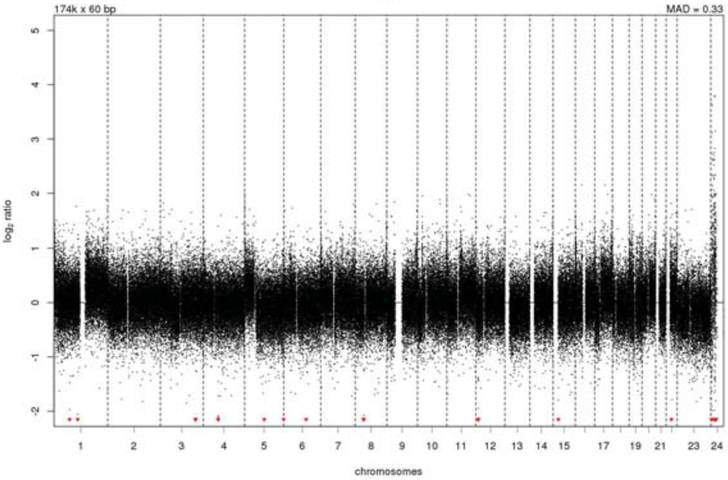
174k x 60 bp
MAD = 0.33
log2 ratio
chromosomes
1 2 3 4 5 6 7 8 9 10 11 12 13 14 15 17 19 21 23 24

16B

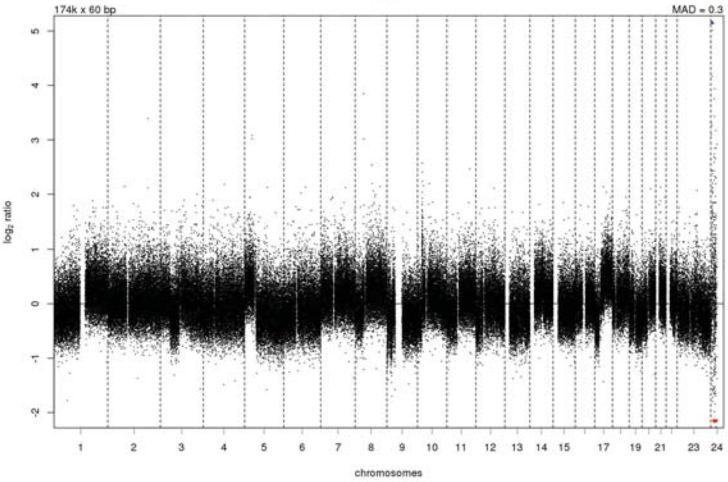
174k x 60 bp
MAD = 0.3
log2 ratio
chromosomes
1 2 3 4 5 6 7 8 9 10 11 12 13 14 15 17 19 21 23 24

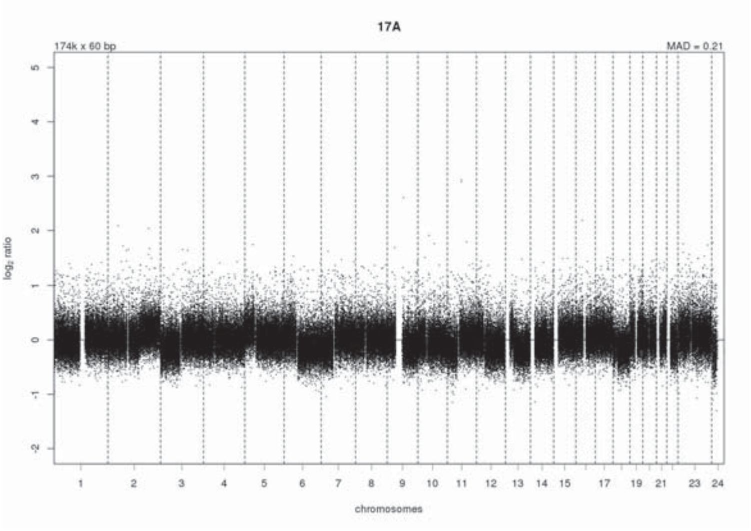

17A
174k x 60 bp
MAD = 0.21
log2 ratio
chromosomes

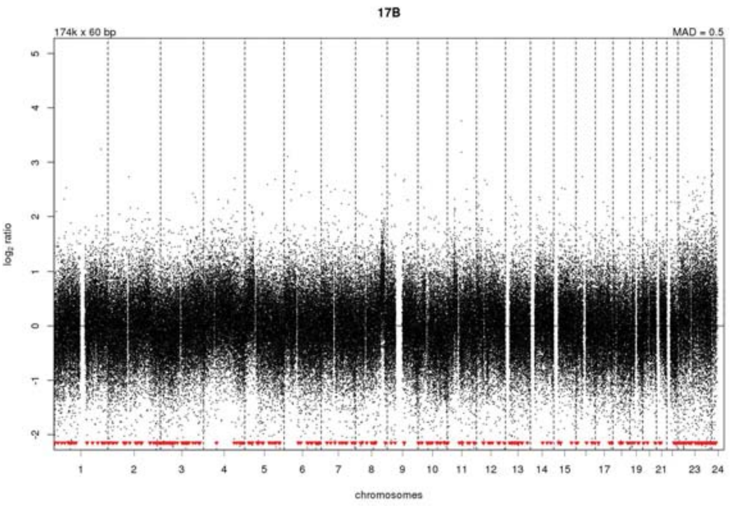

17B
174k x 60 bp
MAD = 0.5
log2 ratio
chromosomes

18A

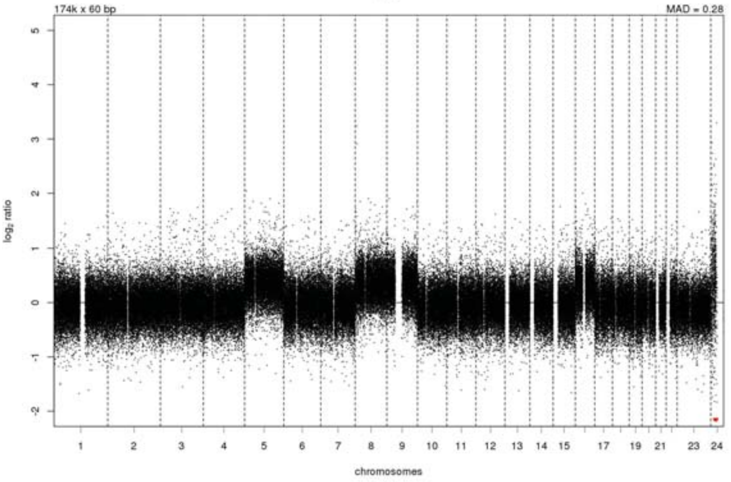
174k x 60 bp
MAD = 0.28
log2 ratio
5 4 3 2 1 0 -1 -2
1 2 3 4 5 6 7 8 9 10 11 12 13 14 15 17 19 21 23 24
chromosomes

18B

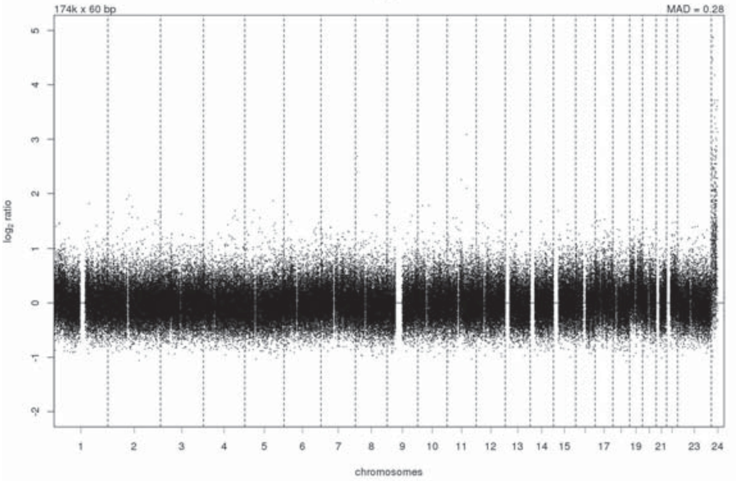
174k x 60 bp
MAD = 0.28
log2 ratio
5 4 3 2 1 0 -1 -2
1 2 3 4 5 6 7 8 9 10 11 12 13 14 15 17 19 21 23 24
chromosomes

18C

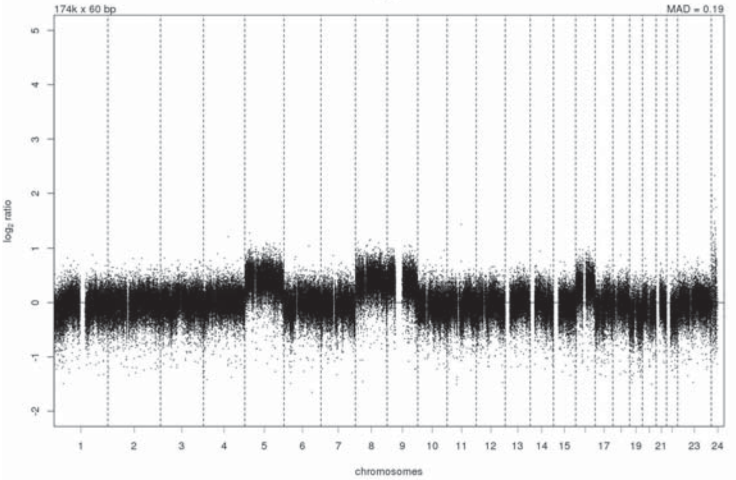
174k x 60 bp
MAD = 0.19
log2 ratio
5 4 3 2 1 0 -1 -2
1 2 3 4 5 6 7 8 9 10 11 12 13 14 15 17 19 21 23 24
chromosomes

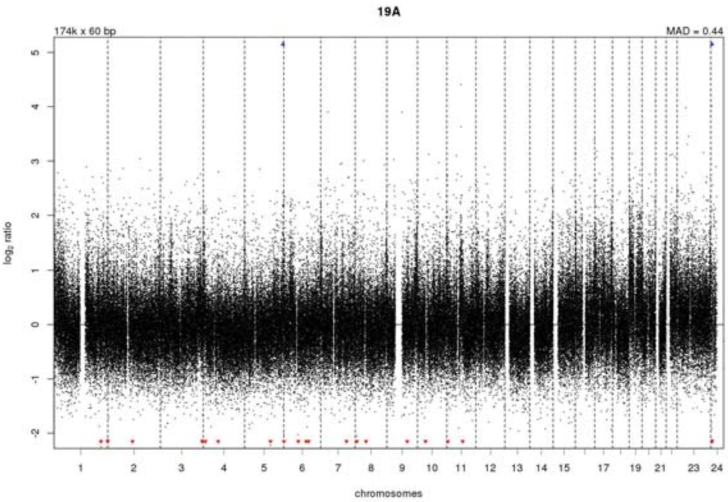
19A
174k x 60 bp
MAD = 0.44
log2 ratio
chromosomes

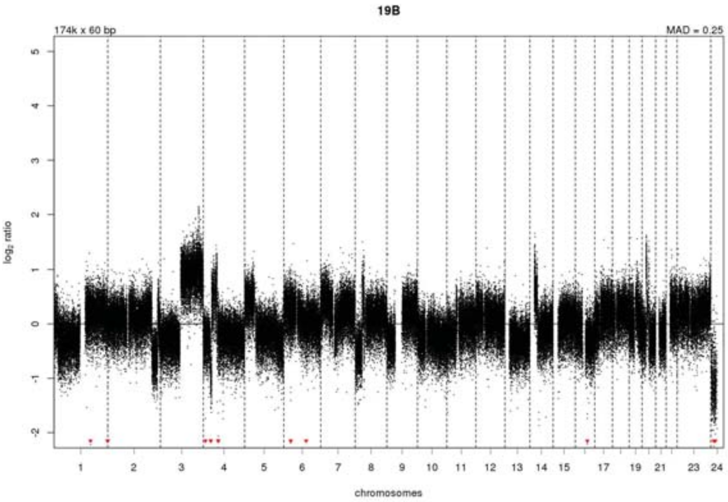
19B
174k x 60 bp
MAD = 0.25
log2 ratio
chromosomes

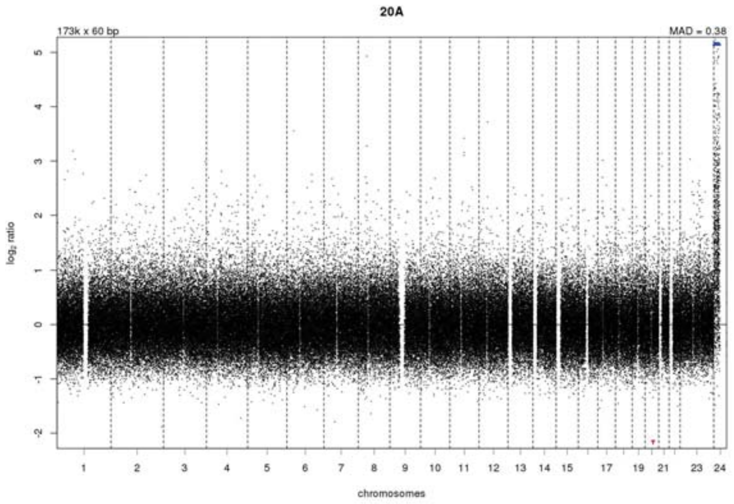
20A
173k x 60 bp
MAD = 0.38
log2 ratio
chromosomes

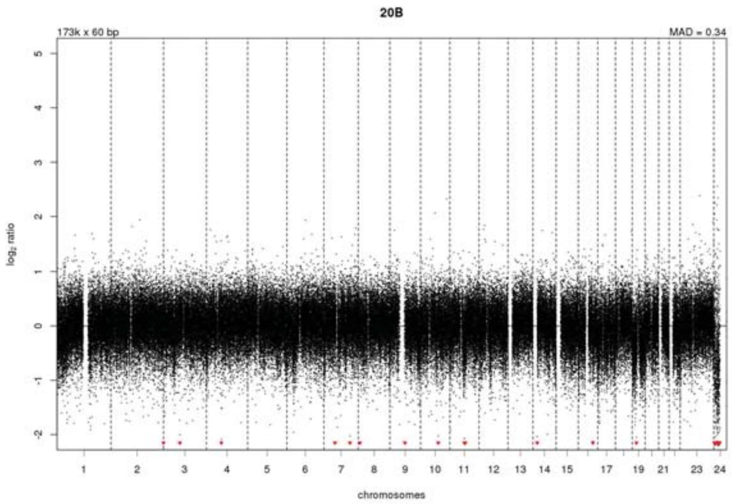
20B
173k x 60 bp
MAD = 0.34
log2 ratio
chromosomes

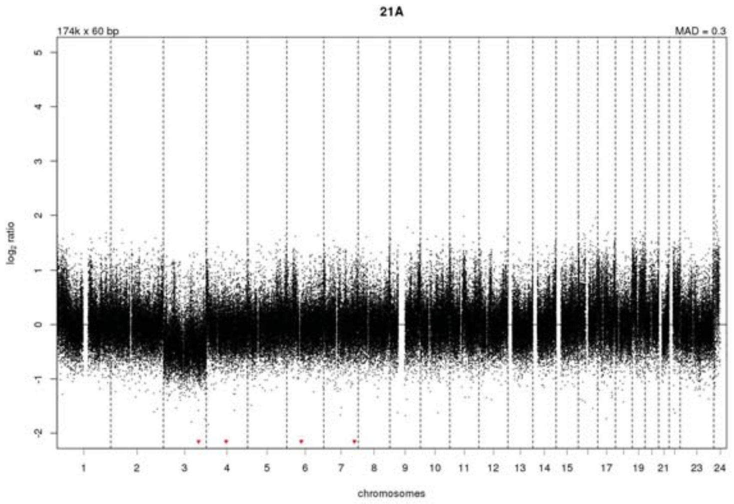
21A
174k x 60 bp
MAD = 0.3
log2 ratio
chromosomes

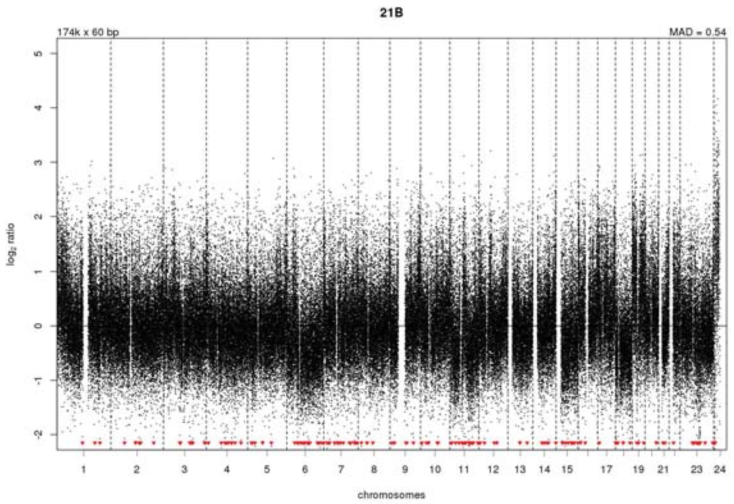
21B
174k x 60 bp
MAD = 0.54
log2 ratio
chromosomes

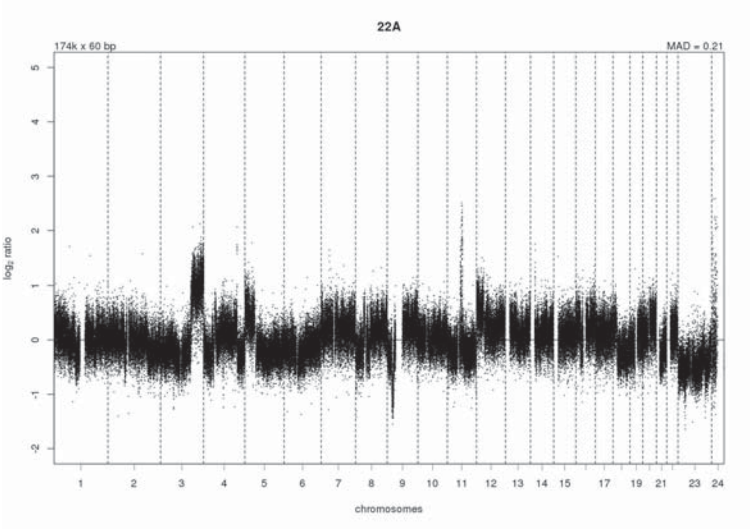
22A
174k x 60 bp
MAD = 0.21
log2 ratio
chromosomes

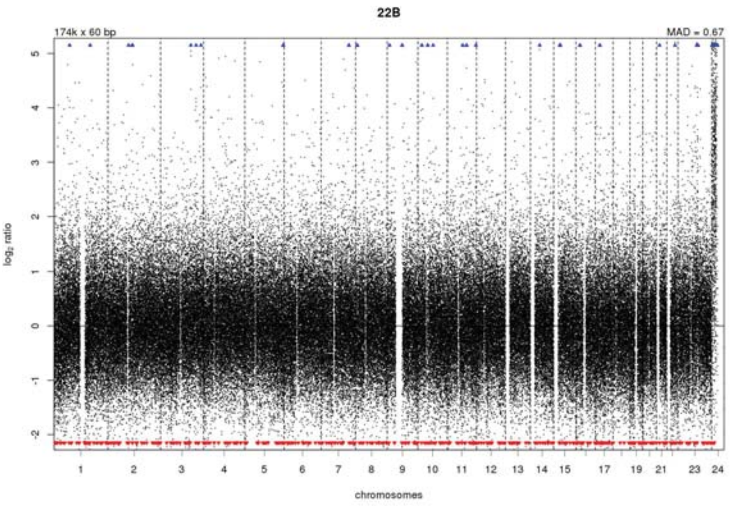
22B
174k x 60 bp
MAD = 0.67
log2 ratio
chromosomes

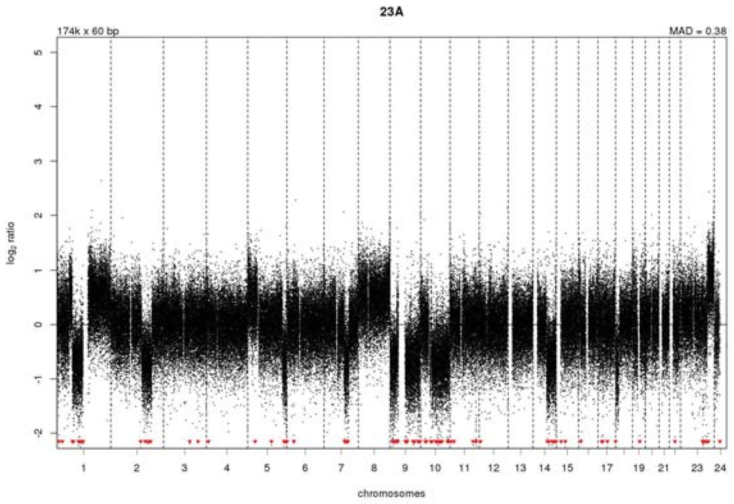

23A
174k x 60 bp
MAD = 0.38
log2 ratio
chromosomes

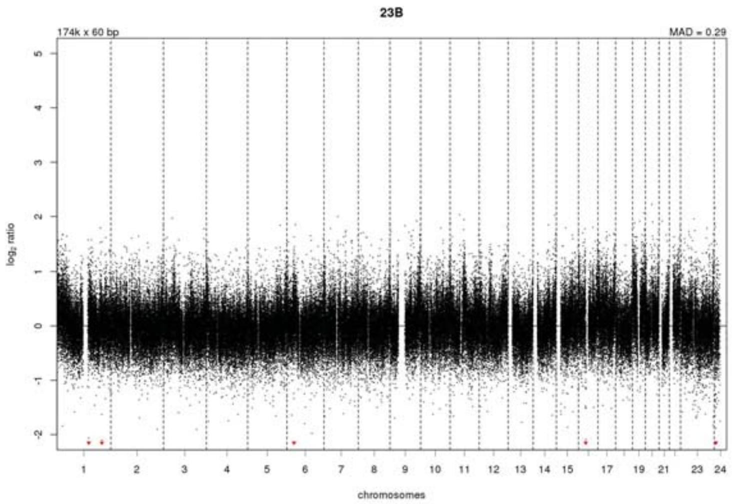

23B
174k x 60 bp
MAD = 0.29
log2 ratio
chromosomes

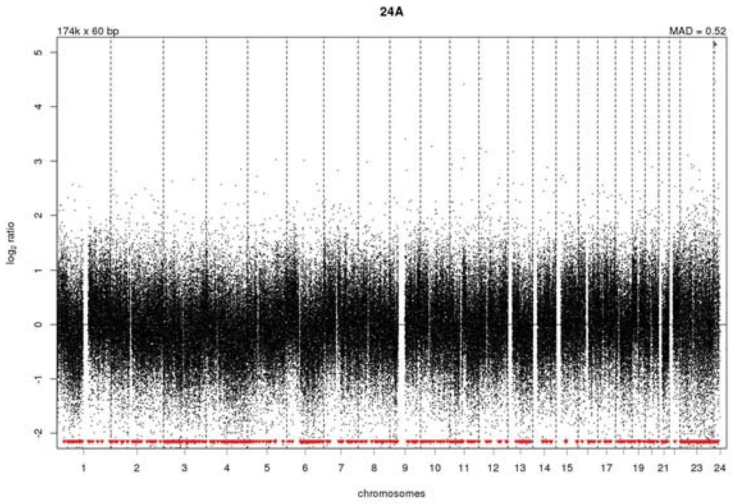

24A
174k x 60 bp
MAD = 0.52
log2 ratio
chromosomes
1 2 3 4 5 6 7 8 9 10 11 12 13 14 15 17 19 21 23 24

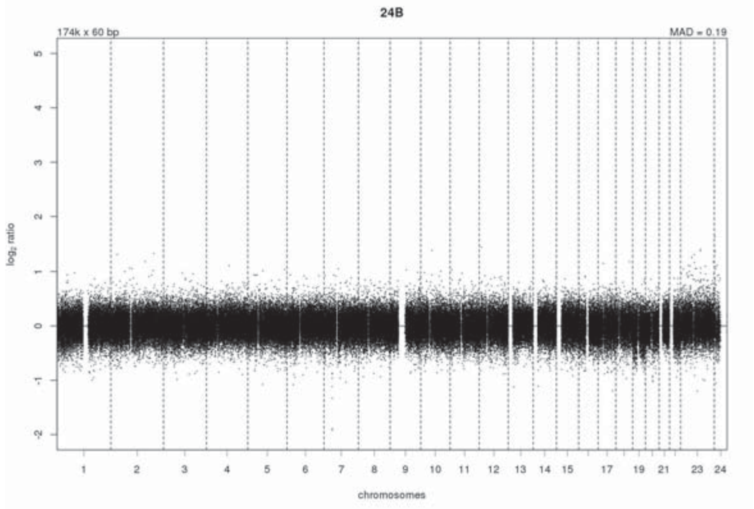

24B
174k x 60 bp
MAD = 0.19
log2 ratio
chromosomes
1 2 3 4 5 6 7 8 9 10 11 12 13 14 15 17 19 21 23 24

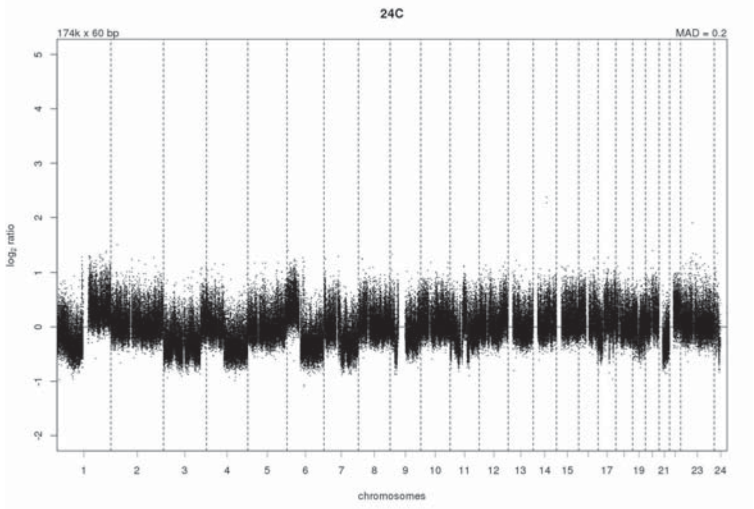

24C
174k x 60 bp
MAD = 0.2
log2 ratio
chromosomes
1 2 3 4 5 6 7 8 9 10 11 12 13 14 15 17 19 21 23 24

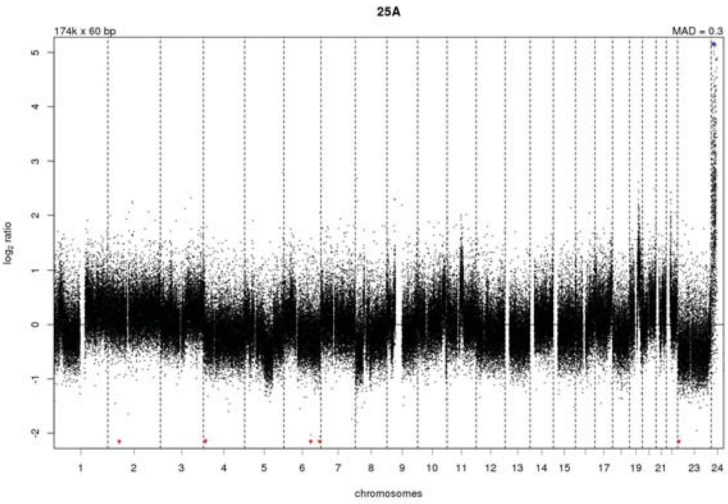
25A
174k x 60 bp
MAD = 0.3
log2 ratio
chromosomes

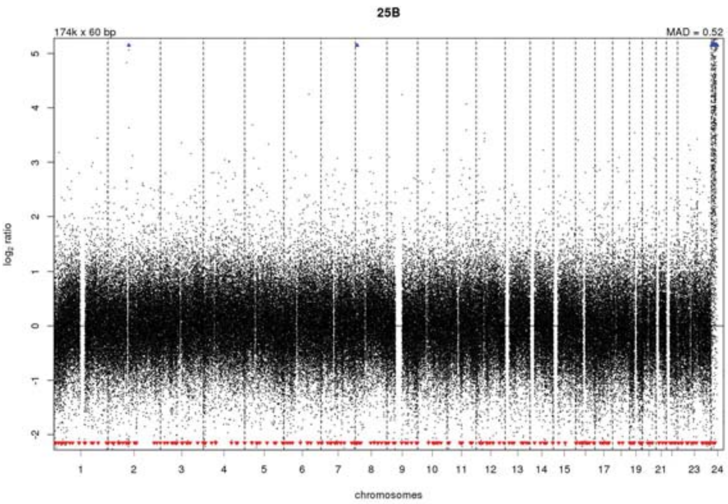
25B
174k x 60 bp
MAD = 0.52
log2 ratio
chromosomes

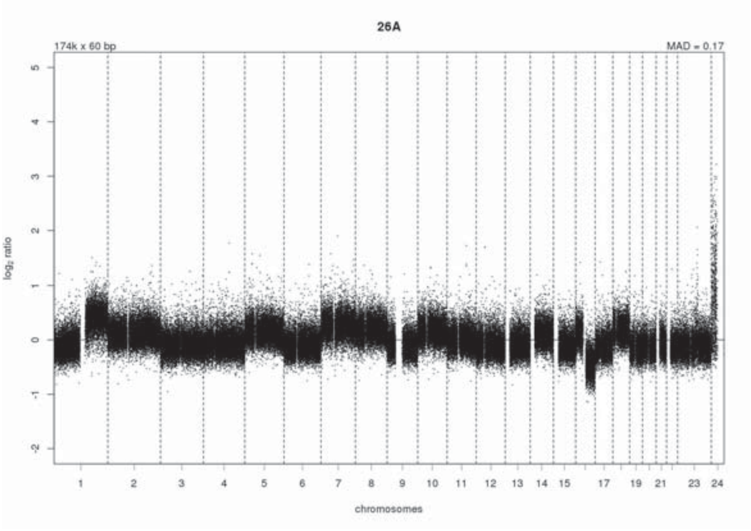
26A
174k x 60 bp
MAD = 0.17
log2 ratio
chromosomes
1 2 3 4 5 6 7 8 9 10 11 12 13 14 15 17 19 21 23 24

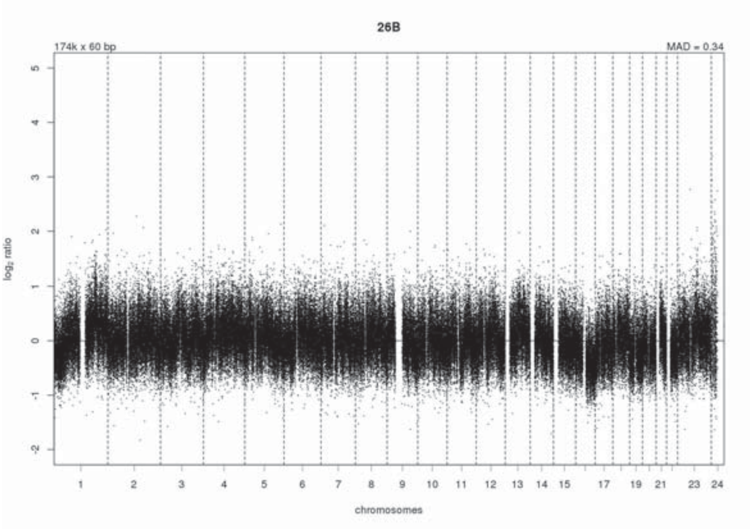
26B
174k x 60 bp
MAD = 0.34
log2 ratio
chromosomes
1 2 3 4 5 6 7 8 9 10 11 12 13 14 15 17 19 21 23 24

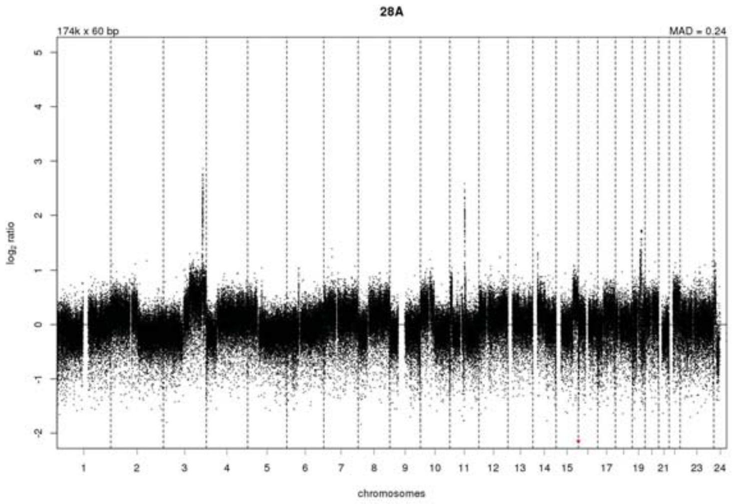
28A
174k x 60 bp
MAD = 0.24
log2 ratio
5
4
3
2
1
0
-1
-2
1 2 3 4 5 6 7 8 9 10 11 12 13 14 15 17 19 21 23 24
chromosomes

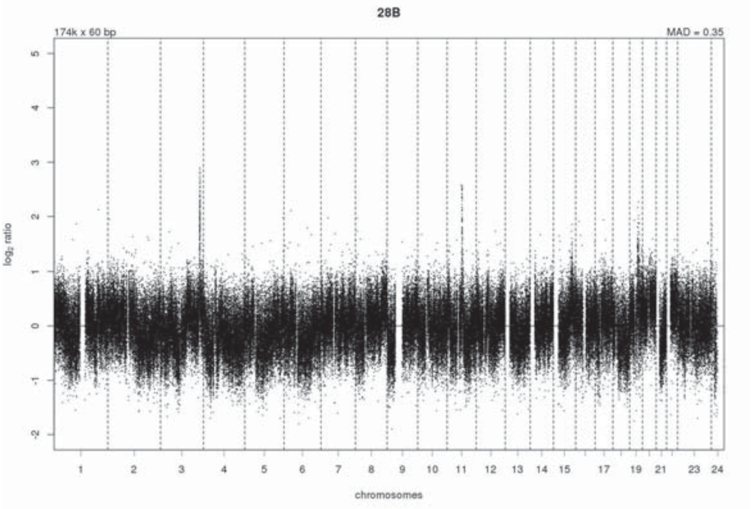
28B
174k x 60 bp
MAD = 0.35
log2 ratio
5
4
3
2
1
0
-1
-2
1 2 3 4 5 6 7 8 9 10 11 12 13 14 15 17 19 21 23 24
chromosomes

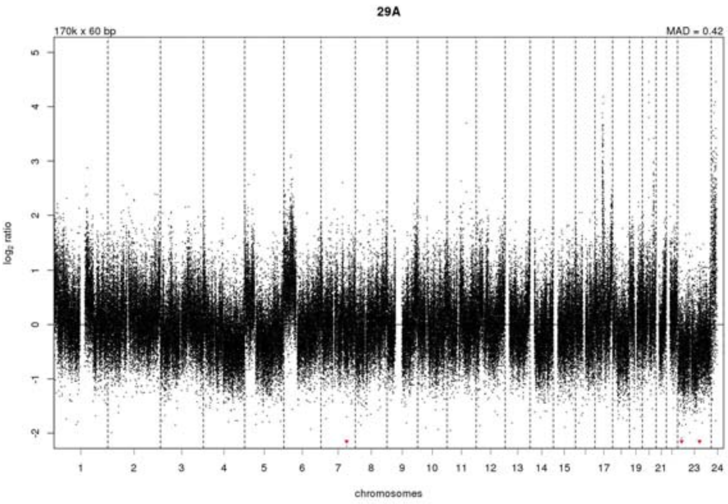
29A
170k x 60 bp
MAD = 0.42
log2 ratio
chromosomes

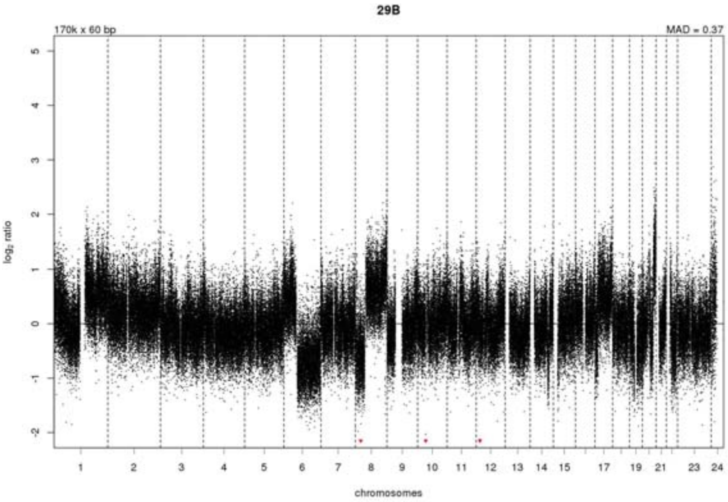
29B
170k x 60 bp
MAD = 0.37
log2 ratio
chromosomes

30A

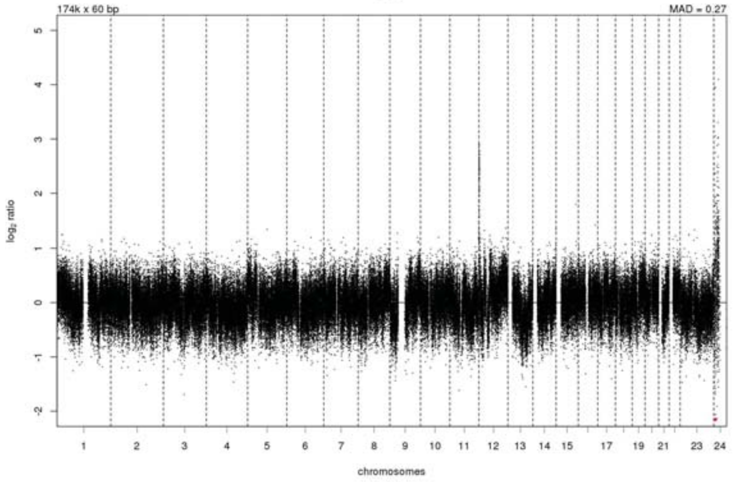
174k x 60 bp
MAD = 0.27
log2 ratio
-2 -1 0 1 2 3 4 5
1 2 3 4 5 6 7 8 9 10 11 12 13 14 15 17 19 21 23 24
chromosomes

30B

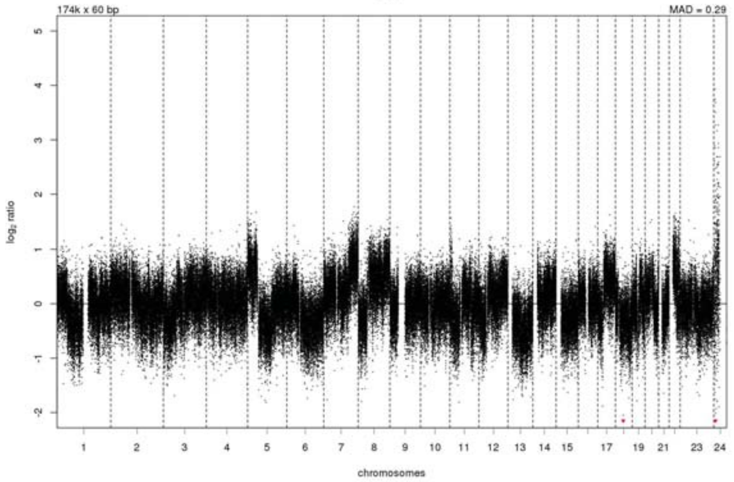
174k x 60 bp
MAD = 0.29
log2 ratio
-2 -1 0 1 2 3 4 5
1 2 3 4 5 6 7 8 9 10 11 12 13 14 15 17 19 21 23 24
chromosomes

30C

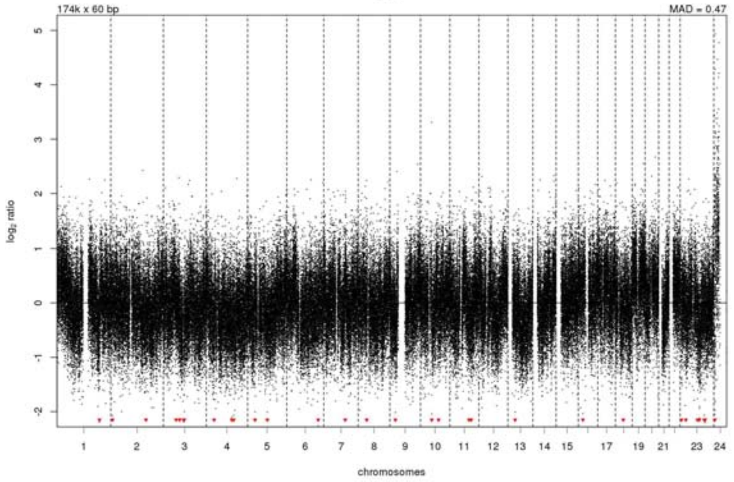
174k x 60 bp
MAD = 0.47
log2 ratio
-2 -1 0 1 2 3 4 5
1 2 3 4 5 6 7 8 9 10 11 12 13 14 15 17 19 21 23 24
chromosomes

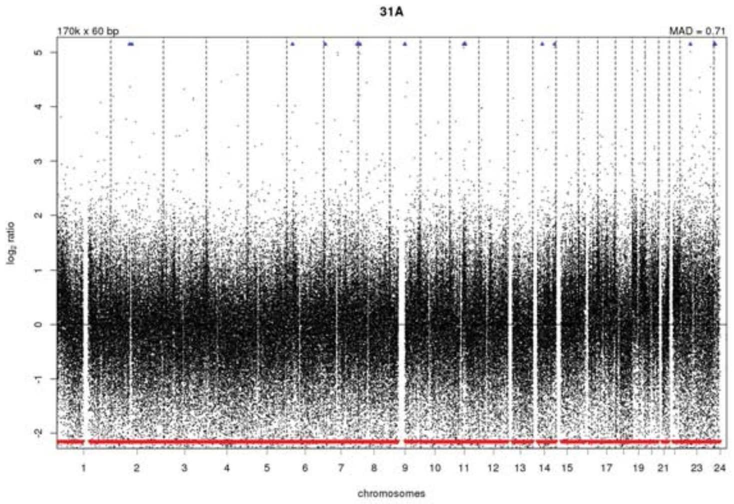
31A
170k x 60 bp
MAD = 0.71
log2 ratio
chromosomes

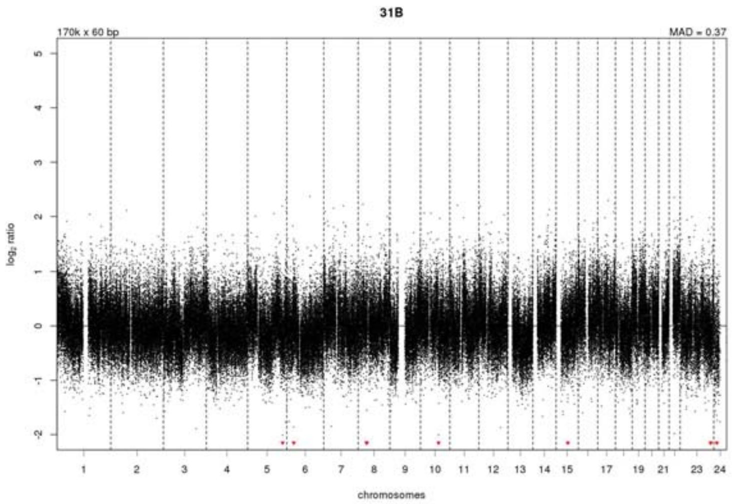
31B
170k x 60 bp
MAD = 0.37
log2 ratio
chromosomes

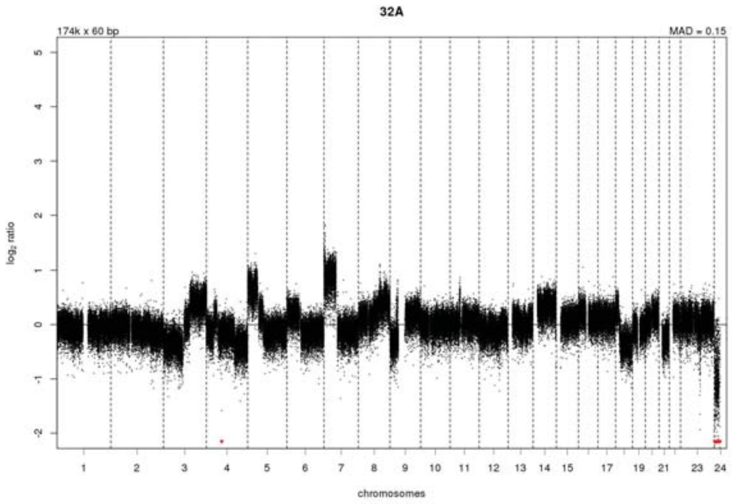
32A
174k x 60 bp
MAD = 0.15
log2 ratio
chromosomes
1
2
3
4
5
6
7
8
9
10
11
12
13
14
15
17
19
21
23
24

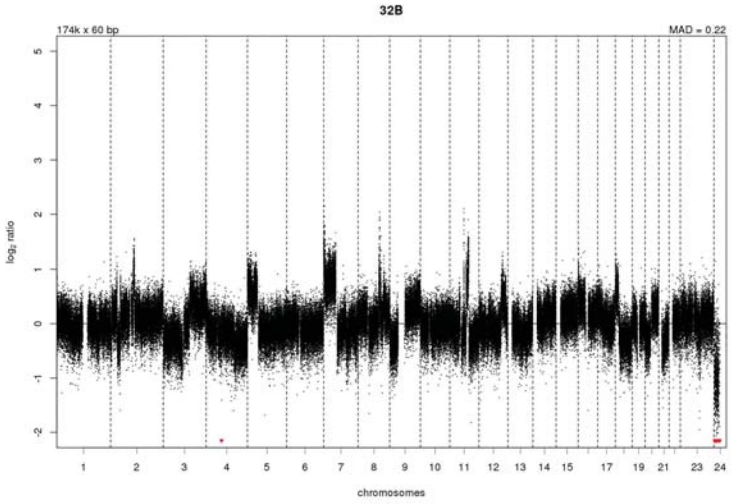
32B
174k x 60 bp
MAD = 0.22
log2 ratio
chromosomes
1
2
3
4
5
6
7
8
9
10
11
12
13
14
15
17
19
21
23
24

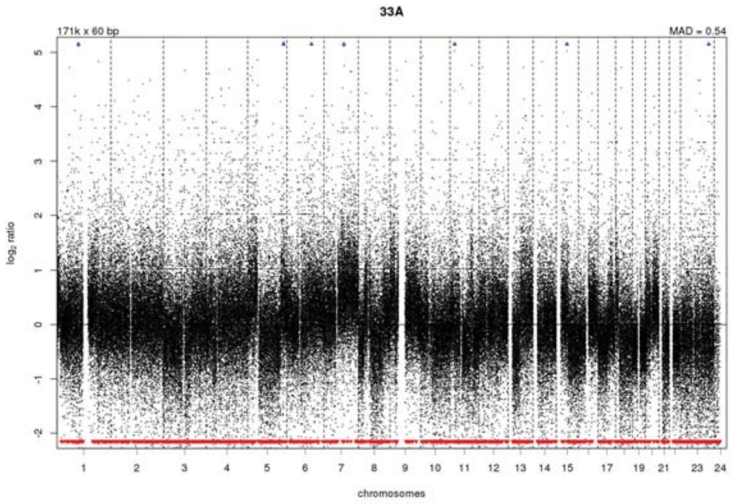
33A
171k x 60 bp
MAD = 0.54
log2 ratio
chromosomes

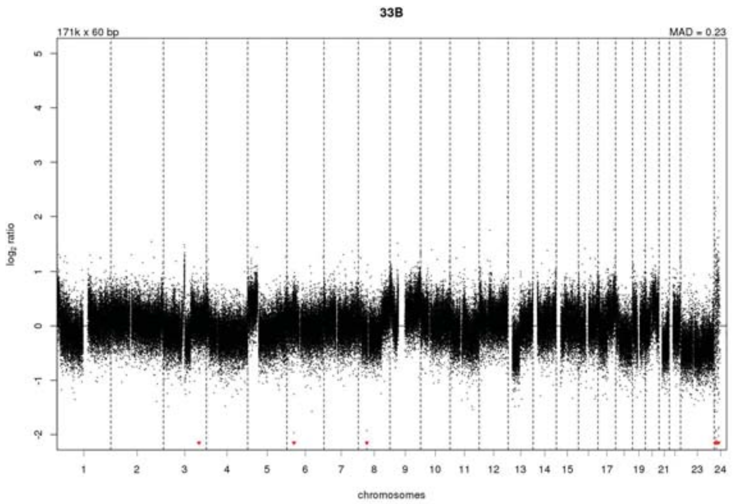
33B
171k x 60 bp
MAD = 0.23
log2 ratio
chromosomes

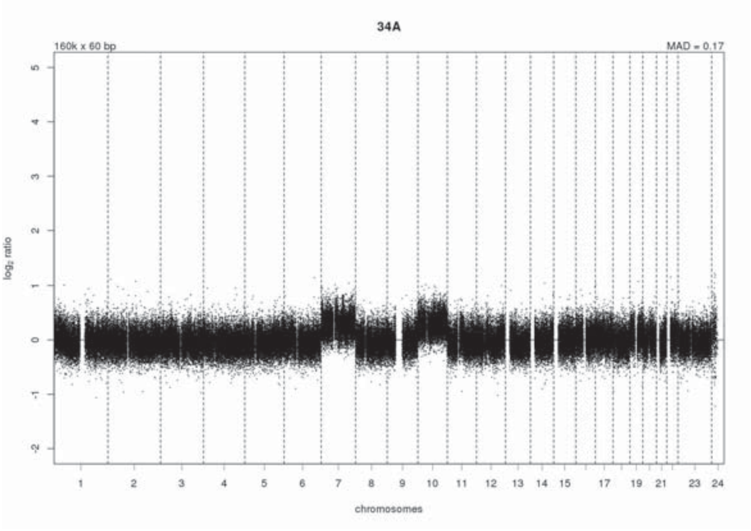
34A
160k x 60 bp
MAD = 0.17
log2 ratio
chromosomes
1
2
3
4
5
6
7
8
9
10
11
12
13
14
15
17
19
21
23
24

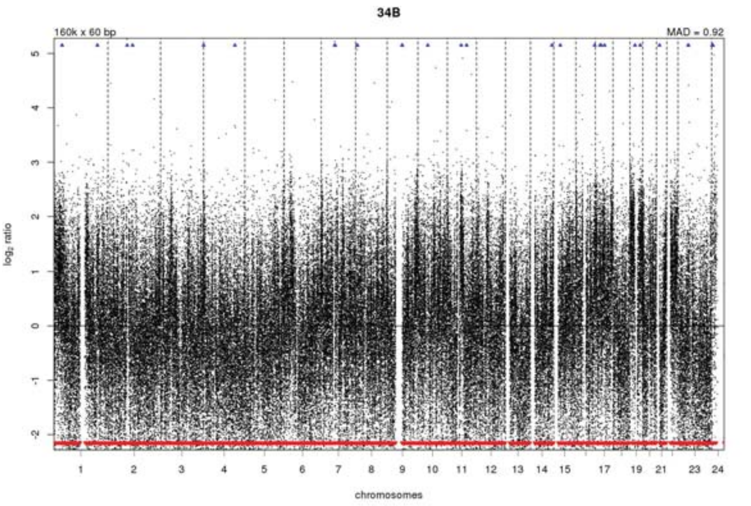
34B
160k x 60 bp
MAD = 0.92
log2 ratio
chromosomes
1
2
3
4
5
6
7
8
9
10
11
12
13
14
15
17
19
21
23
24

35A

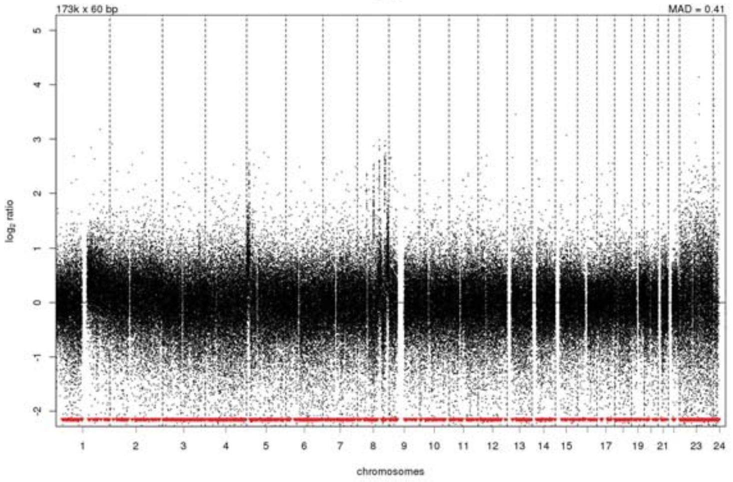
173k x 60 bp
MAD = 0.41
log2 ratio
5
4
3
2
1
0
-1
-2
1
2
3
4
5
6
7
8
9
10
11
12
13
14
15
17
19
21
23
24
chromosomes

35B

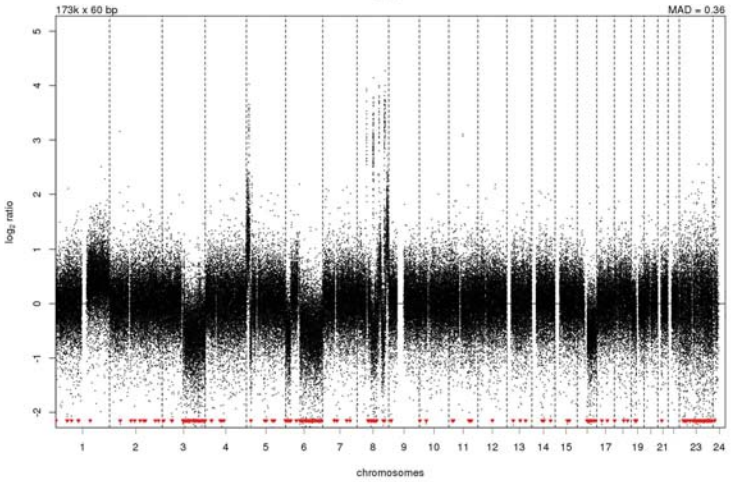
173k x 60 bp
MAD = 0.36
log2 ratio
5
4
3
2
1
0
-1
-2
1
2
3
4
5
6
7
8
9
10
11
12
13
14
15
17
19
21
23
24
chromosomes

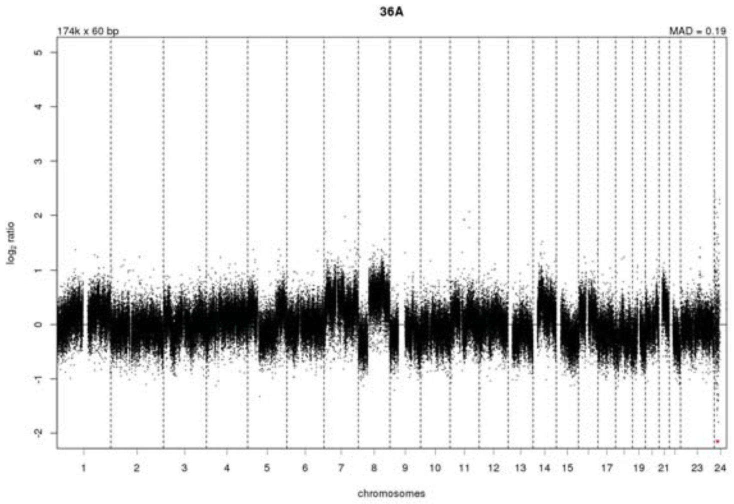
36A
174k x 60 bp
MAD = 0.19
log2 ratio
chromosomes

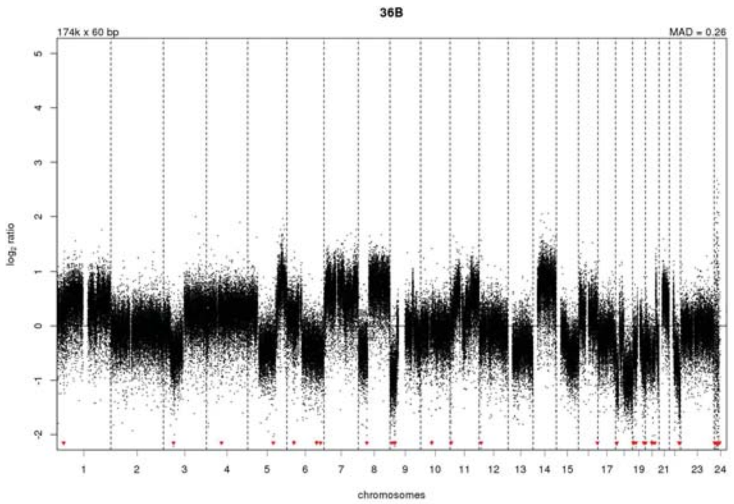
36B
174k x 60 bp
MAD = 0.26
log2 ratio
chromosomes

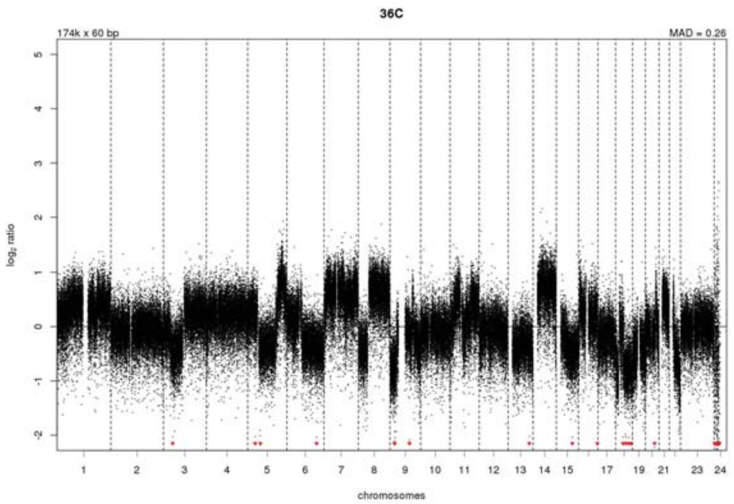
36C
174k x 60 bp
MAD = 0.26
log2 ratio
chromosomes

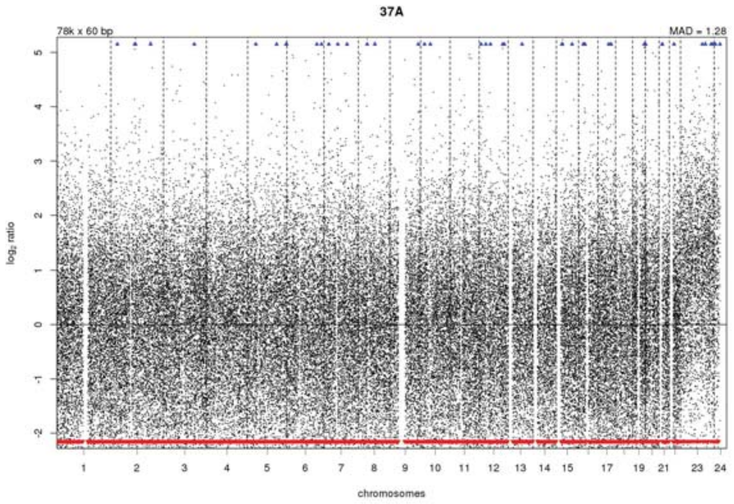
37A
78k x 60 bp
MAD = 1.28
log2 ratio
chromosomes

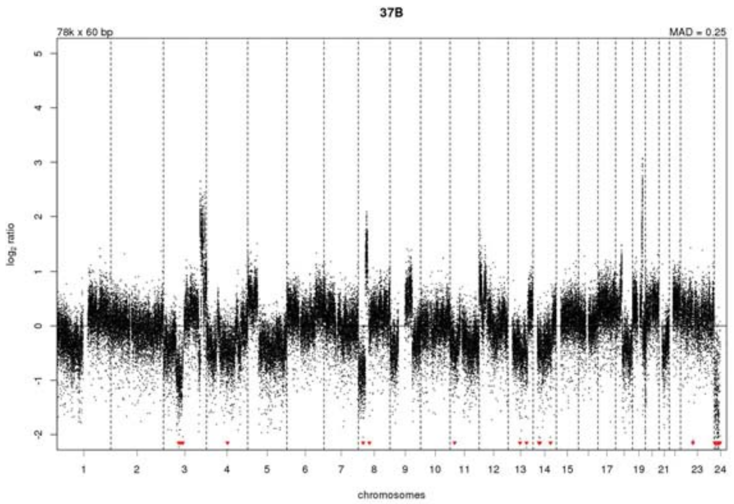
37B
78k x 60 bp
MAD = 0.25
log2 ratio
chromosomes

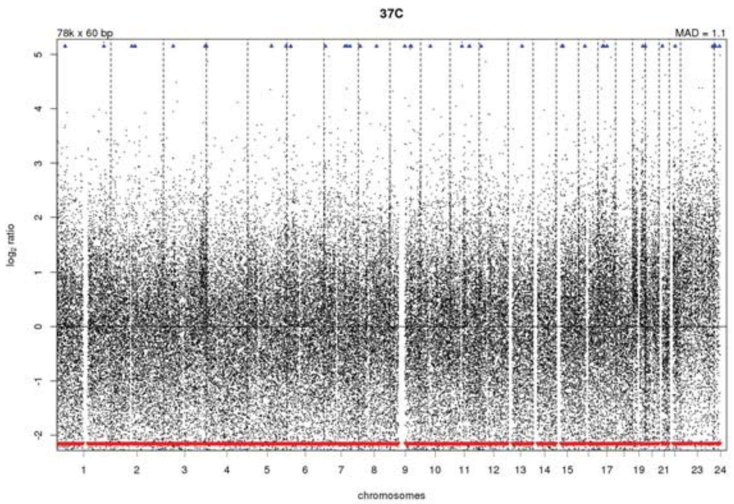
37C
78k x 60 bp
MAD = 1.1
log2 ratio
chromosomes

38A

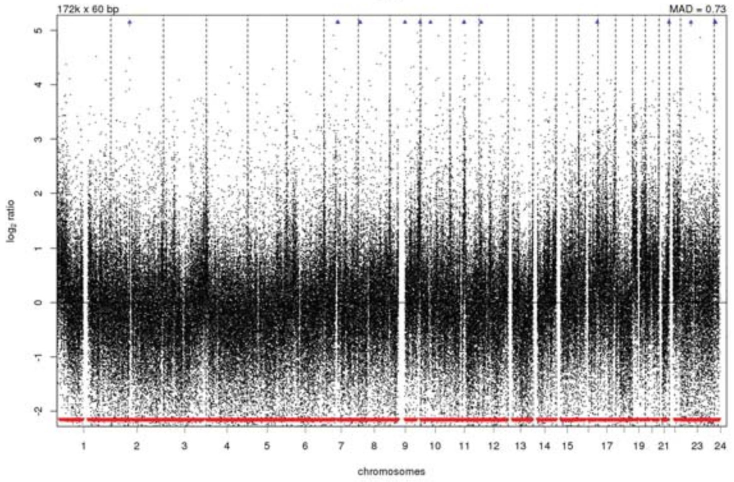
172k x 60 bp
MAD = 0.73
log2 ratio
chromosomes

38B

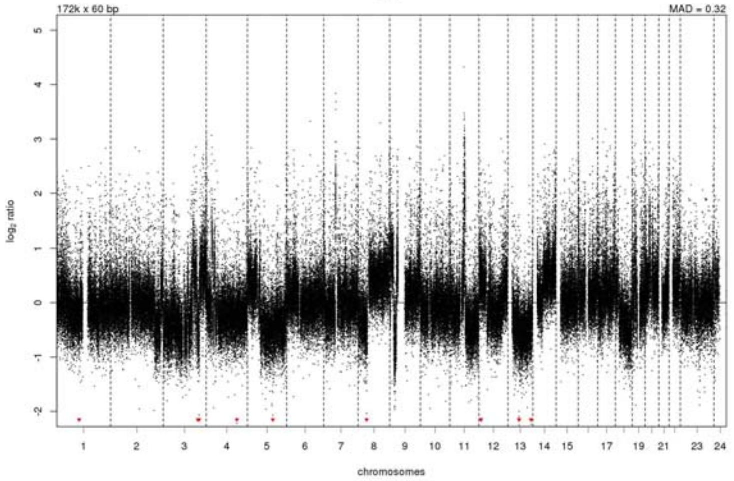
172k x 60 bp
MAD = 0.32
log2 ratio
chromosomes

38C

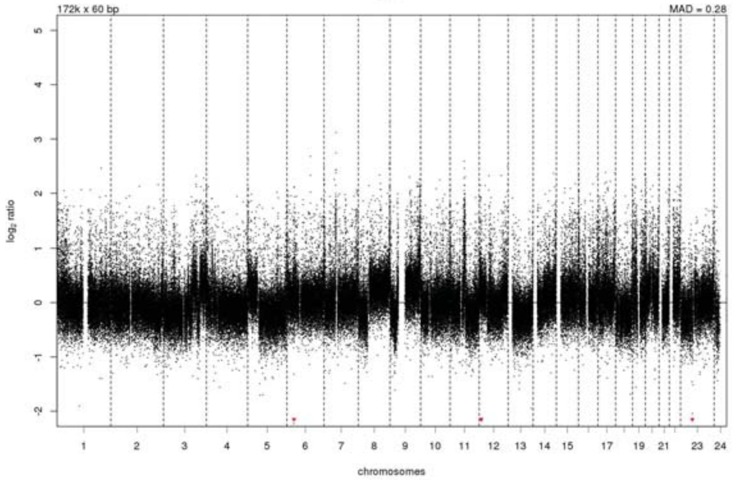
172k x 60 bp
MAD = 0.28
log2 ratio
chromosomes

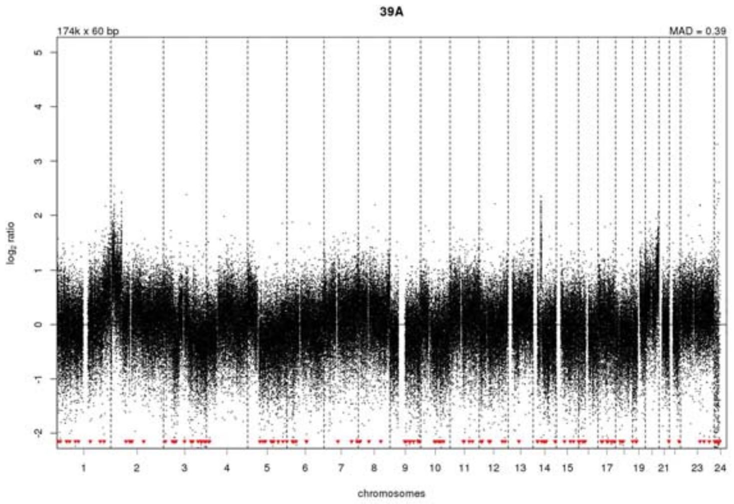
39A
174k x 60 bp
MAD = 0.39
log2 ratio
chromosomes

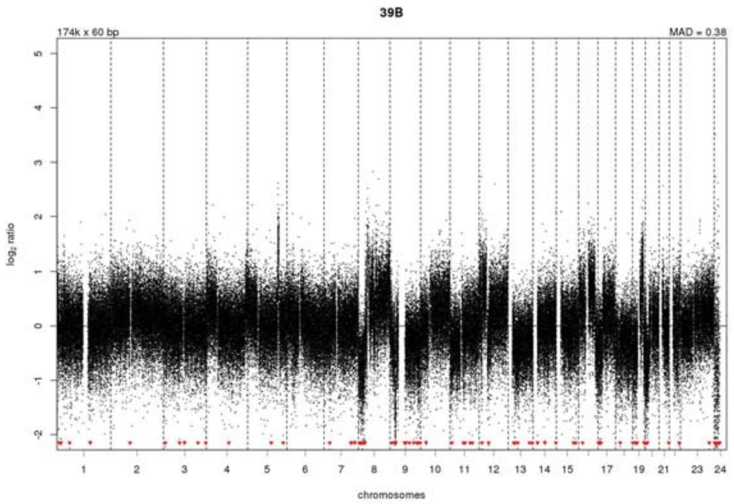
39B
174k x 60 bp
MAD = 0.38
log2 ratio
chromosomes

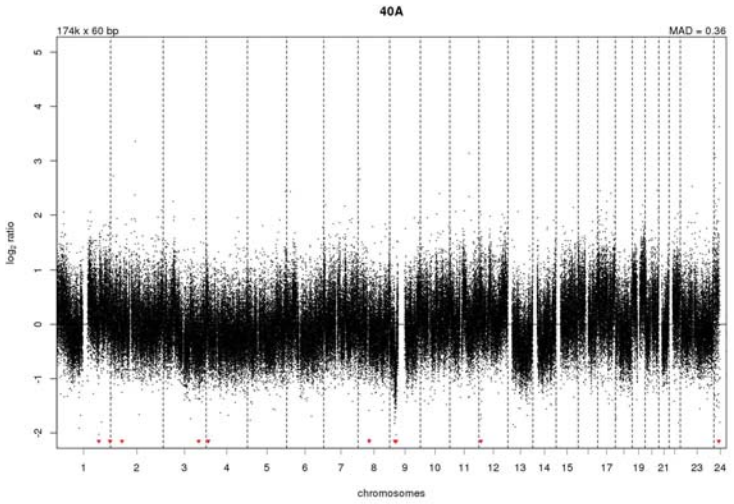
40A
174k x 60 bp
MAD = 0.36
log2 ratio
chromosomes

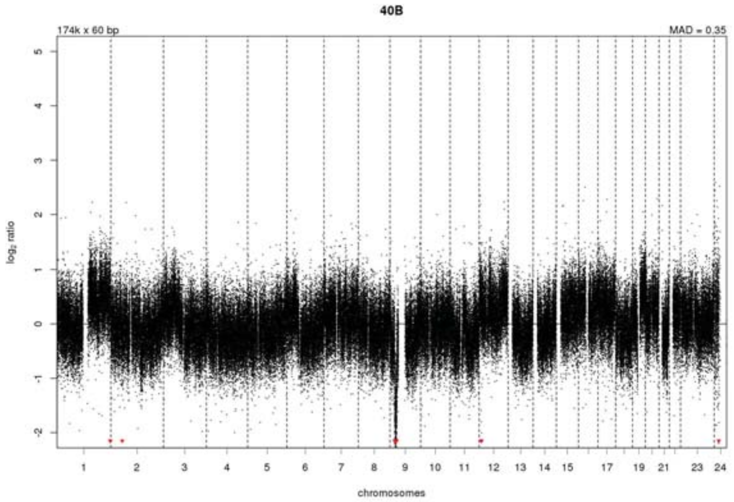
40B
174k x 60 bp
MAD = 0.35
log2 ratio
chromosomes

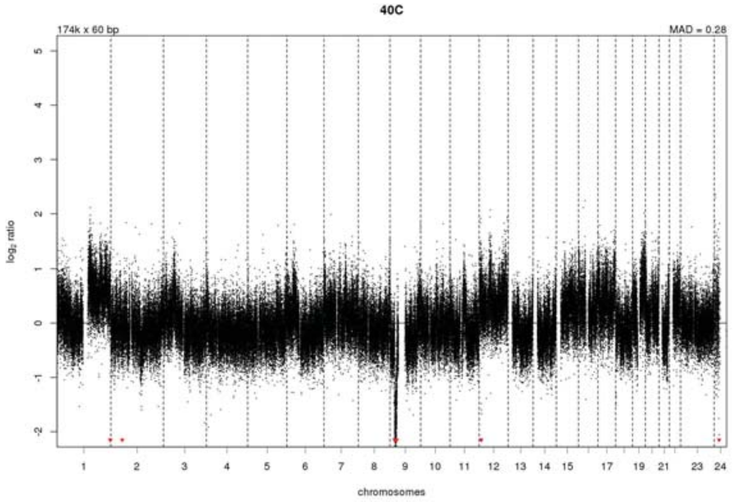
40C
174k x 60 bp
MAD = 0.28
log2 ratio
chromosomes

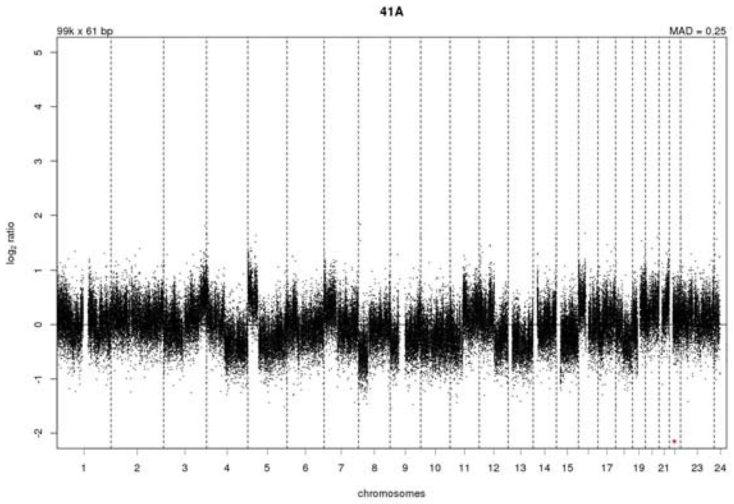
41A
99k x 61 bp
MAD = 0.25
log2 ratio
chromosomes

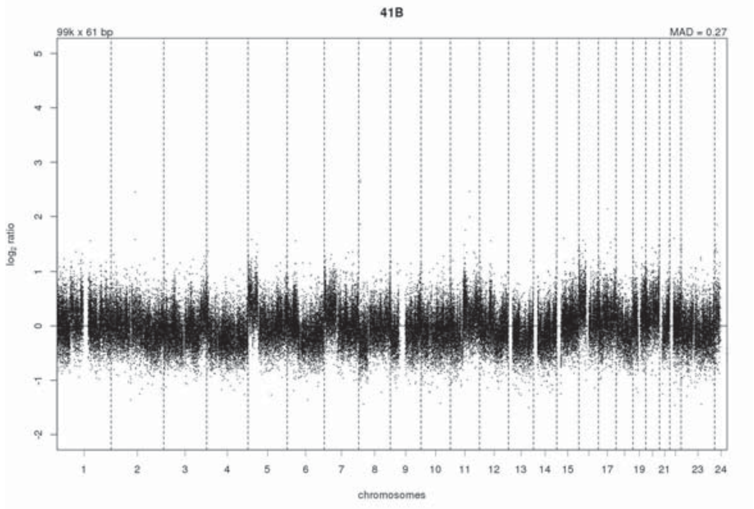
41B
99k x 61 bp
MAD = 0.27
log2 ratio
chromosomes

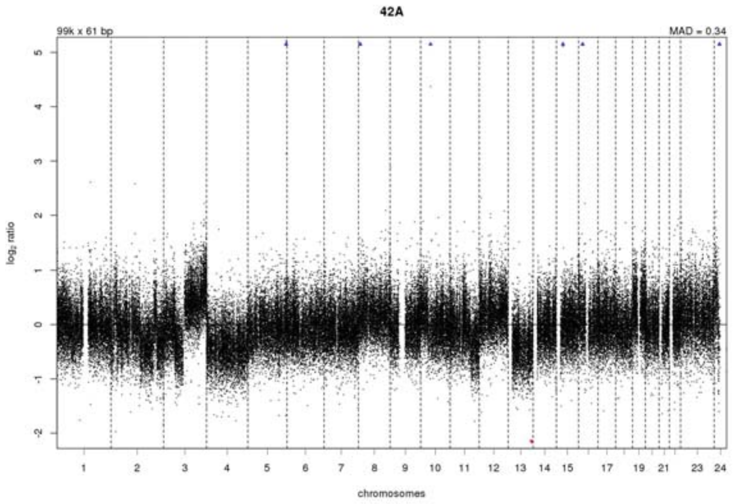
42A
99k x 61 bp
MAD = 0.34
log2 ratio
chromosomes

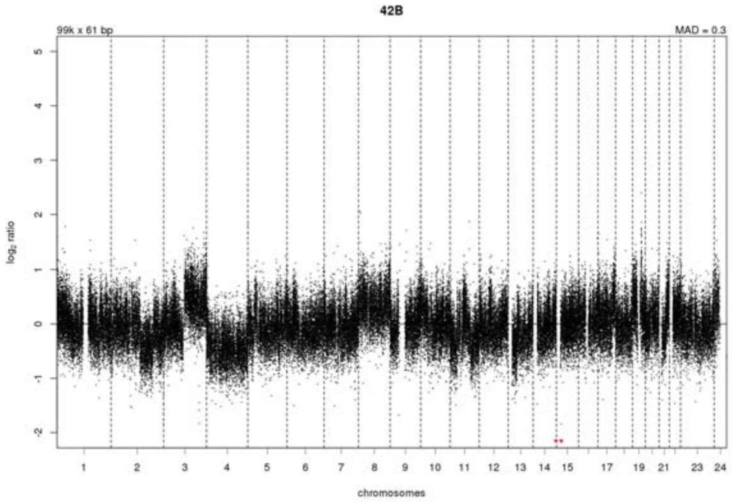
42B
99k x 61 bp
MAD = 0.3
log2 ratio
chromosomes

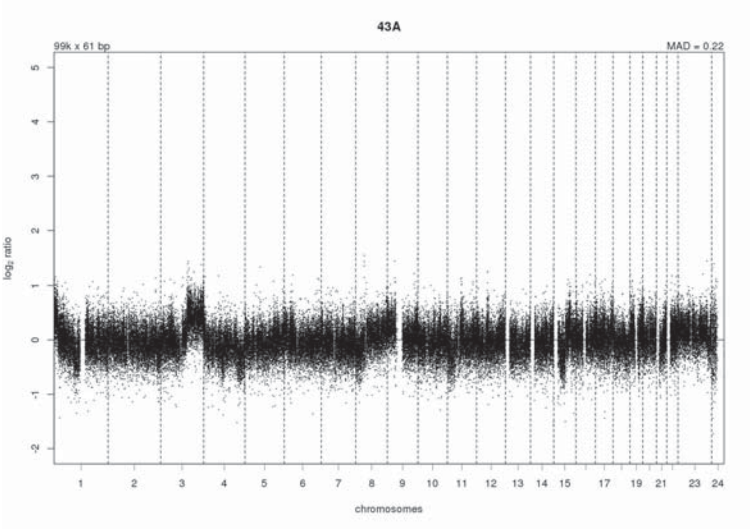
43A
99k x 61 bp
MAD = 0.22
log2 ratio
chromosomes

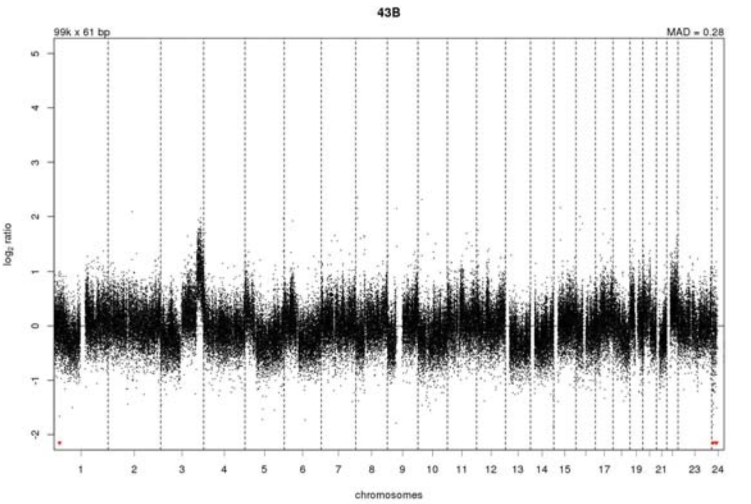
43B
99k x 61 bp
MAD = 0.28
log2 ratio
chromosomes

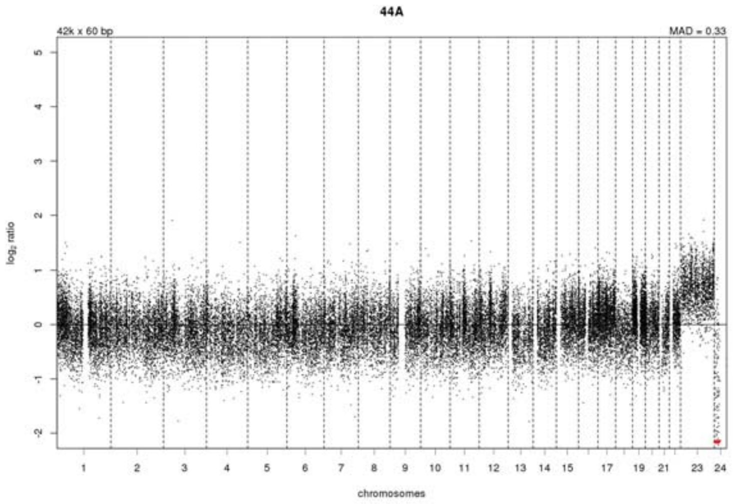
44A
42k x 60 bp
MAD = 0.33
log2 ratio
chromosomes

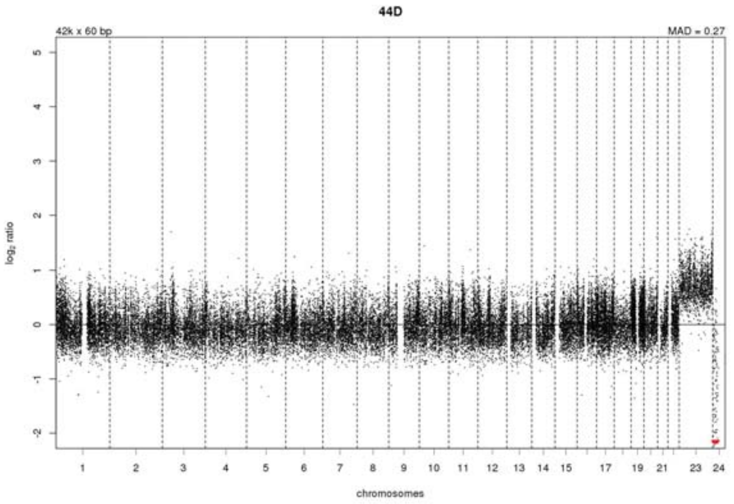
44D
42k x 60 bp
MAD = 0.27
log2 ratio
chromosomes

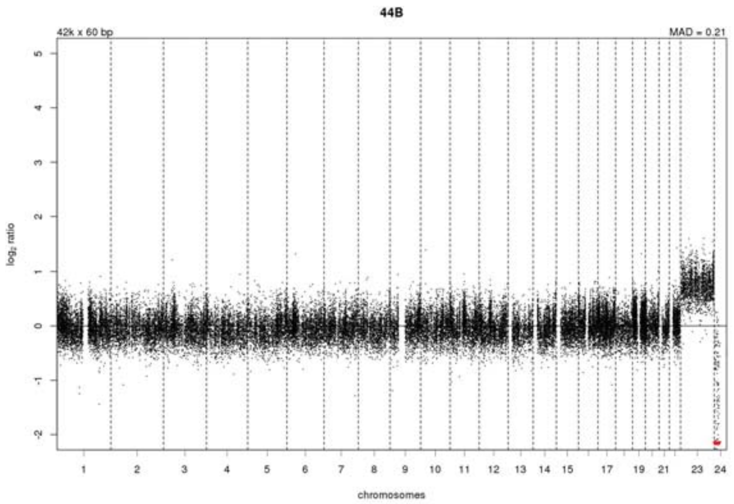
44B
42k x 60 bp
MAD = 0.21
log2 ratio
chromosomes

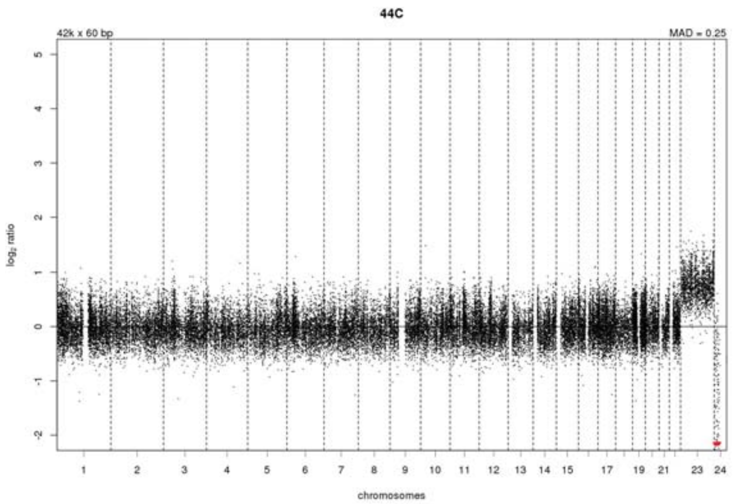
44C
42k x 60 bp
MAD = 0.25
log2 ratio
chromosomes

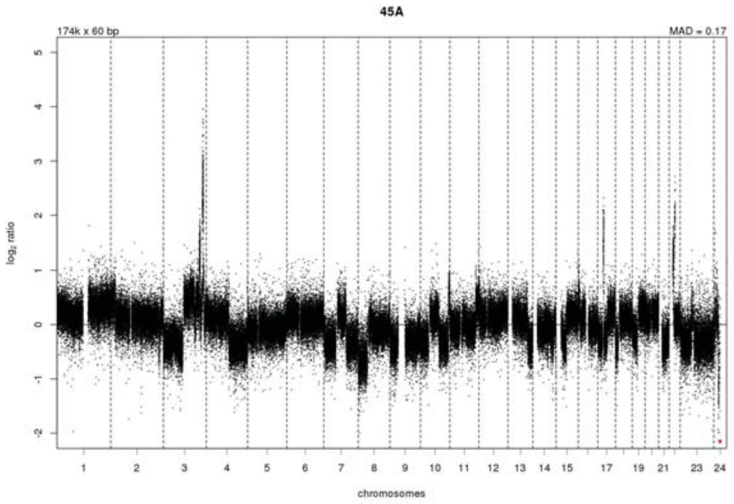
45A
174k x 60 bp
MAD = 0.17
log2 ratio
chromosomes

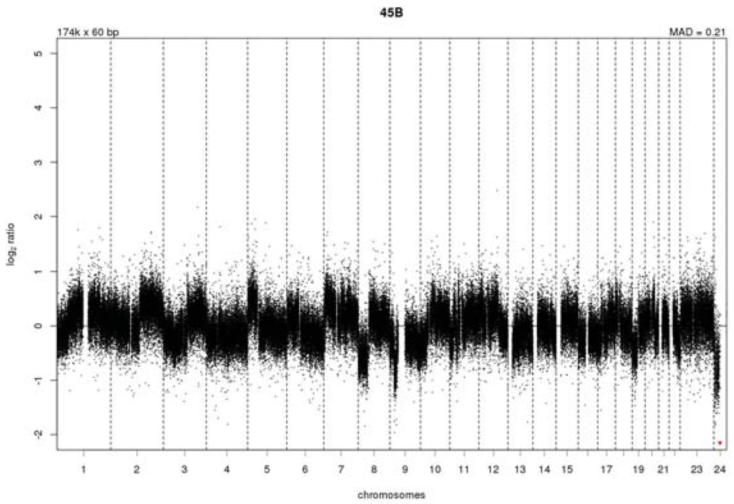
45B
174k x 60 bp
MAD = 0.21
log2 ratio
chromosomes

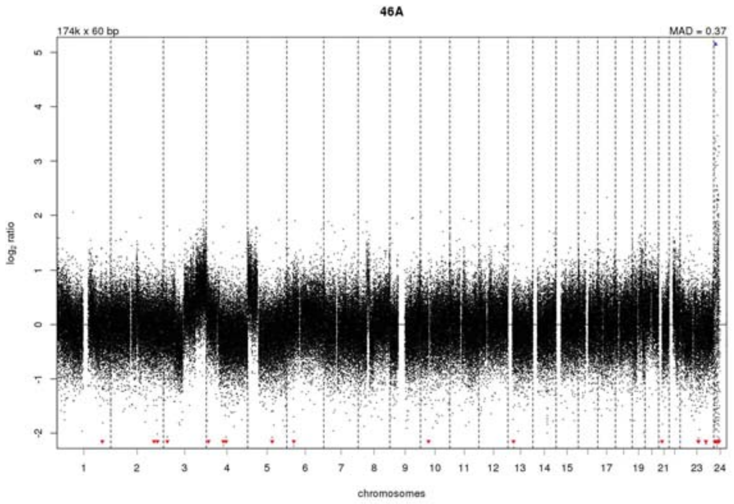
46A
174k x 60 bp
MAD = 0.37
log2 ratio
chromosomes

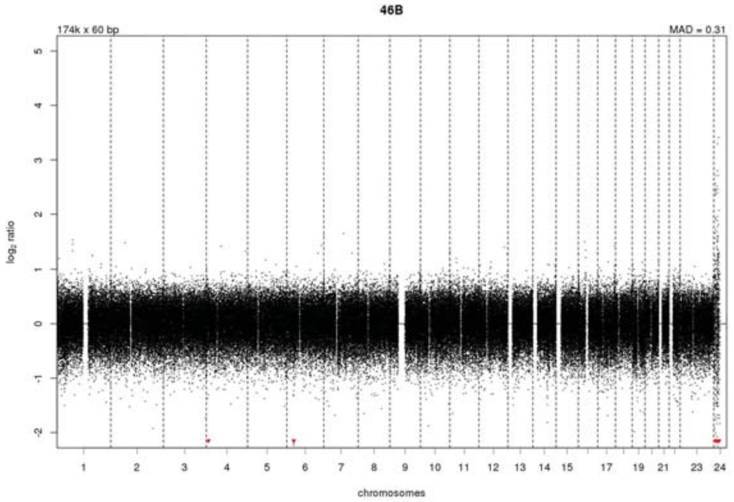
46B
174k x 60 bp
MAD = 0.31
log2 ratio
chromosomes

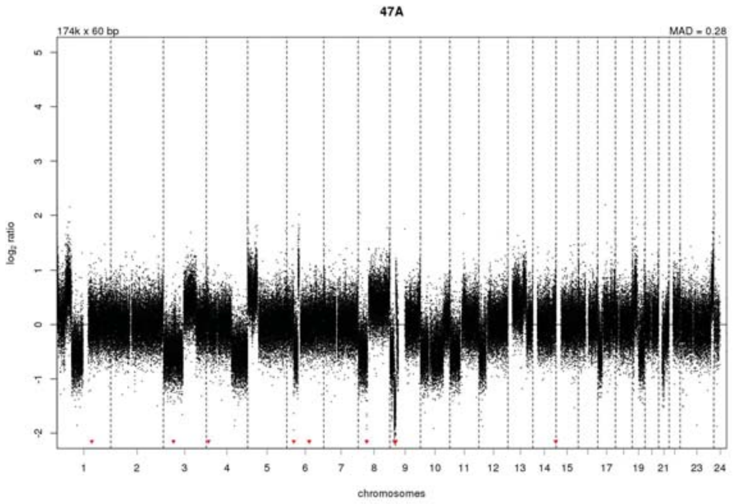
47A
174k x 60 bp
MAD = 0.28
log2 ratio
chromosomes

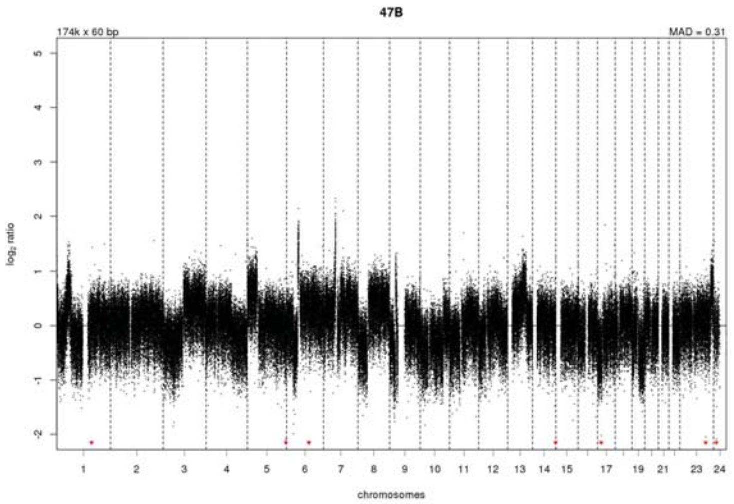
47B
174k x 60 bp
MAD = 0.31
log2 ratio
chromosomes

48A

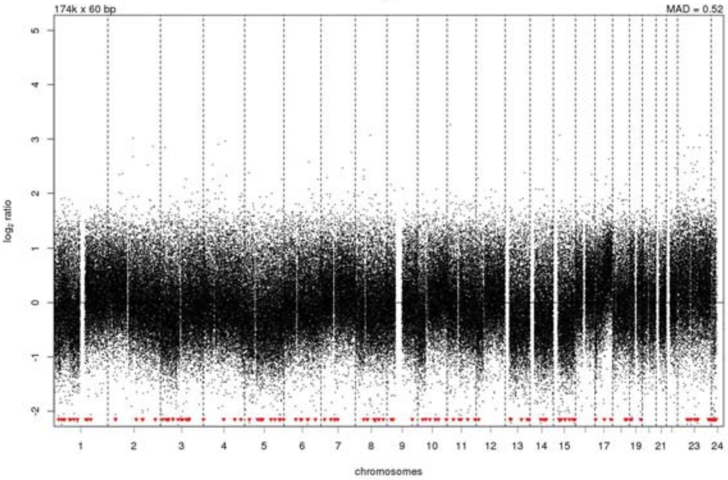
174k x 60 bp
MAD = 0.52
log2 ratio
chromosomes
1
2
3
4
5
6
7
8
9
10
11
12
13
14
15
17
19
21
23
24

48B

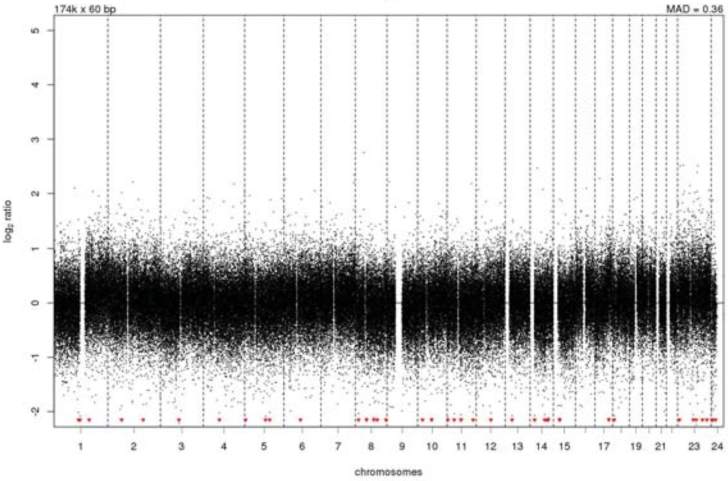
174k x 60 bp
MAD = 0.36
log2 ratio
chromosomes
1
2
3
4
5
6
7
8
9
10
11
12
13
14
15
17
19
21
23
24

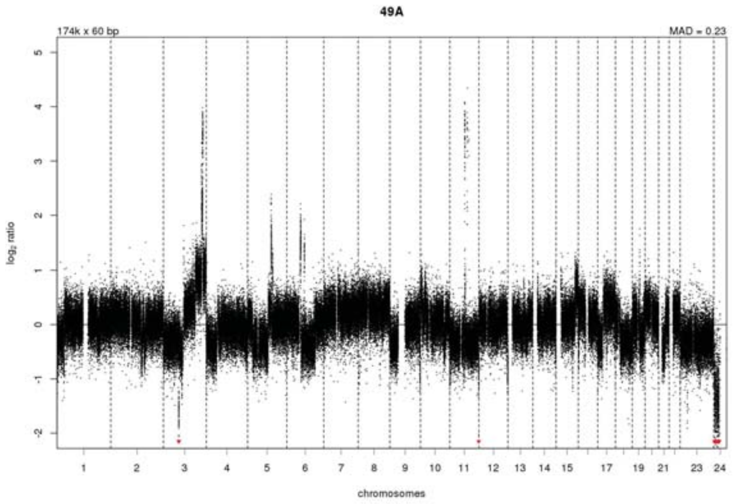
49A
174k x 60 bp
MAD = 0.23
log2 ratio
5
4
3
2
1
0
-1
-2
1
2
3
4
5
6
7
8
9
10
11
12
13
14
15
17
19
21
23
24
chromosomes

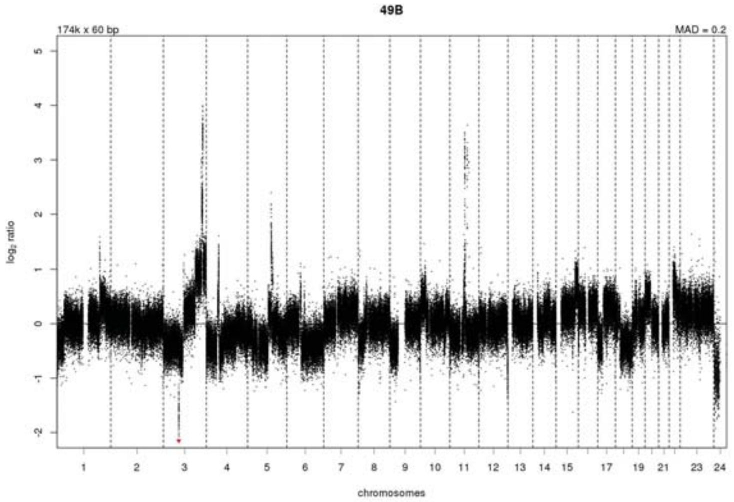
49B
174k x 60 bp
MAD = 0.2
log2 ratio
5
4
3
2
1
0
-1
-2
1
2
3
4
5
6
7
8
9
10
11
12
13
14
15
17
19
21
23
24
chromosomes

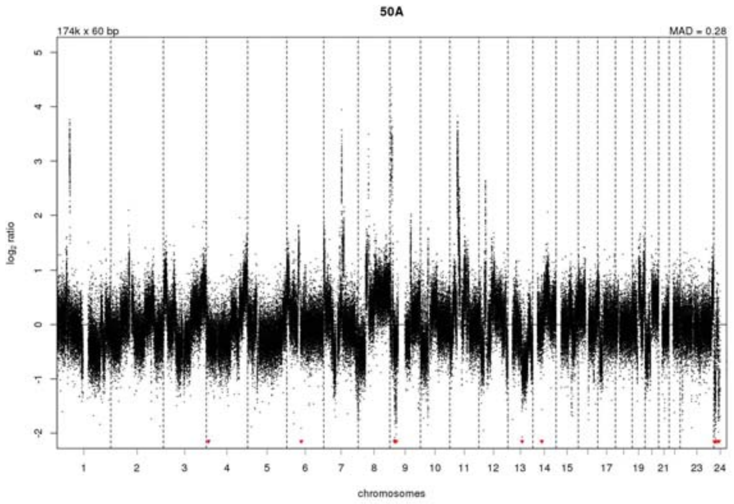
50A
174k x 60 bp
MAD = 0.28
log2 ratio
chromosomes

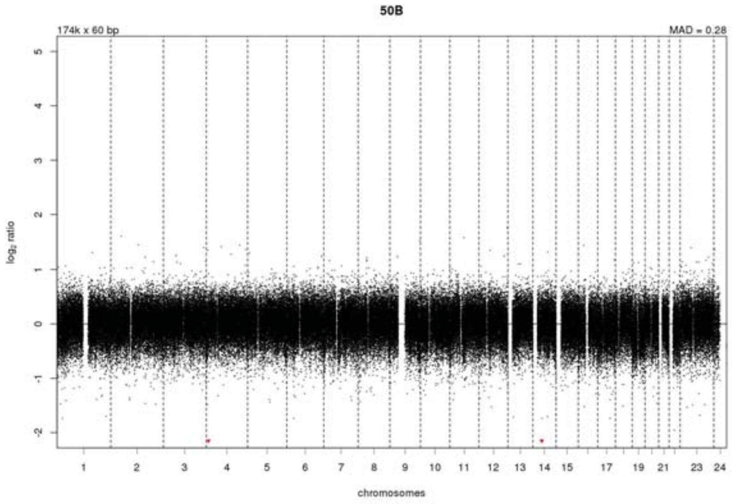
50B
174k x 60 bp
MAD = 0.28
log2 ratio
chromosomes

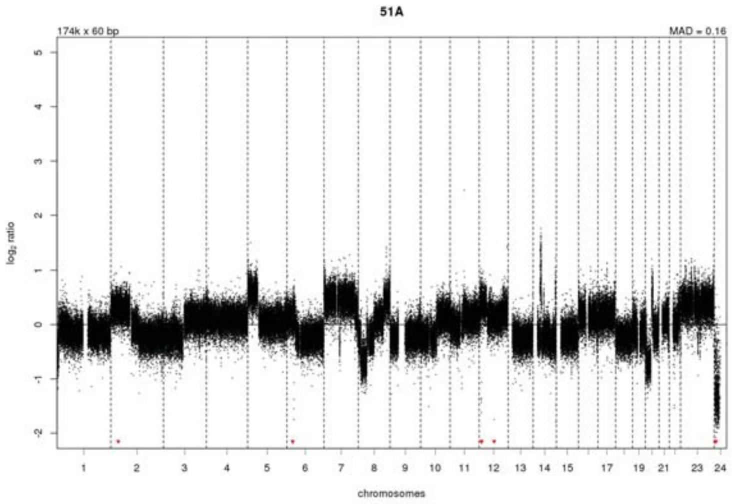

51A
174k x 60 bp
MAD = 0.16
log2 ratio
chromosomes

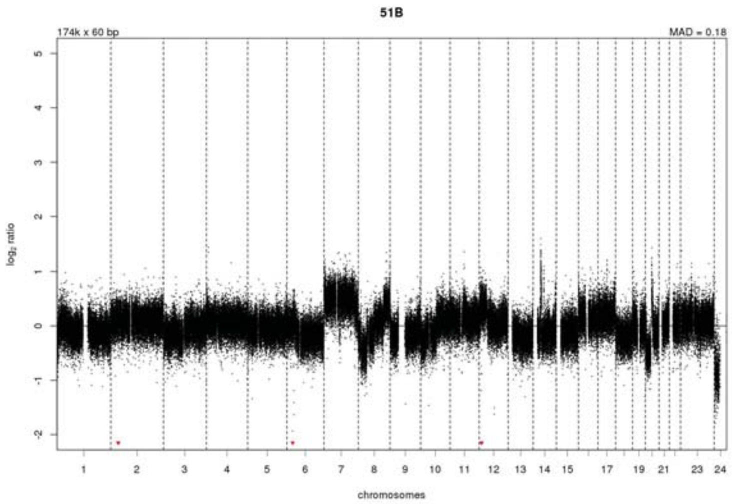

51B
174k x 60 bp
MAD = 0.18
log2 ratio
chromosomes

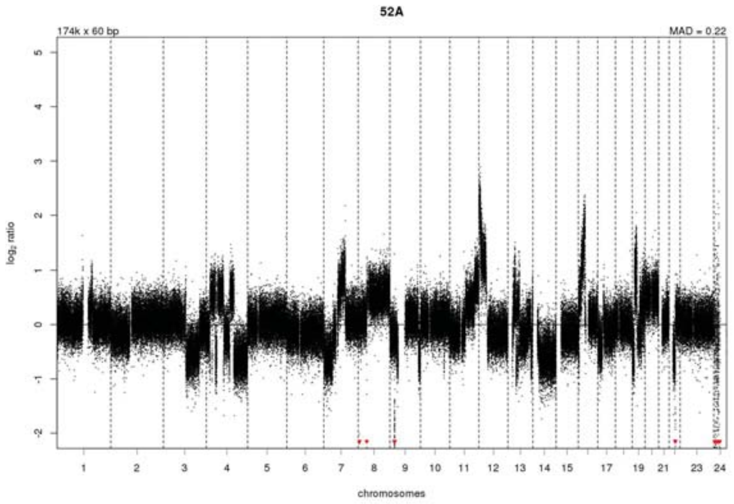
52A
174k x 60 bp
MAD = 0.22
log2 ratio
chromosomes
1 2 3 4 5 6 7 8 9 10 11 12 13 14 15 17 19 21 23 24

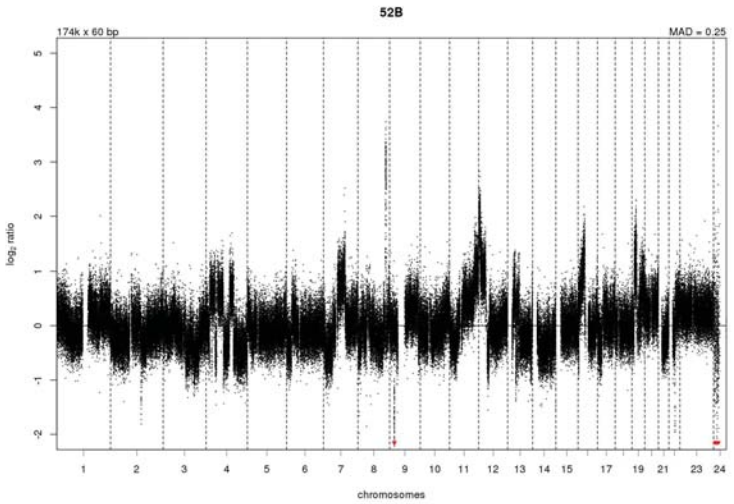
52B
174k x 60 bp
MAD = 0.25
log2 ratio
chromosomes
1 2 3 4 5 6 7 8 9 10 11 12 13 14 15 17 19 21 23 24

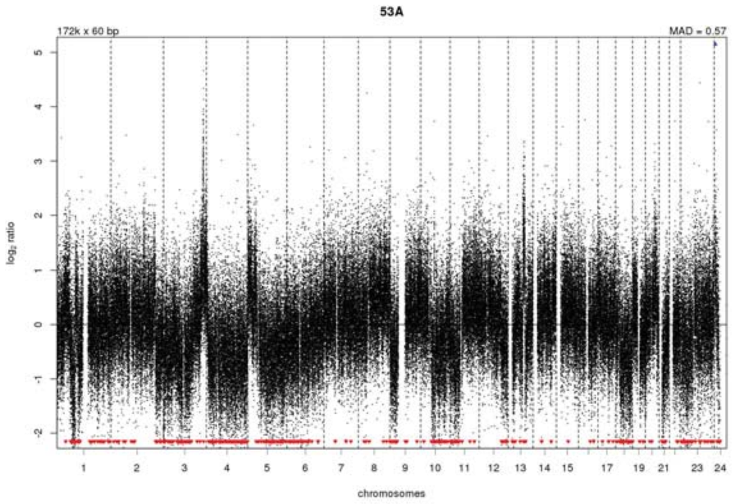
53A
172k x 60 bp
MAD = 0.57
log2 ratio
chromosomes

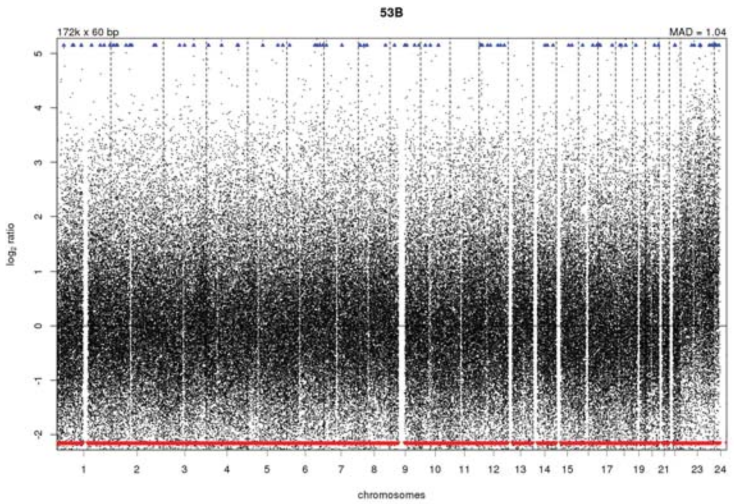
53B
172k x 60 bp
MAD = 1.04
log2 ratio
chromosomes

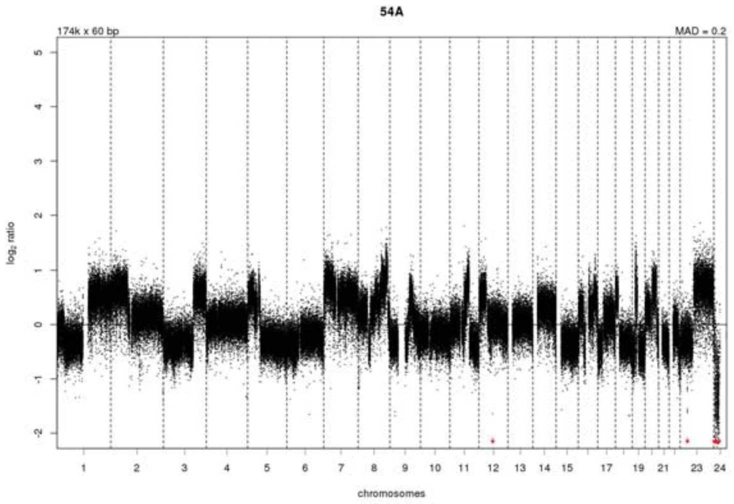
54A
174k x 60 bp
MAD = 0.2
log2 ratio
5
4
3
2
1
0
-1
-2
1
2
3
4
5
6
7
8
9
10
11
12
13
14
15
17
19
21
23
24
chromosomes

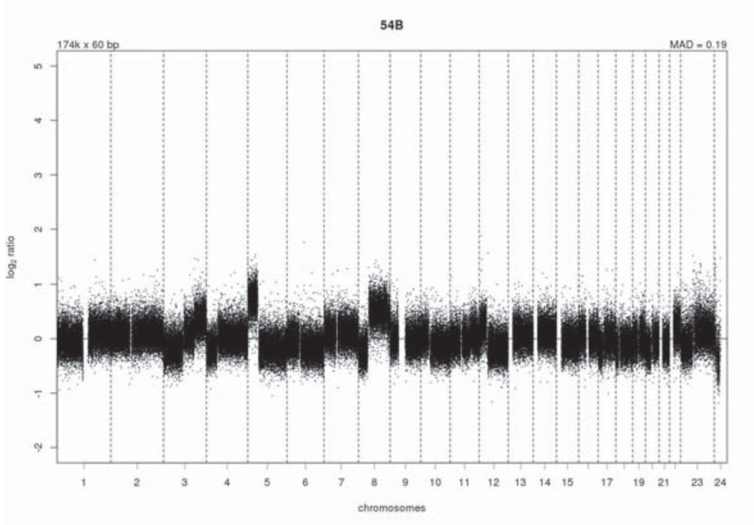
54B
174k x 60 bp
MAD = 0.19
log2 ratio
5
4
3
2
1
0
-1
-2
1
2
3
4
5
6
7
8
9
10
11
12
13
14
15
17
19
21
23
24
chromosomes

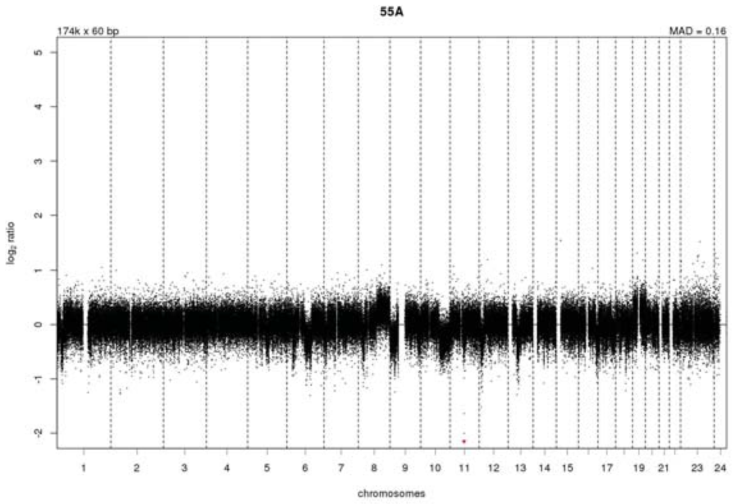

55A
174k x 60 bp
MAD = 0.16
log2 ratio
chromosomes

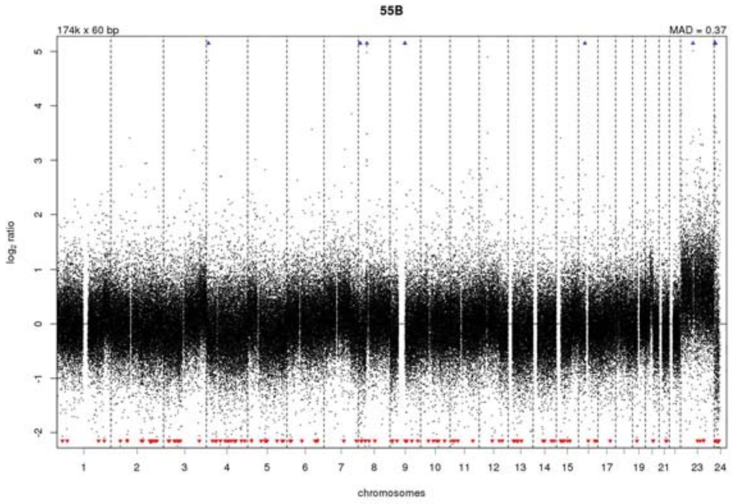

55B
174k x 60 bp
MAD = 0.37
log2 ratio
chromosomes

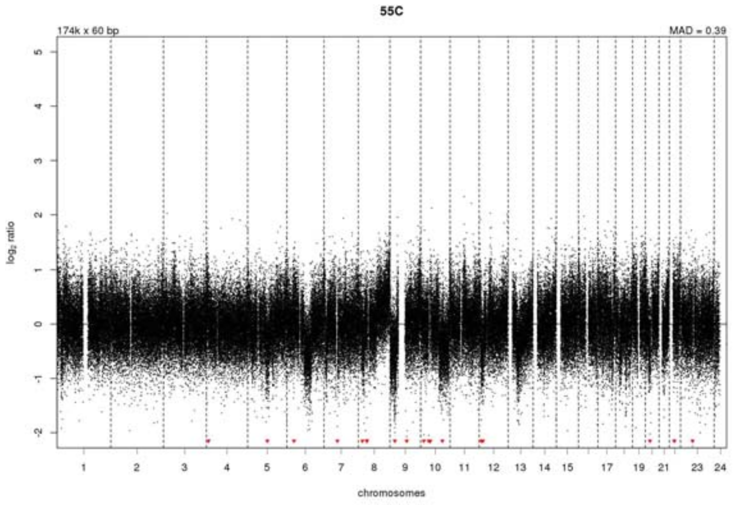

55C
174k x 60 bp
MAD = 0.39
log2 ratio
chromosomes

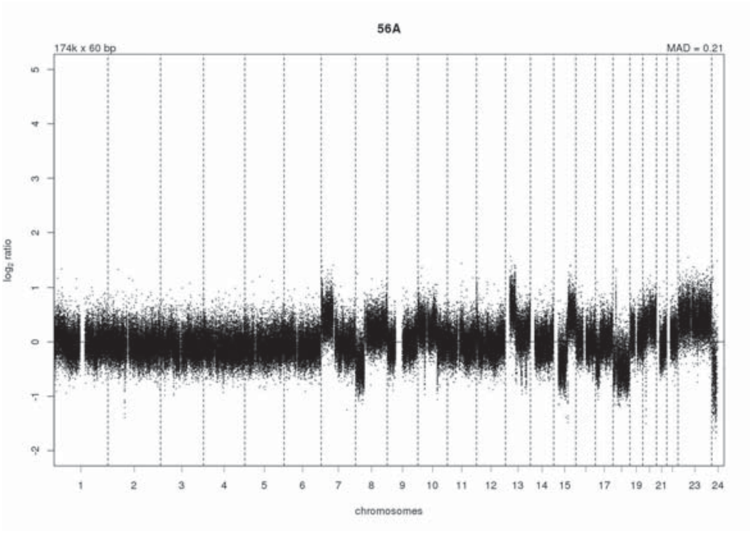
56A
174k x 60 bp
MAD = 0.21
log2 ratio
chromosomes

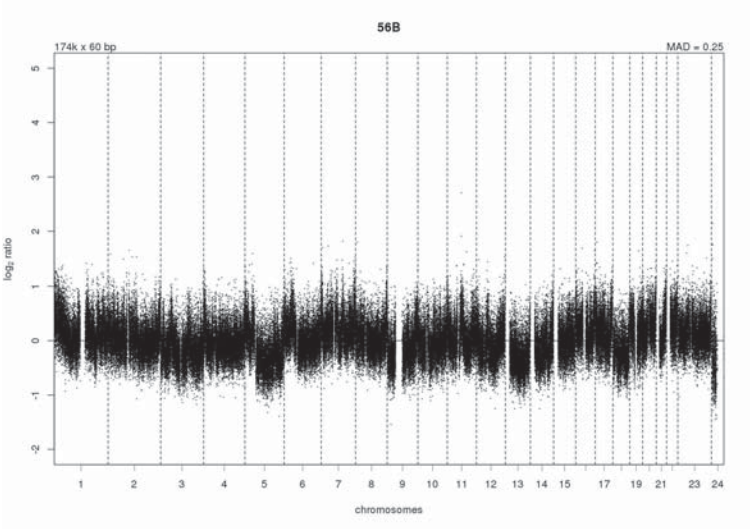
56B
174k x 60 bp
MAD = 0.25
log2 ratio
chromosomes

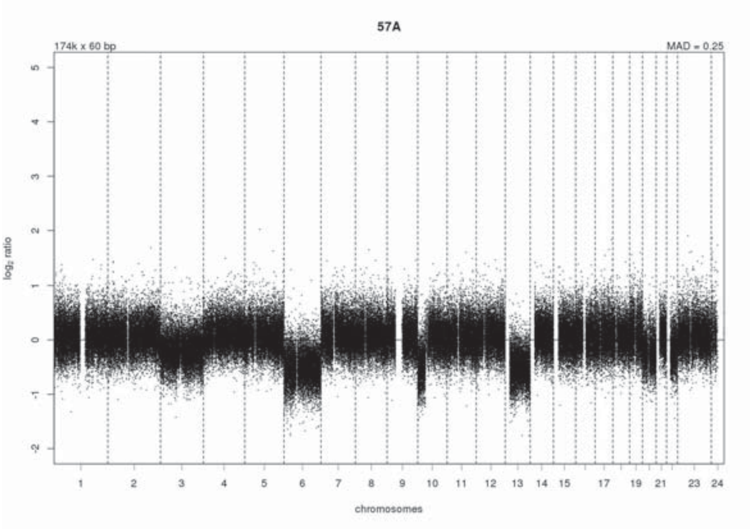
57A
174k x 60 bp
MAD = 0.25
log2 ratio
chromosomes

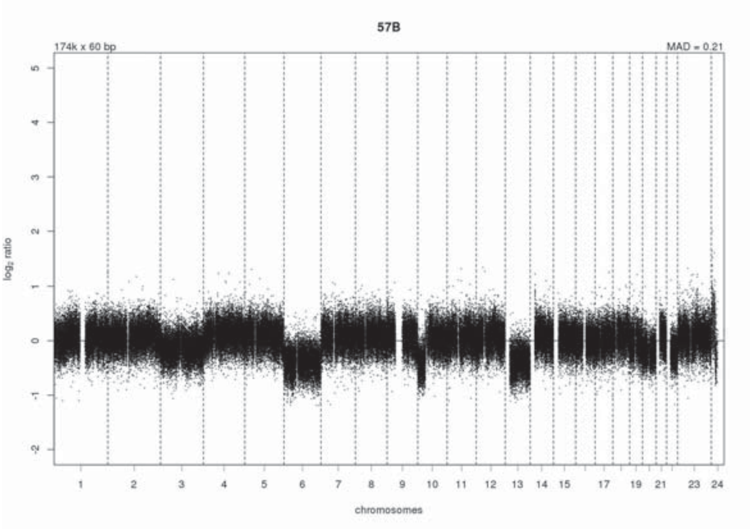
57B
174k x 60 bp
MAD = 0.21
log2 ratio
chromosomes

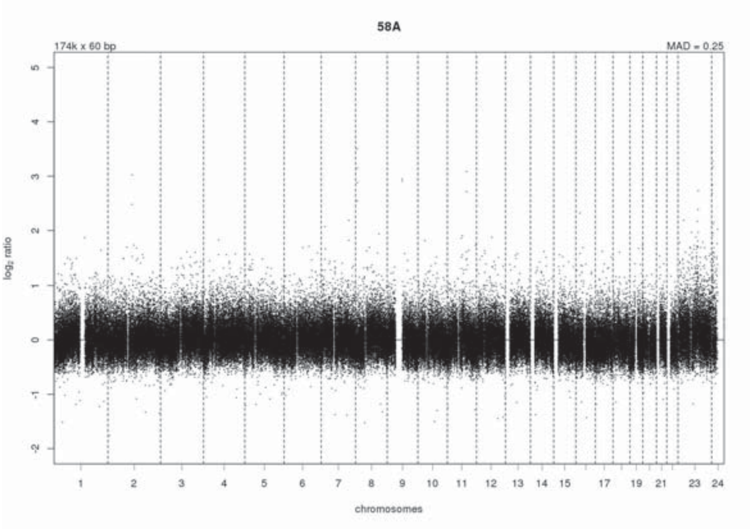
58A
174k x 60 bp
MAD = 0.25
log2 ratio
5
4
3
2
1
0
-1
-2
1
2
3
4
5
6
7
8
9
10
11
12
13
14
15
17
19
21
23
24
chromosomes

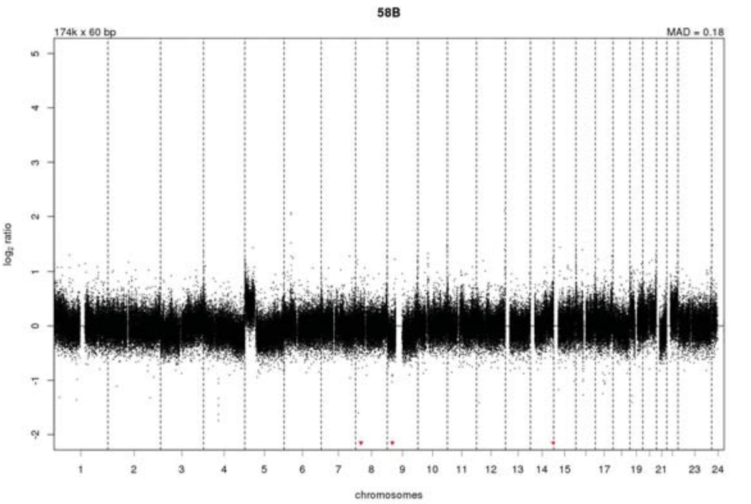
58B
174k x 60 bp
MAD = 0.18
log2 ratio
5
4
3
2
1
0
-1
-2
1
2
3
4
5
6
7
8
9
10
11
12
13
14
15
17
19
21
23
24
chromosomes

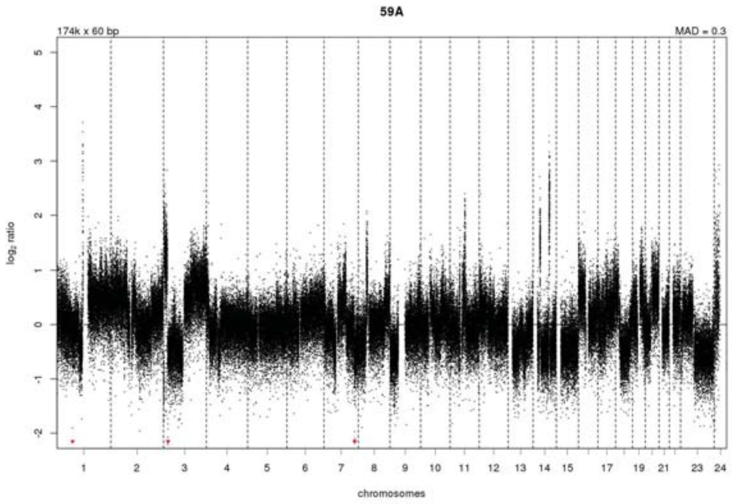
59A
174k x 60 bp
MAD = 0.3
log2 ratio
chromosomes
1 2 3 4 5 6 7 8 9 10 11 12 13 14 15 17 19 21 23 24

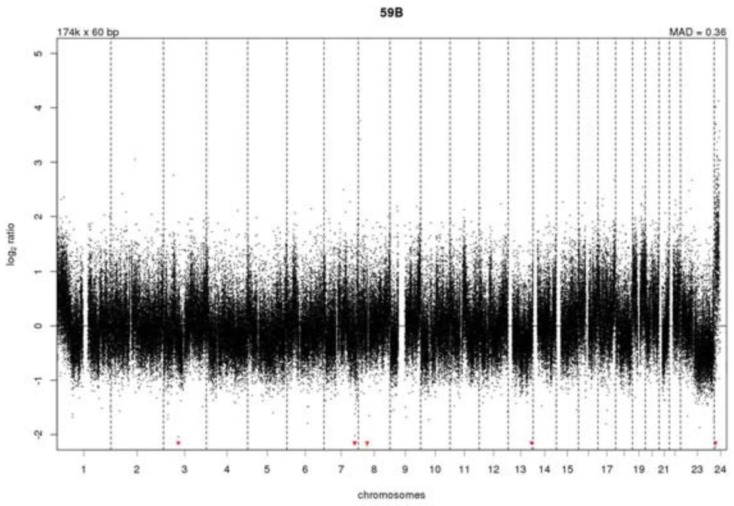
59B
174k x 60 bp
MAD = 0.36
log2 ratio
chromosomes
1 2 3 4 5 6 7 8 9 10 11 12 13 14 15 17 19 21 23 24

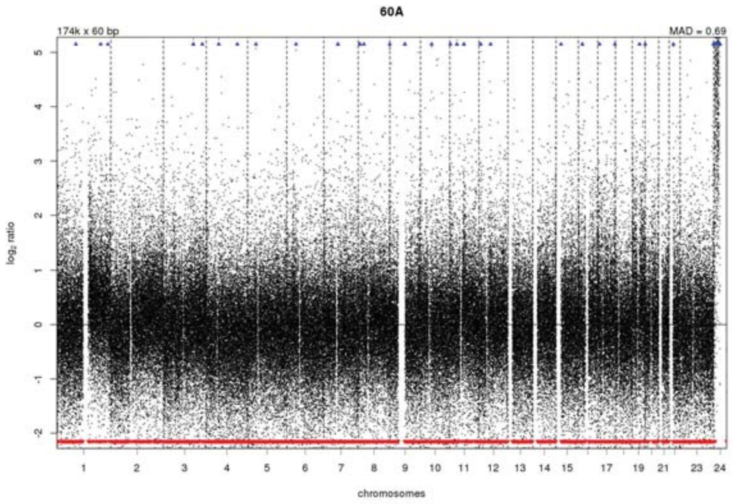
60A
174k x 60 bp
MAD = 0.69
log2 ratio
chromosomes

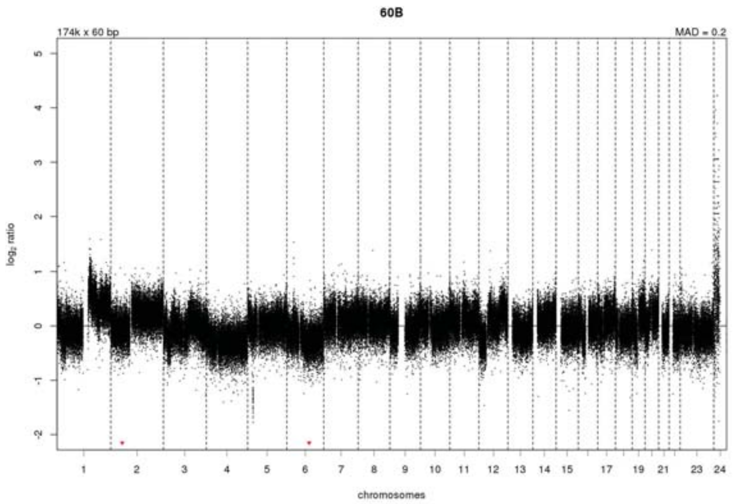
60B
174k x 60 bp
MAD = 0.2
log2 ratio
chromosomes

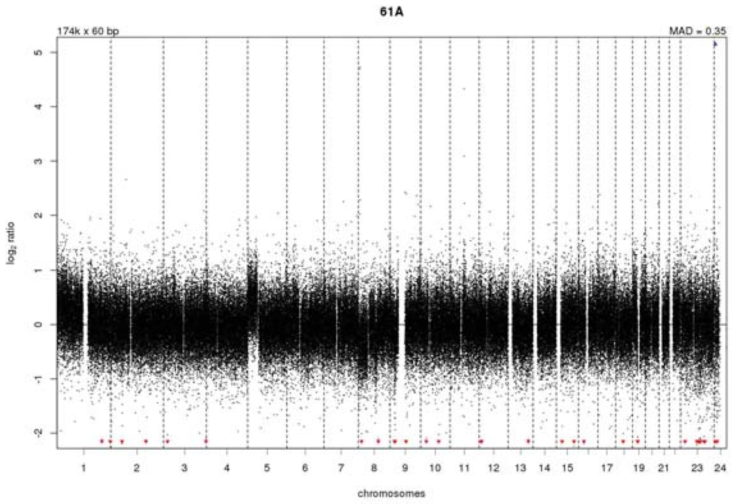

61A
174k x 60 bp
MAD = 0.35
log2 ratio
chromosomes

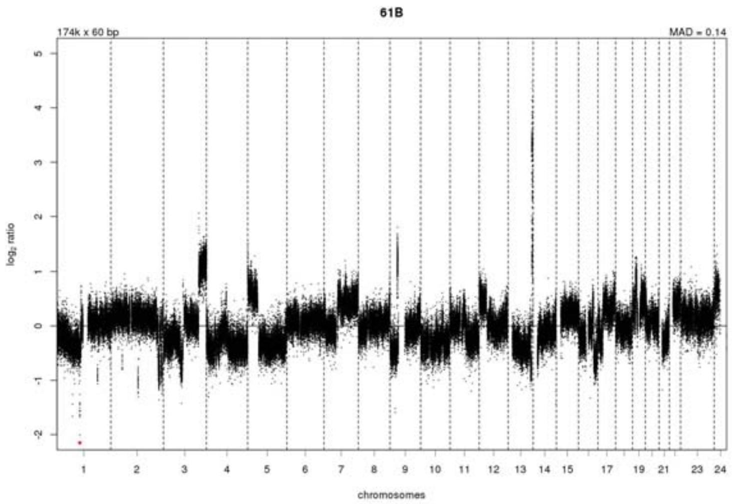

61B
174k x 60 bp
MAD = 0.14
log2 ratio
chromosomes

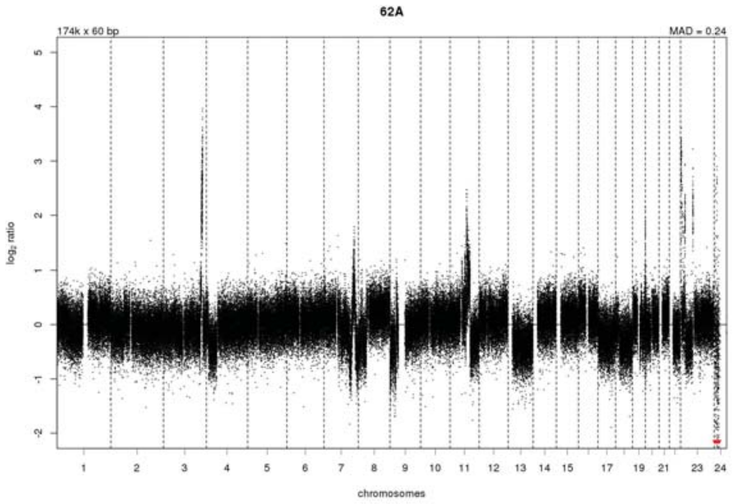
62A
174k x 60 bp
MAD = 0.24
log2 ratio
chromosomes

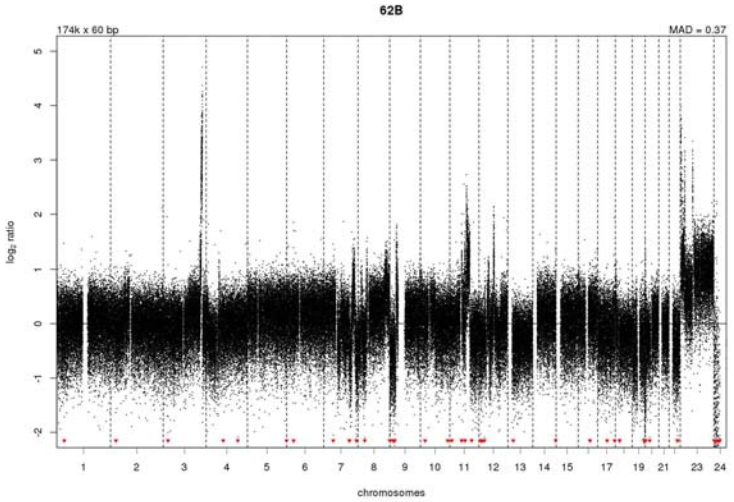
62B
174k x 60 bp
MAD = 0.37
log2 ratio
chromosomes

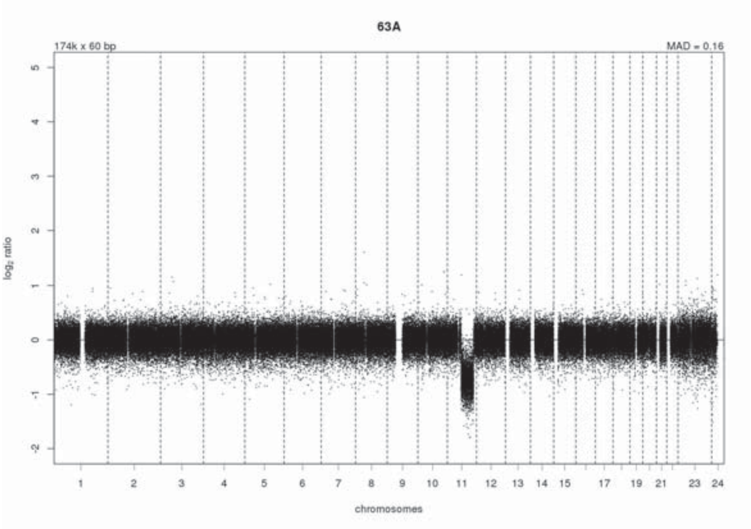
63A
174k x 60 bp
MAD = 0.16
log2 ratio
chromosomes

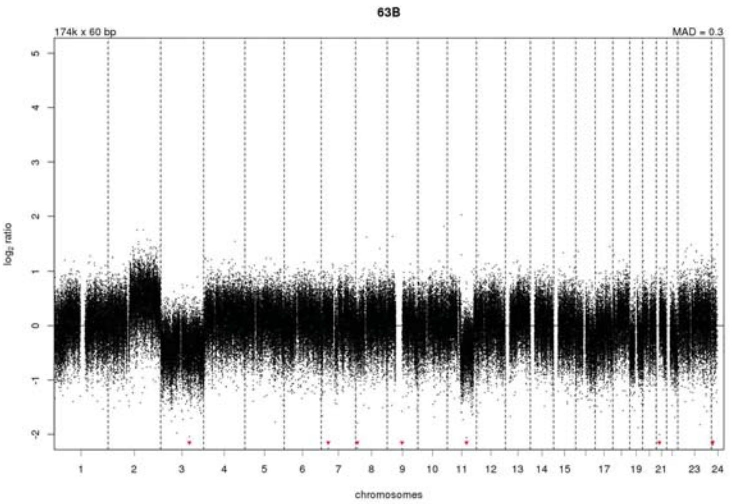
63B
174k x 60 bp
MAD = 0.3
log2 ratio
chromosomes

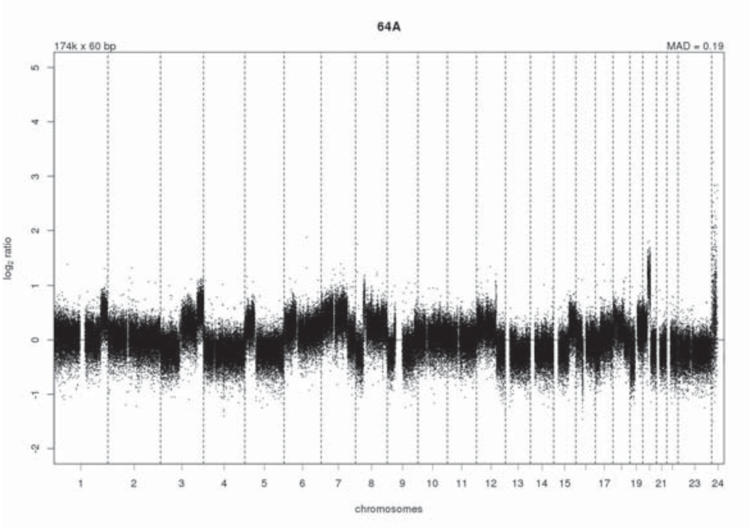
64A
174k x 60 bp
MAD = 0.19
log2 ratio
chromosomes

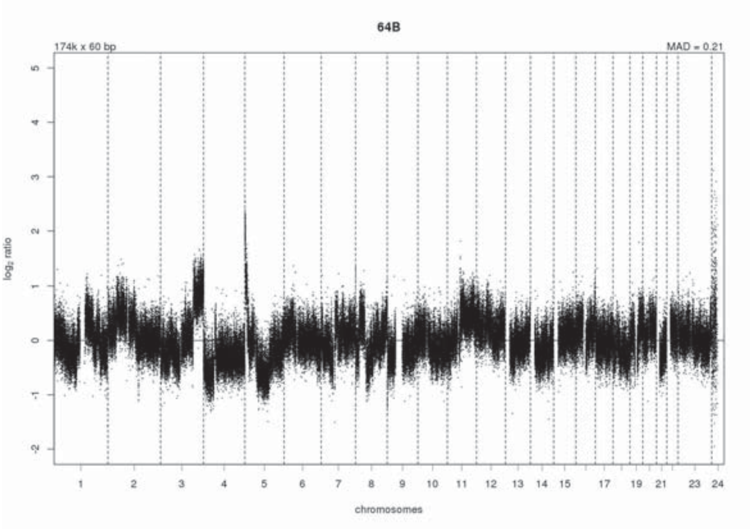
64B
174k x 60 bp
MAD = 0.21
log2 ratio
chromosomes

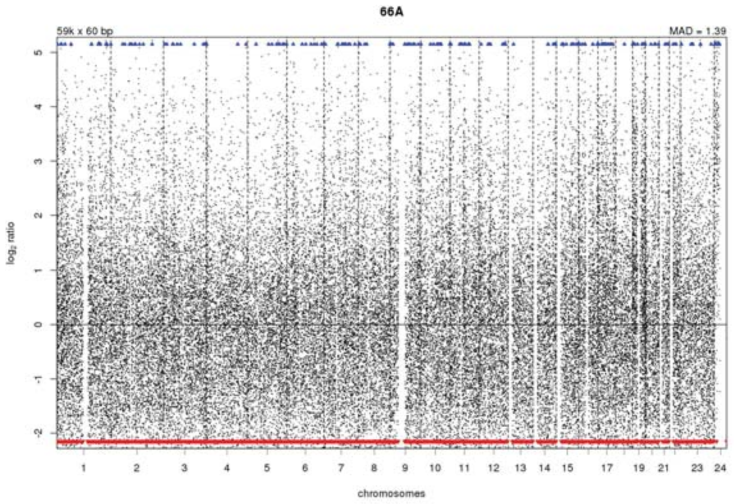
66A
59k x 60 bp
MAD = 1.39
log2 ratio
chromosomes

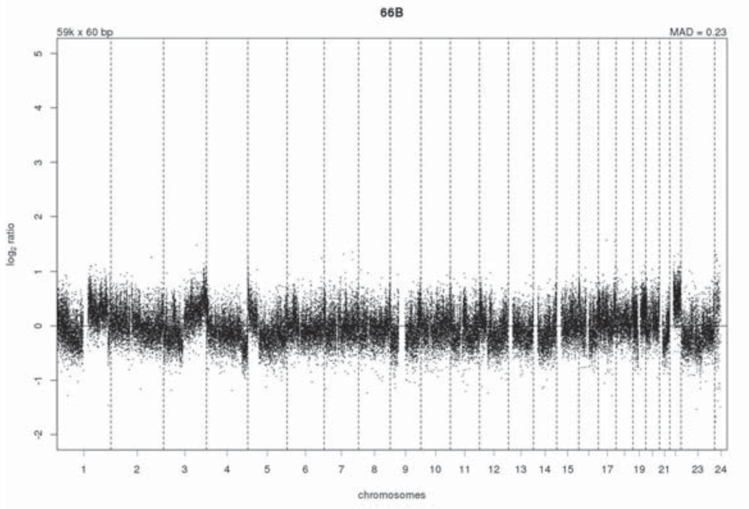
66B
59k x 60 bp
MAD = 0.23
log2 ratio
chromosomes

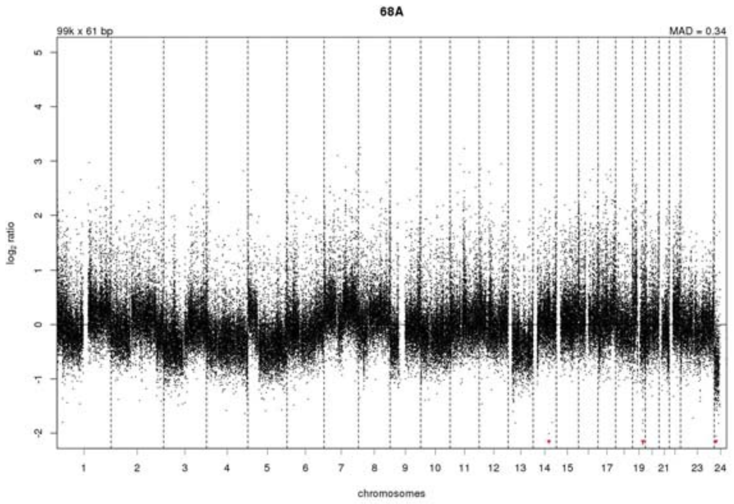
68A
99k x 61 bp
MAD = 0.34
log2 ratio
chromosomes

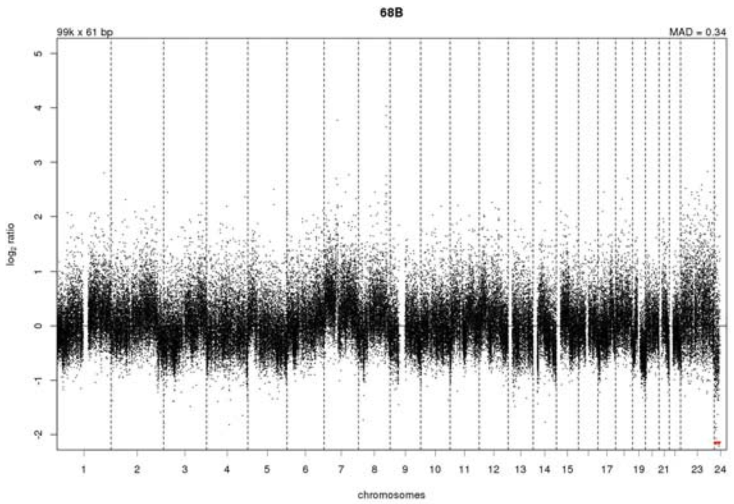
68B
99k x 61 bp
MAD = 0.34
log2 ratio
chromosomes

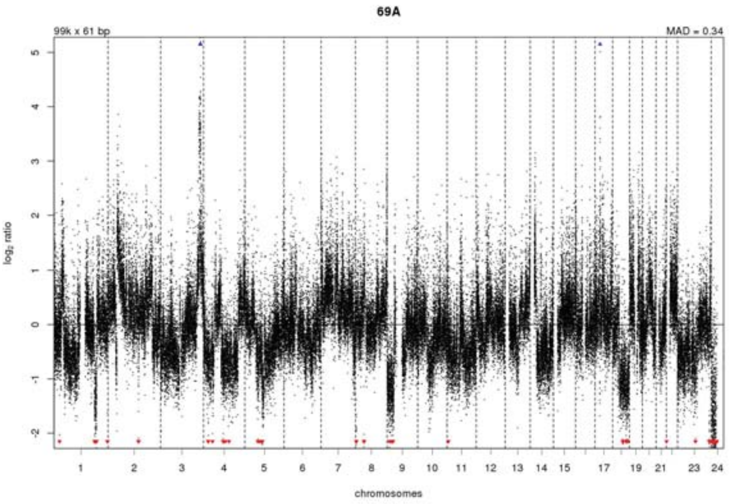
69A
99k x 61 bp
MAD = 0.34
log2 ratio
chromosomes

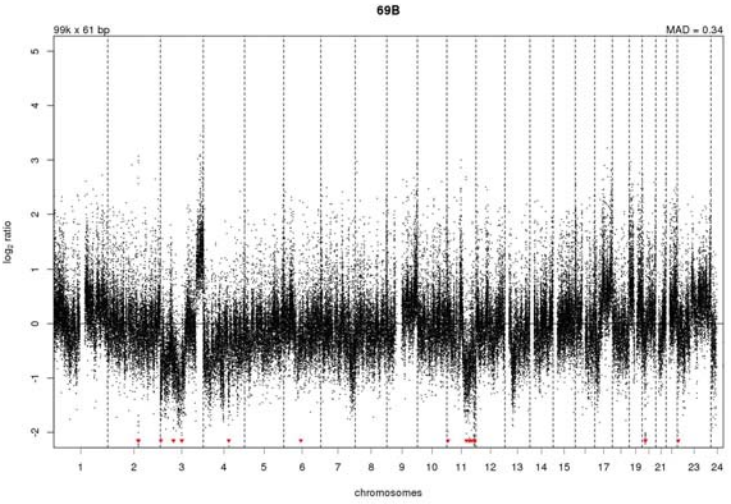
69B
99k x 61 bp
MAD = 0.34
log2 ratio
chromosomes

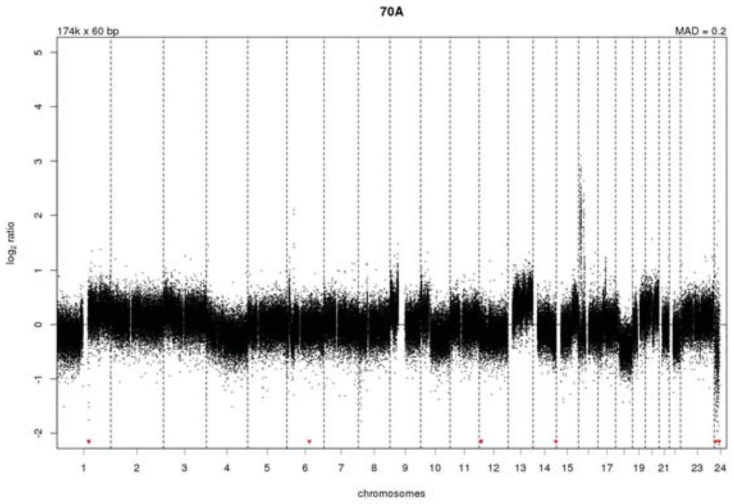
70A
174k x 60 bp
MAD = 0.2
log2 ratio
chromosomes

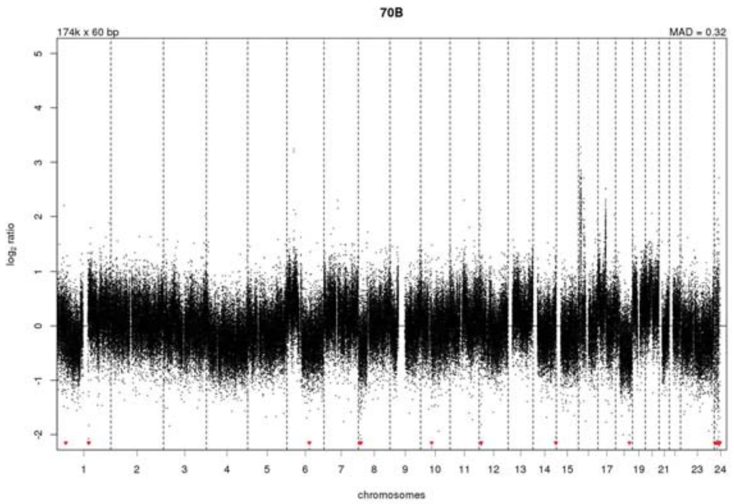
70B
174k x 60 bp
MAD = 0.32
log2 ratio
chromosomes

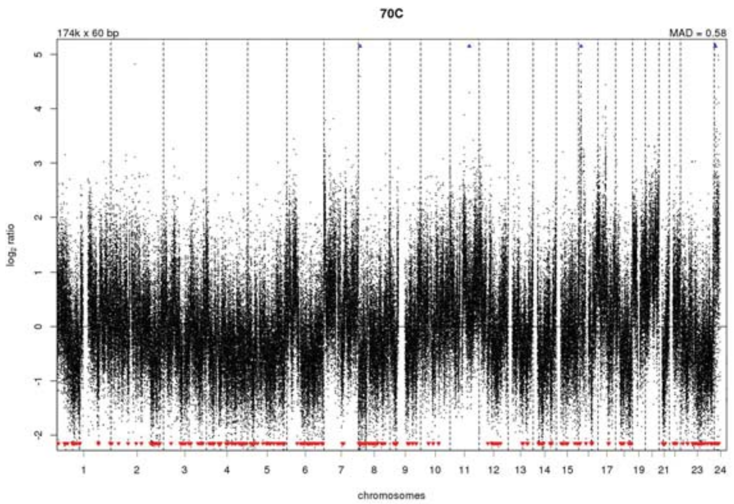
70C
174k x 60 bp
MAD = 0.58
log2 ratio
chromosomes

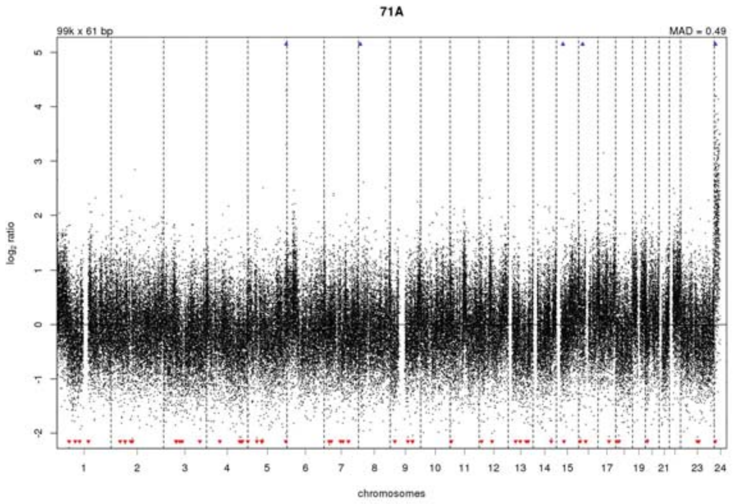
71A
99k x 61 bp
MAD = 0.49
log2 ratio
5 4 3 2 1 0 -1 -2
1 2 3 4 5 6 7 8 9 10 11 12 13 14 15 17 19 21 23 24
chromosomes

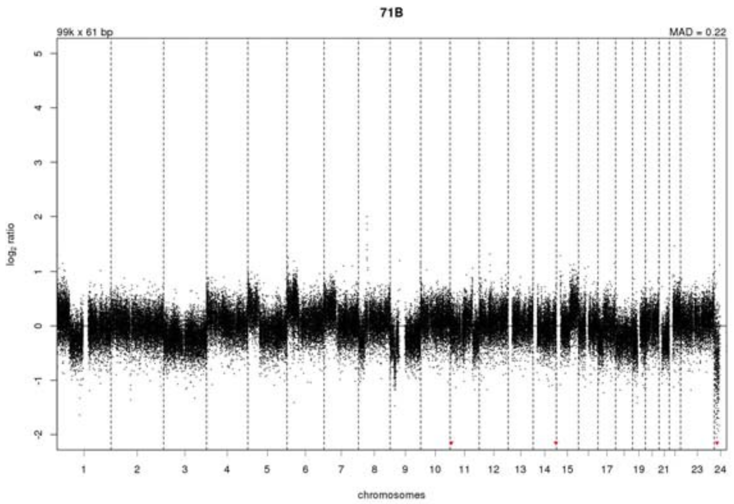
71B
99k x 61 bp
MAD = 0.22
log2 ratio
5 4 3 2 1 0 -1 -2
1 2 3 4 5 6 7 8 9 10 11 12 13 14 15 17 19 21 23 24
chromosomes

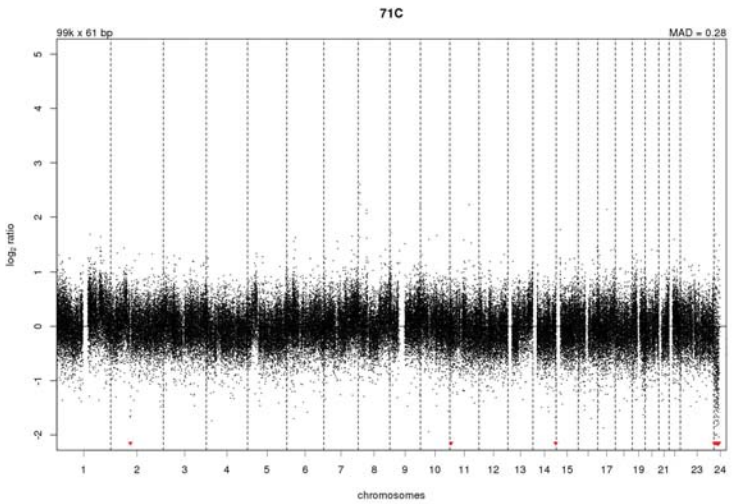
71C
99k x 61 bp
MAD = 0.28
log2 ratio
5 4 3 2 1 0 -1 -2
1 2 3 4 5 6 7 8 9 10 11 12 13 14 15 17 19 21 23 24
chromosomes

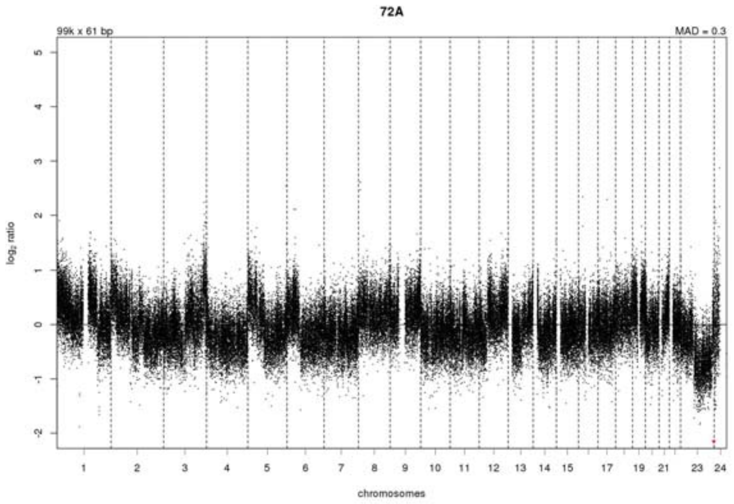
72A
99k x 61 bp
MAD = 0.3
log2 ratio
chromosomes

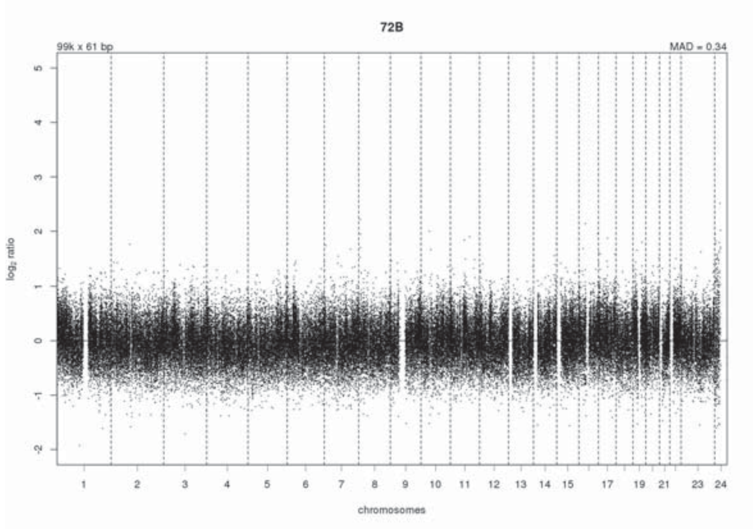
72B
99k x 61 bp
MAD = 0.34
log2 ratio
chromosomes

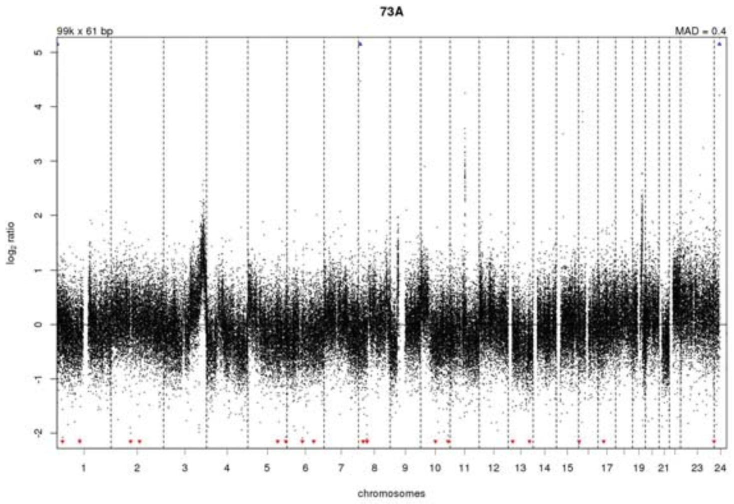
73A
99k x 61 bp
MAD = 0.4
log2 ratio
chromosomes

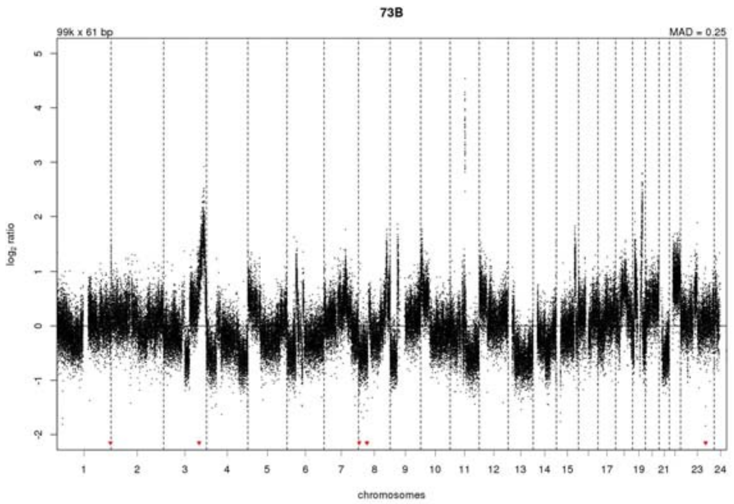
73B
99k x 61 bp
MAD = 0.25
log2 ratio
chromosomes

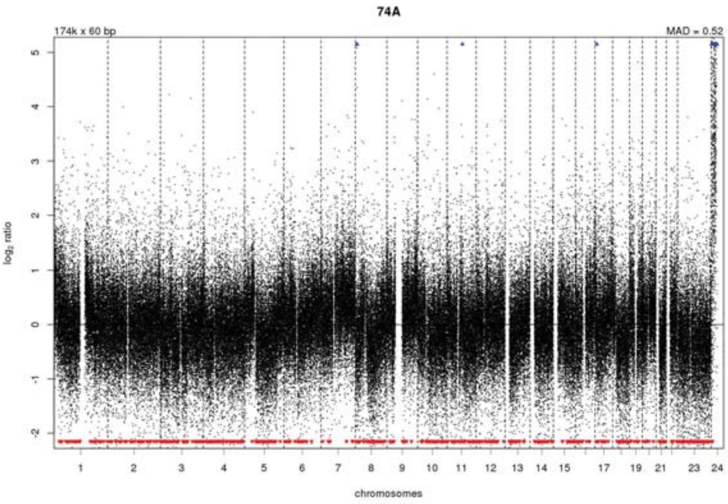
74A
174k x 60 bp
MAD = 0.52
log2 ratio
chromosomes

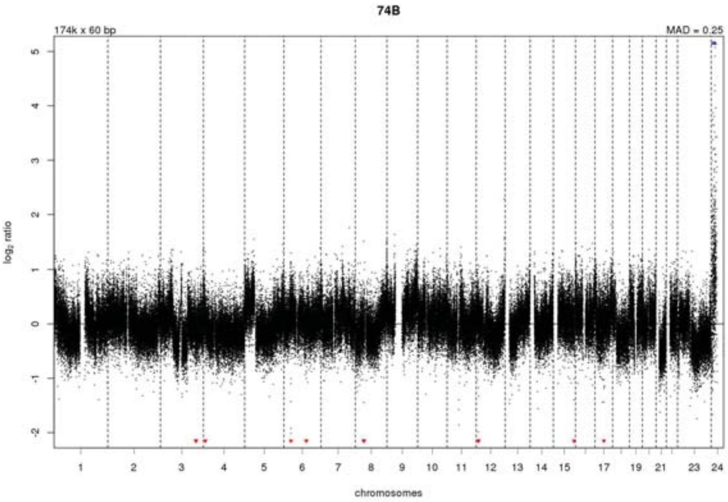
74B
174k x 60 bp
MAD = 0.25
log2 ratio
chromosomes

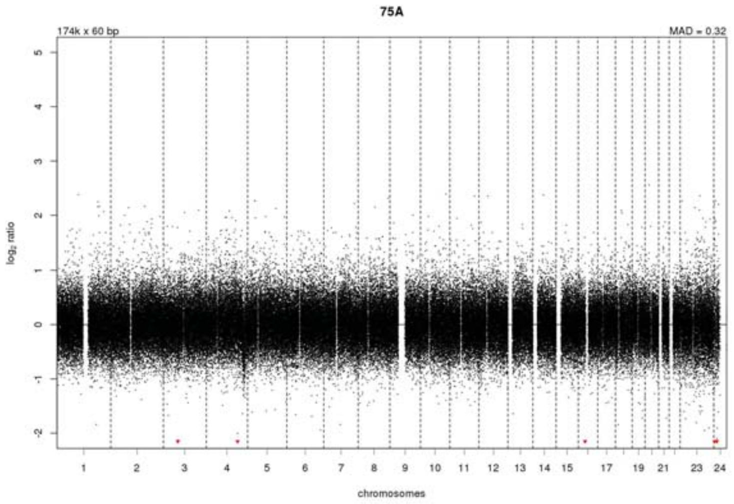
75A
174k x 60 bp
MAD = 0.32
log2 ratio
chromosomes

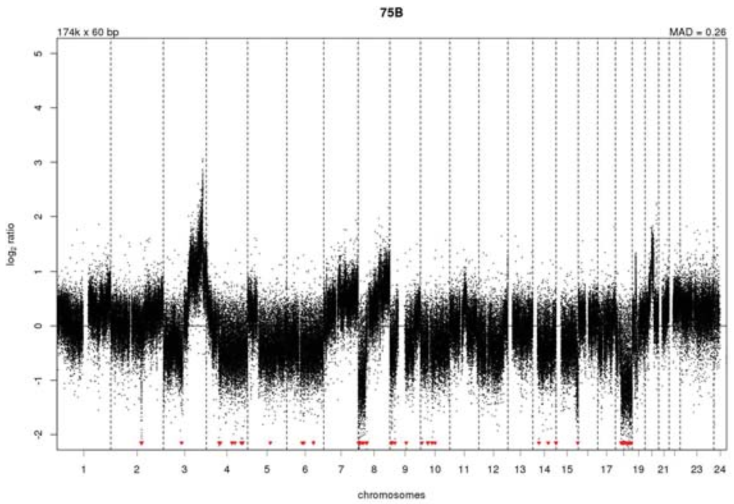
75B
174k x 60 bp
MAD = 0.26
log2 ratio
chromosomes

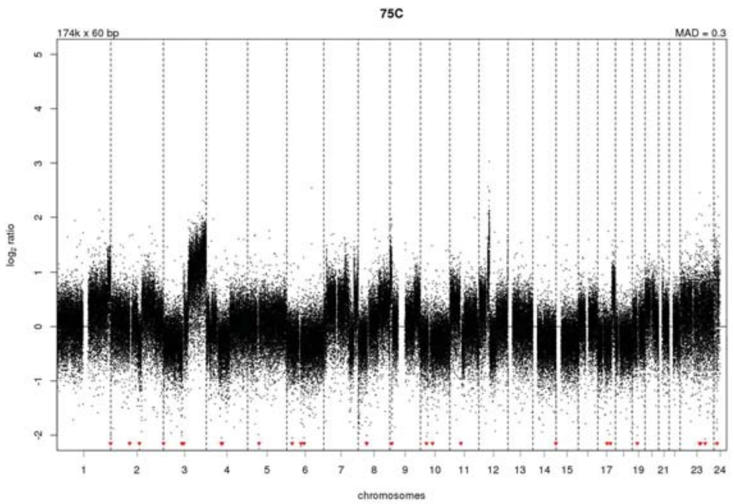
75C
174k x 60 bp
MAD = 0.3
log2 ratio
chromosomes

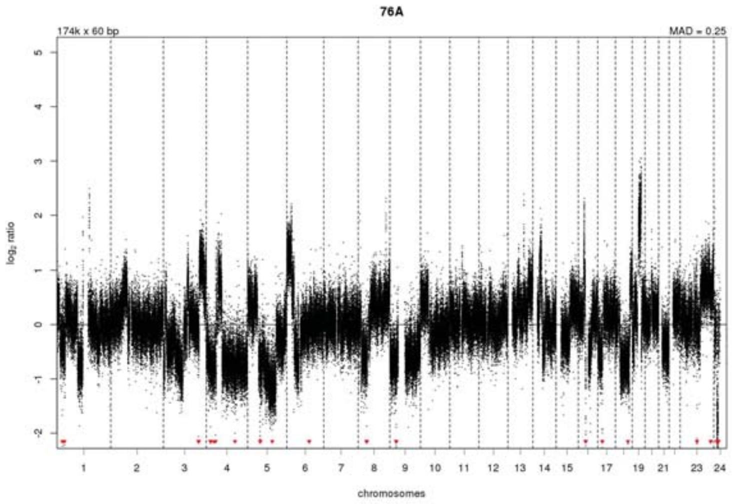
76A
174k x 60 bp
MAD = 0.25
log2 ratio
chromosomes
1
2
3
4
5
6
7
8
9
10
11
12
13
14
15
17
19
21
23
24

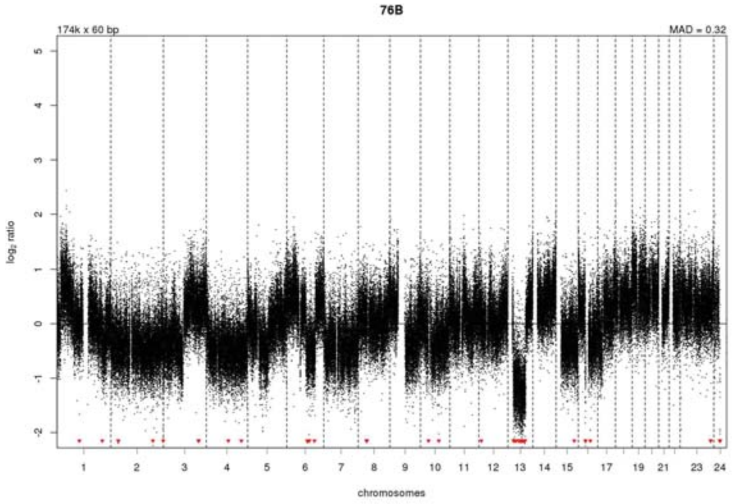
76B
174k x 60 bp
MAD = 0.32
log2 ratio
chromosomes
1
2
3
4
5
6
7
8
9
10
11
12
13
14
15
17
19
21
23
24

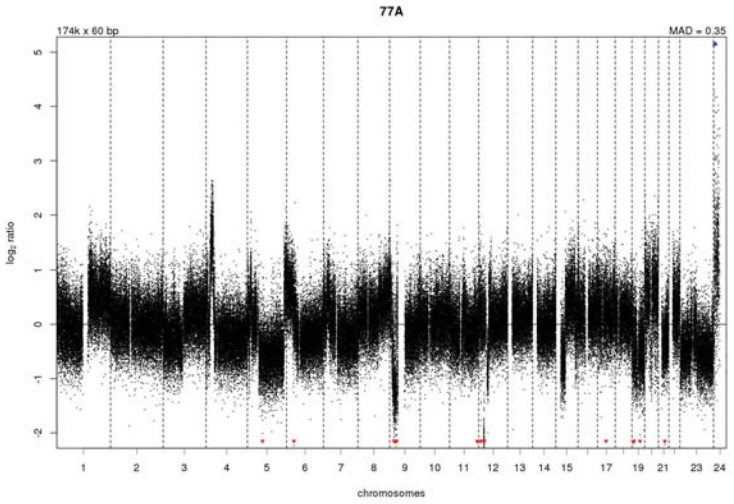

77A
174k x 60 bp
MAD = 0.35
log2 ratio
chromosomes

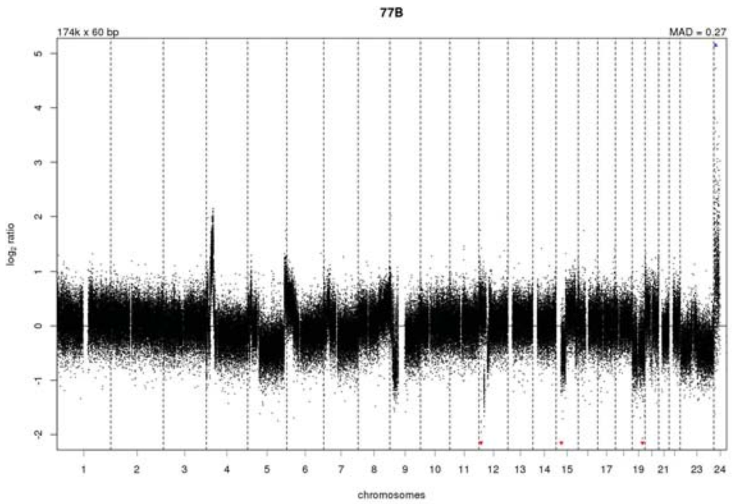

77B
174k x 60 bp
MAD = 0.27
log2 ratio
chromosomes

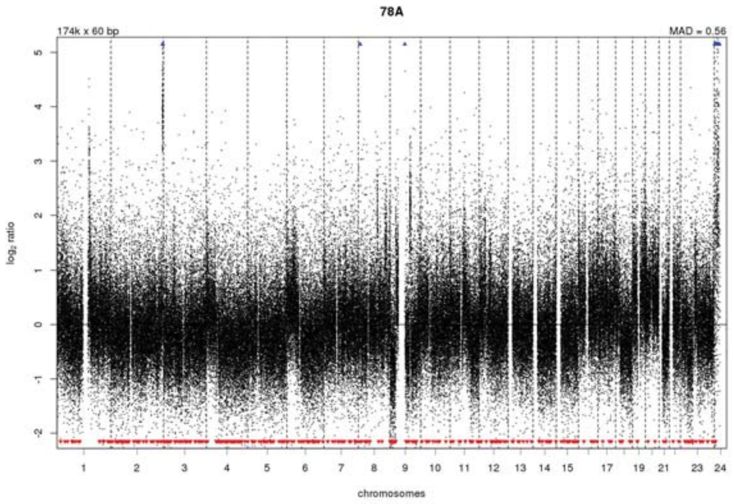
78A
174k x 60 bp
MAD = 0.56
log2 ratio
chromosomes

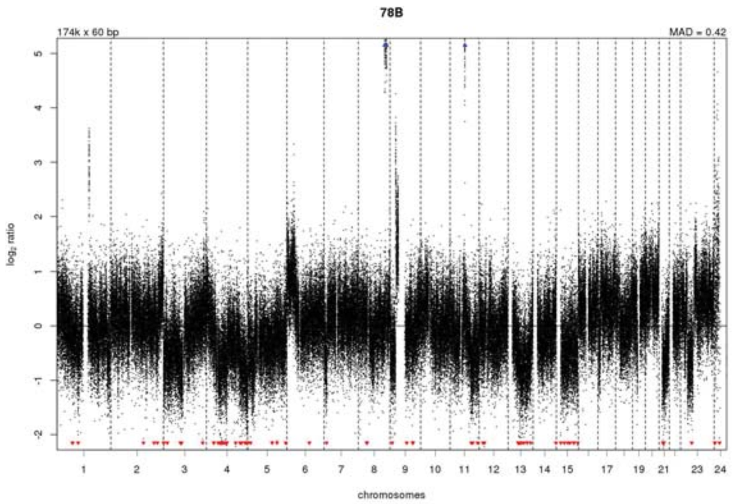
78B
174k x 60 bp
MAD = 0.42
log2 ratio
chromosomes

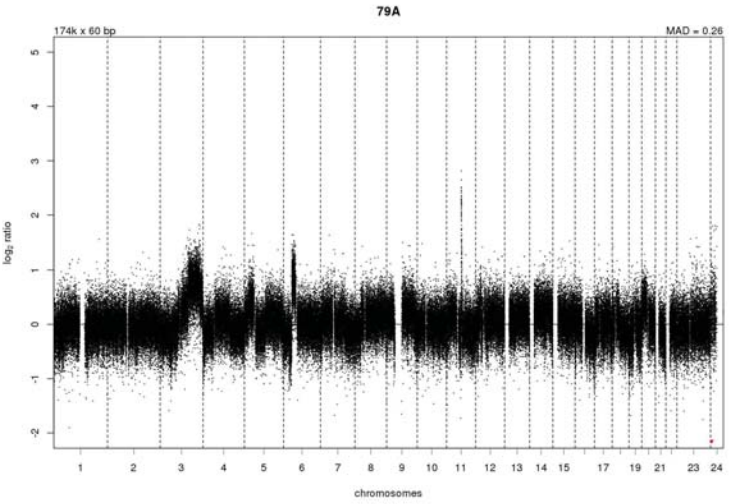
79A
174k x 60 bp
MAD = 0.26
log2 ratio
chromosomes

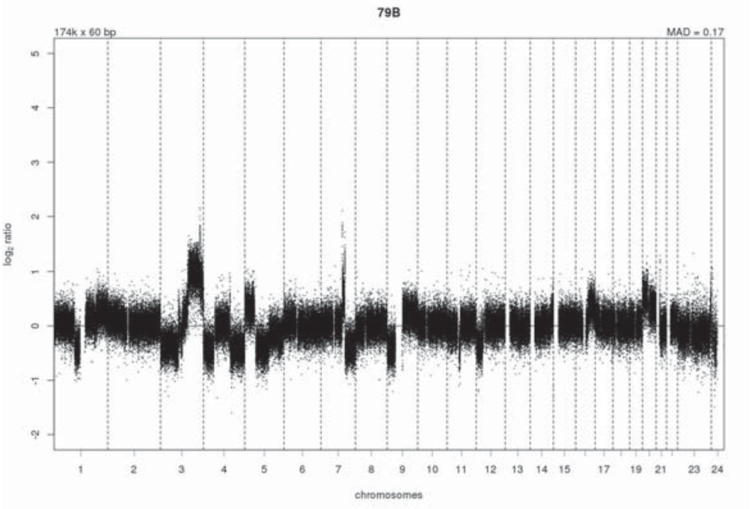
79B
174k x 60 bp
MAD = 0.17
log2 ratio
chromosomes

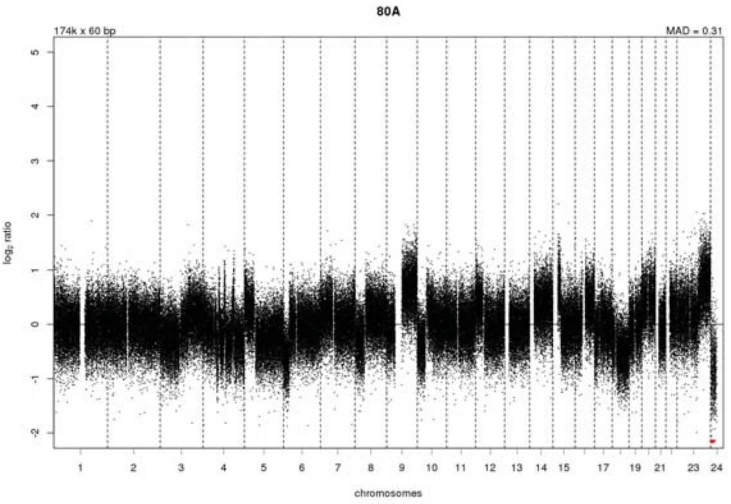
80A
174k x 60 bp
MAD = 0.31
log2 ratio
5
4
3
2
1
0
-1
-2
1
2
3
4
5
6
7
8
9
10
11
12
13
14
15
17
19
21
23
24
chromosomes

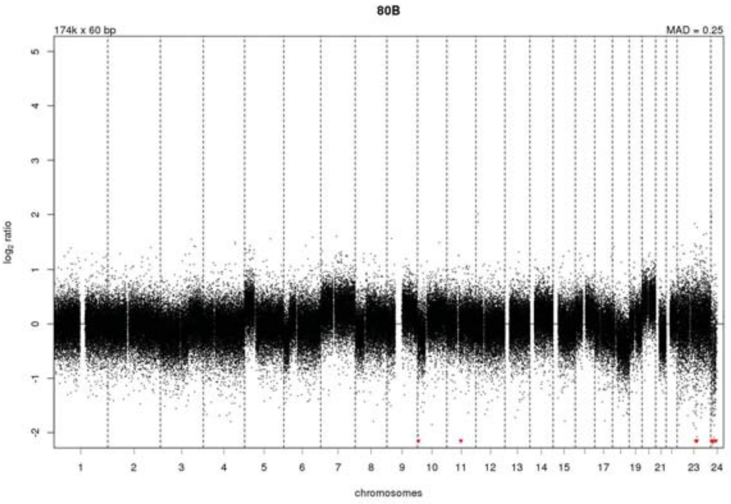
80B
174k x 60 bp
MAD = 0.25
log2 ratio
5
4
3
2
1
0
-1
-2
1
2
3
4
5
6
7
8
9
10
11
12
13
14
15
17
19
21
23
24
chromosomes

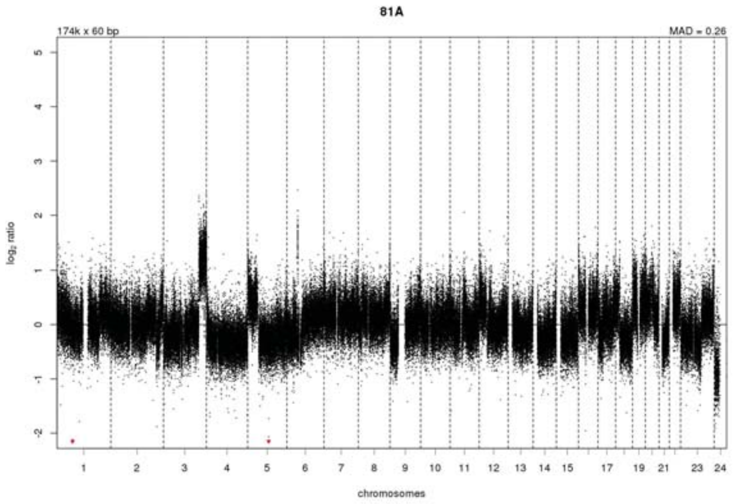
81A
174k x 60 bp
MAD = 0.26
log2 ratio
chromosomes

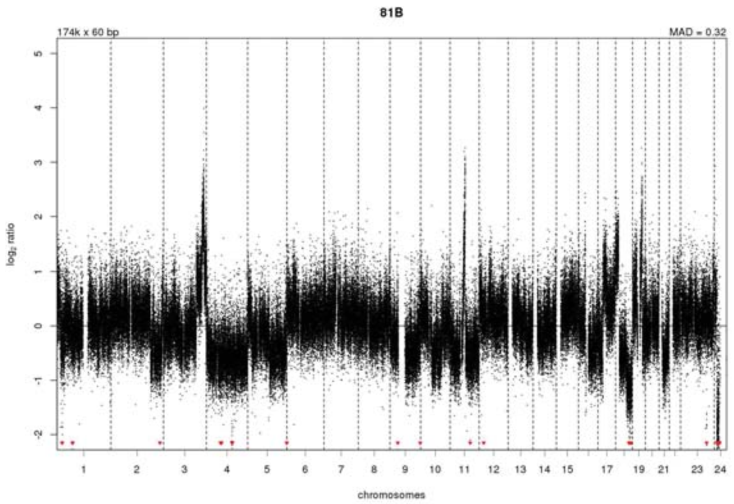
81B
174k x 60 bp
MAD = 0.32
log2 ratio
chromosomes

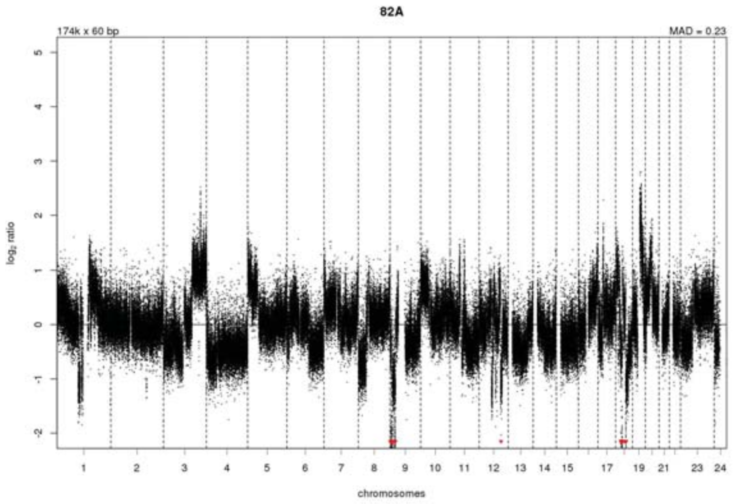
82A
174k x 60 bp
MAD = 0.23
log2 ratio
chromosomes
1 2 3 4 5 6 7 8 9 10 11 12 13 14 15 17 19 21 23 24

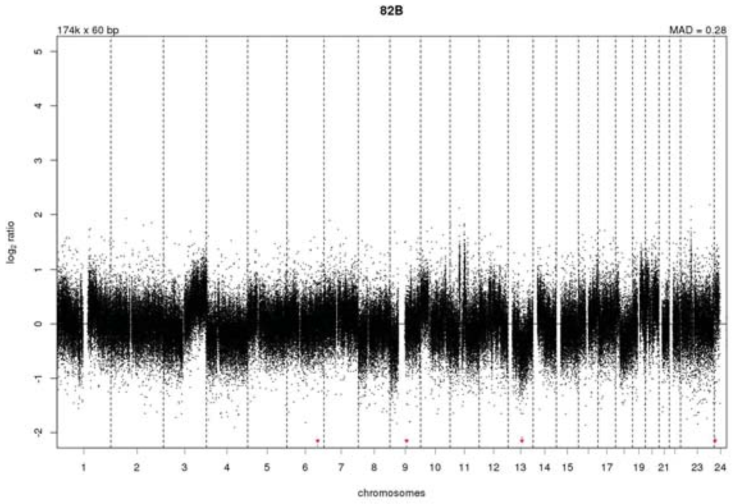
82B
174k x 60 bp
MAD = 0.28
log2 ratio
chromosomes
1 2 3 4 5 6 7 8 9 10 11 12 13 14 15 17 19 21 23 24

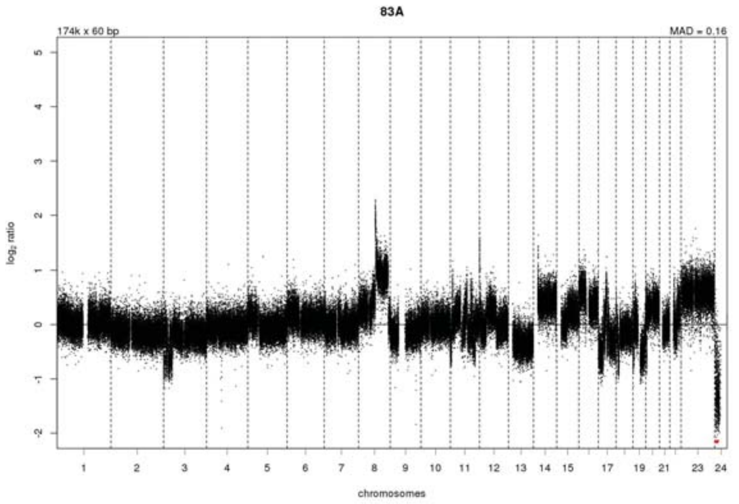
83A
174k x 60 bp
MAD = 0.16
log2 ratio
5 4 3 2 1 0 -1 -2
1 2 3 4 5 6 7 8 9 10 11 12 13 14 15 17 19 21 23 24
chromosomes

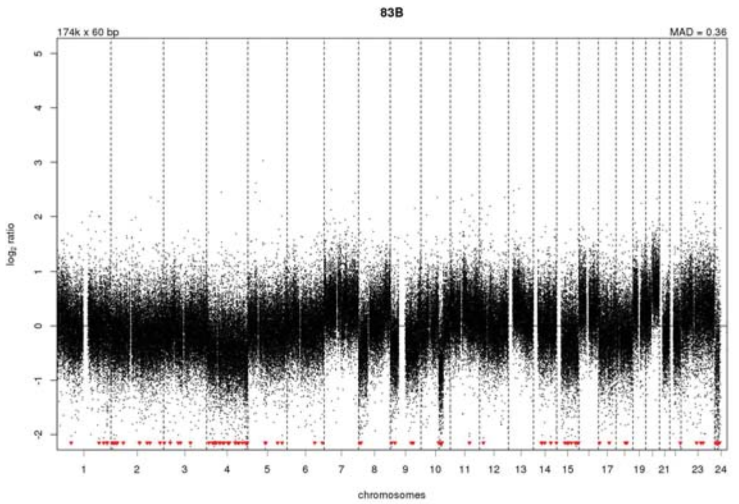
83B
174k x 60 bp
MAD = 0.36
log2 ratio
5 4 3 2 1 0 -1 -2
1 2 3 4 5 6 7 8 9 10 11 12 13 14 15 17 19 21 23 24
chromosomes

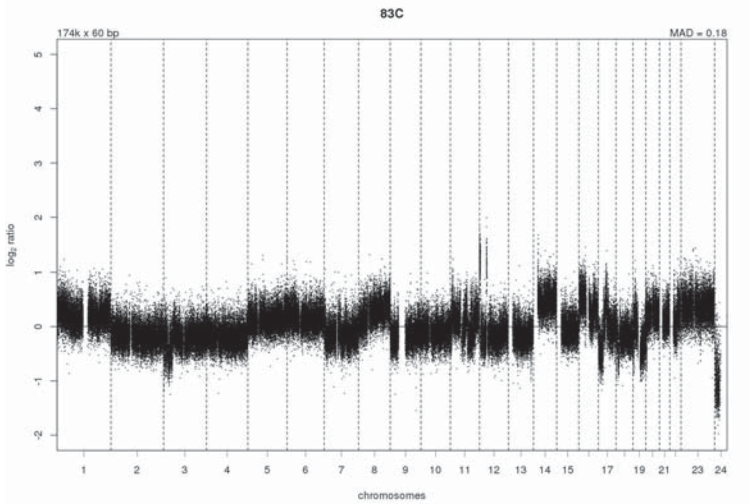
83C
174k x 60 bp
MAD = 0.18
log2 ratio
5 4 3 2 1 0 -1 -2
1 2 3 4 5 6 7 8 9 10 11 12 13 14 15 17 19 21 23 24
chromosomes

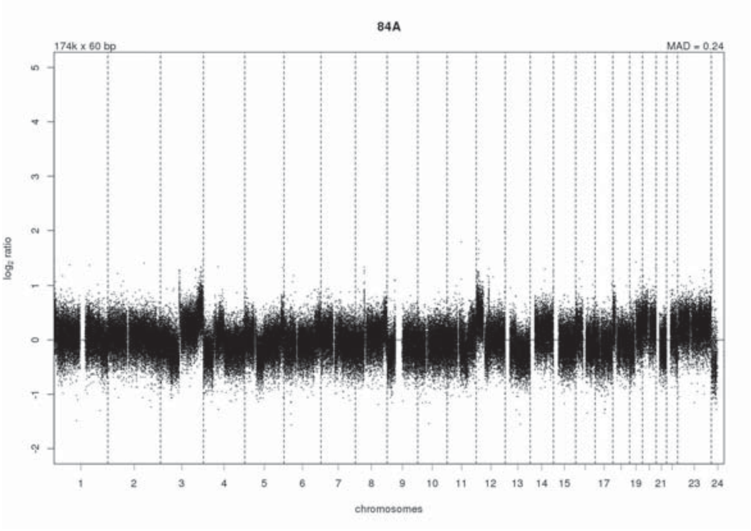
84A
174k x 60 bp
MAD = 0.24
log2 ratio
chromosomes

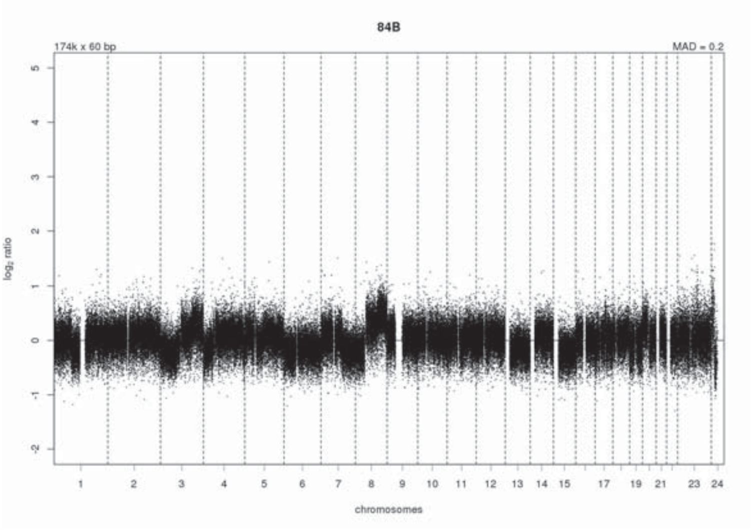
84B
174k x 60 bp
MAD = 0.2
log2 ratio
chromosomes

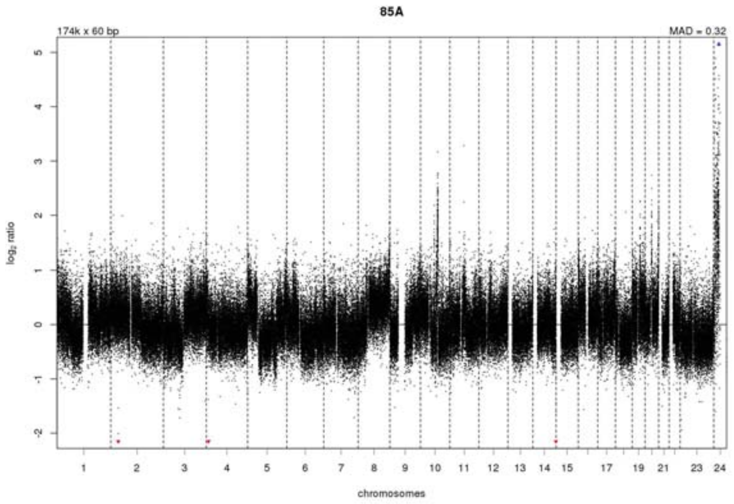
85A
174k x 60 bp
MAD = 0.32
log2 ratio
chromosomes
1 2 3 4 5 6 7 8 9 10 11 12 13 14 15 17 19 21 23 24

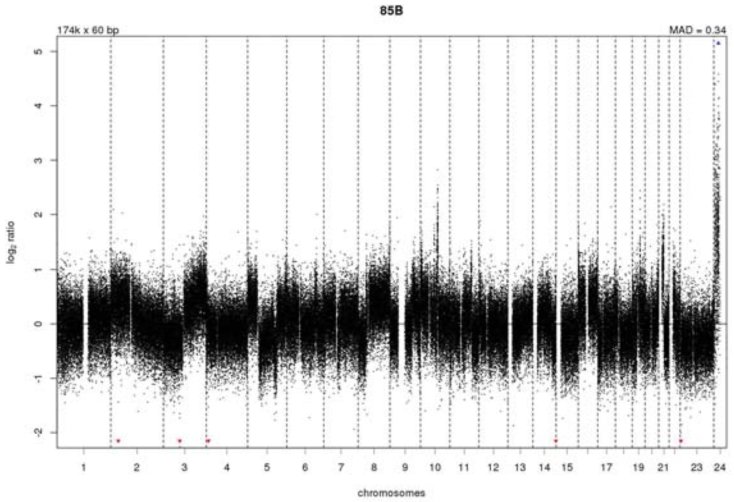
85B
174k x 60 bp
MAD = 0.34
log2 ratio
chromosomes
1 2 3 4 5 6 7 8 9 10 11 12 13 14 15 17 19 21 23 24

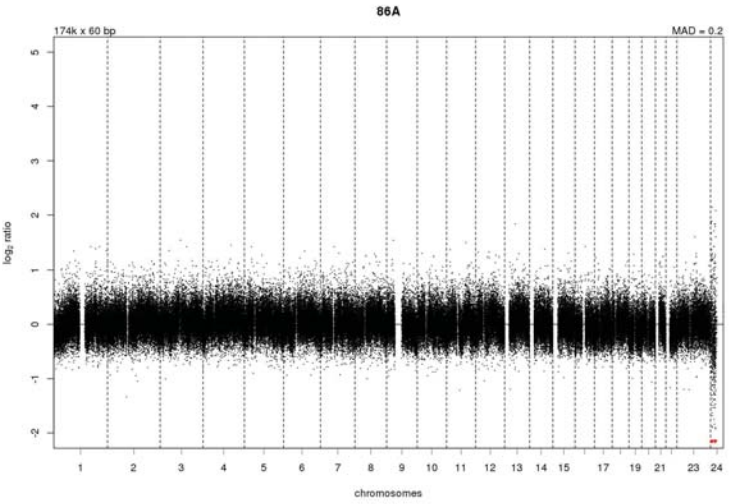

86A
174k x 60 bp
MAD = 0.2
log2 ratio
chromosomes

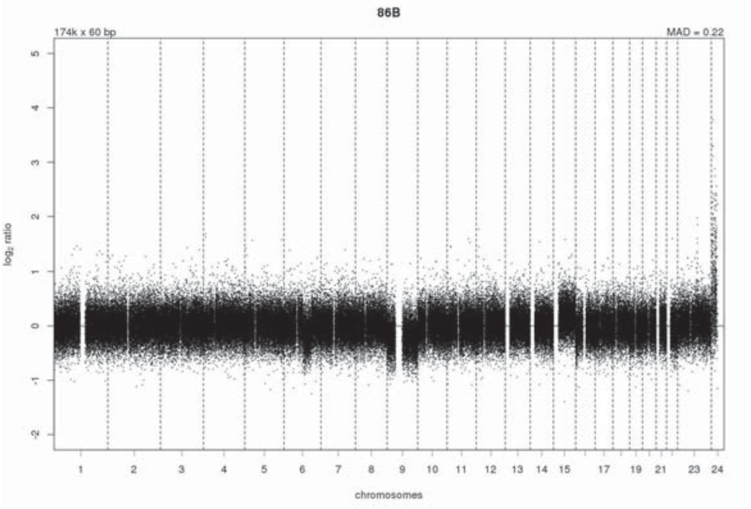

86B
174k x 60 bp
MAD = 0.22
log2 ratio
chromosomes

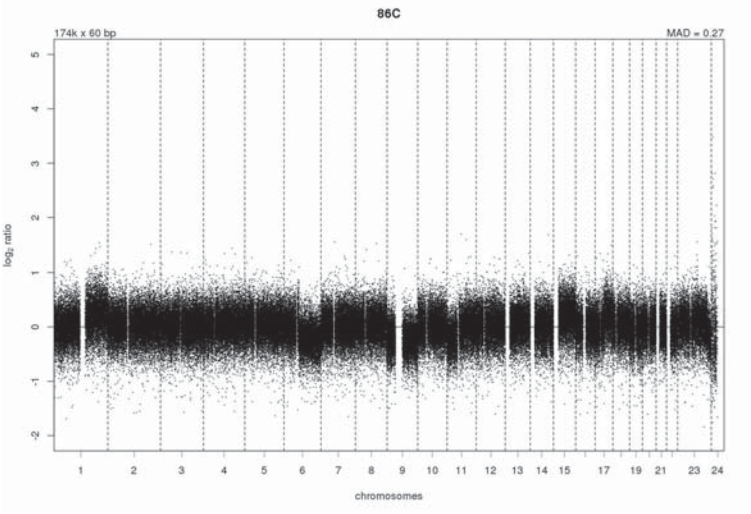

86C
174k x 60 bp
MAD = 0.27
log2 ratio
chromosomes

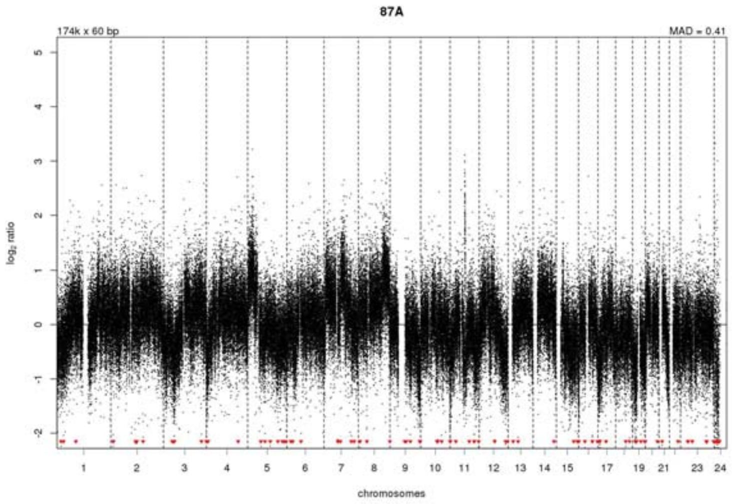
87A
174k x 60 bp
MAD = 0.41
log2 ratio
chromosomes

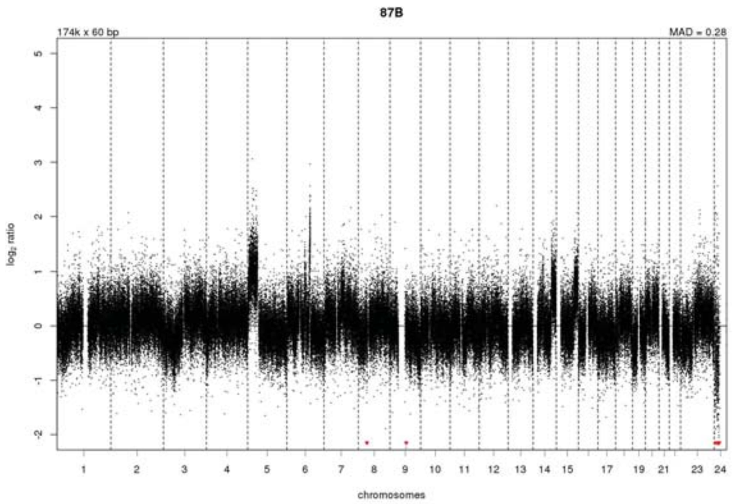
87B
174k x 60 bp
MAD = 0.28
log2 ratio
chromosomes

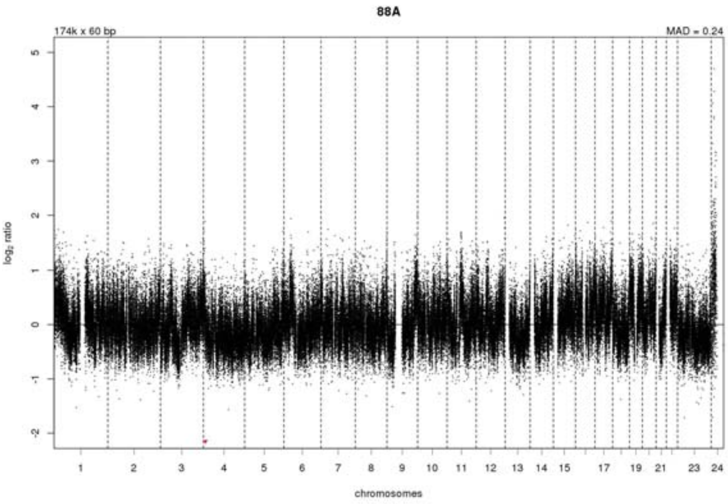
88A
174k x 60 bp
MAD = 0.24
log2 ratio
5
4
3
2
1
0
-1
-2
1 2 3 4 5 6 7 8 9 10 11 12 13 14 15 17 19 21 23 24
chromosomes

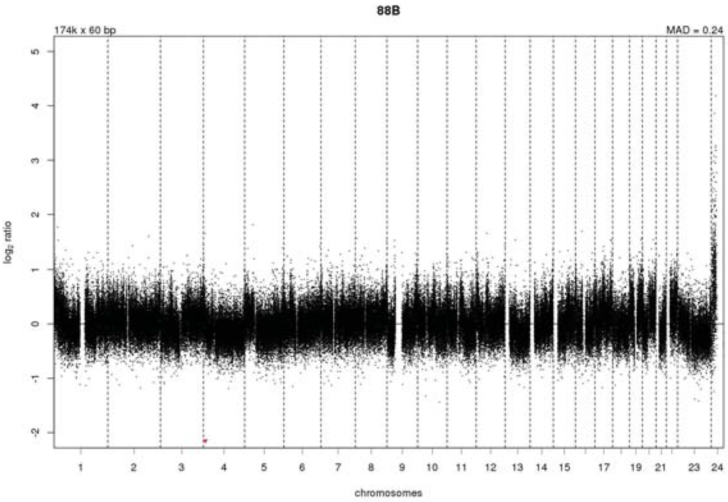
88B
174k x 60 bp
MAD = 0.24
log2 ratio
5
4
3
2
1
0
-1
-2
1 2 3 4 5 6 7 8 9 10 11 12 13 14 15 17 19 21 23 24
chromosomes

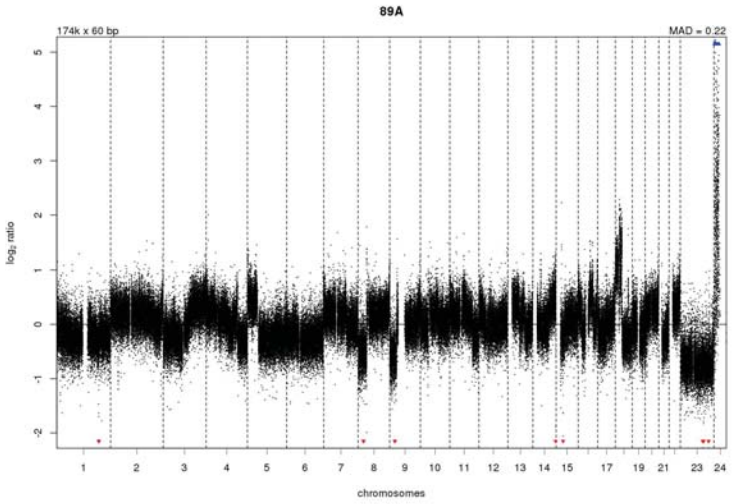
89A
174k x 60 bp
MAD = 0.22
log2 ratio
chromosomes

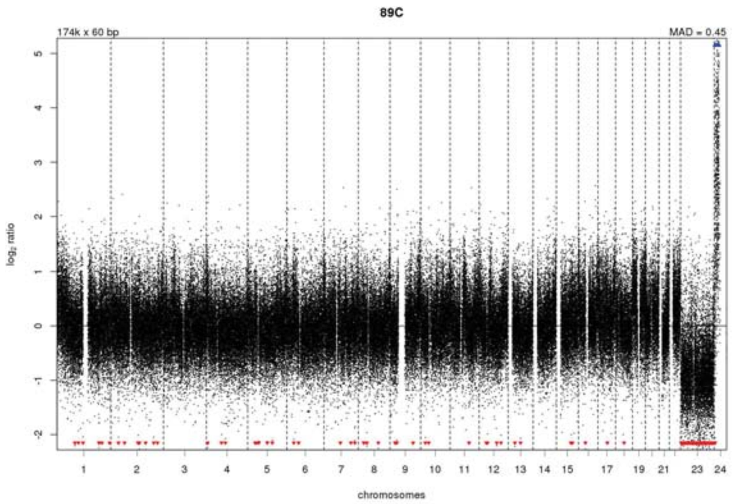
89C
174k x 60 bp
MAD = 0.45
log2 ratio
chromosomes

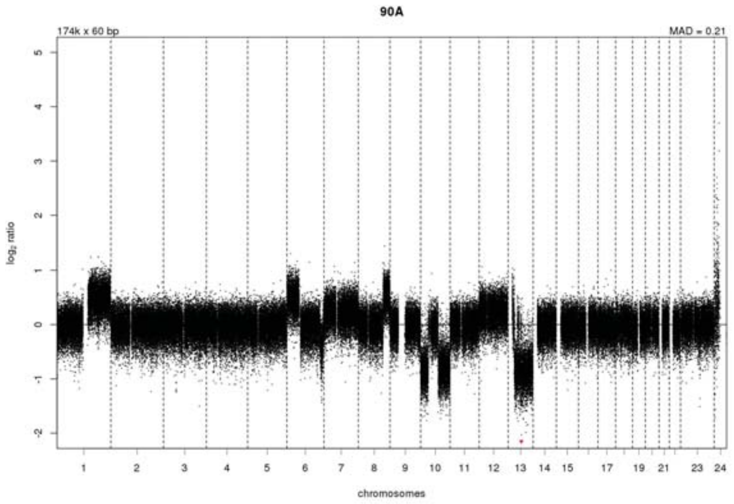
90A
174k x 60 bp
MAD = 0.21
log2 ratio
5
4
3
2
1
0
-1
-2
1
2
3
4
5
6
7
8
9
10
11
12
13
14
15
17
19
21
23
24
chromosomes

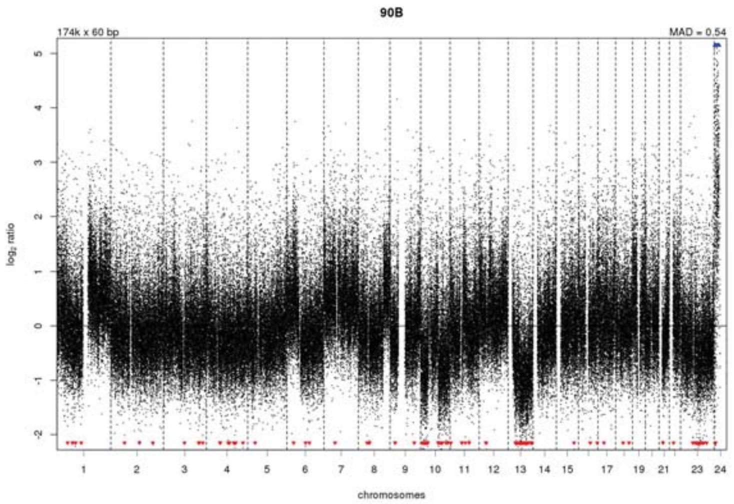
90B
174k x 60 bp
MAD = 0.54
log2 ratio
5
4
3
2
1
0
-1
-2
1
2
3
4
5
6
7
8
9
10
11
12
13
14
15
17
19
21
23
24
chromosomes
